# Supplementary material for: Exploring intra‐ and intergenomic variation in haplotype‐resolved pangenomes
Source: Plant Biotechnol J. 2025 Jan 5;23(3):874–86. doi: 10.1111/pbi.14545 (PMC11869183; doi:10.1111/pbi.14545)
Supplement: Supplementary file 3 — Supplement S3 Supplementary Data. [file PBI-23-874-s002.zip › Supplementary_data/sequence_visualization/README.pdf]

Exploring intra- and inter-genomic variation in haplotype-resolved pangenomes

PanTools ‘sequence_visualization’ output

This supplementary file holds two folders containing plots generated by PanTools ‘sequence_visualization’ function for all haplotype-resolved genome assemblies.

- Apple visualizations were generated by including the following rule settings: ‘gene_classification, ‘haplotype_presence’, ‘other_ chromosomes’, ‘repeat_coverage’, and ‘gene_coverage’. The ‘Coverage per block’ feature bar in the apple plots shows the percentage of the genome overlapping with gene and repeat annotations in 500 Kb blocks.
- Potato plots were created with only ‘haplotype_presence’ bars.

In both apple and potato, synteny relationships are drawn between haplotypes. These are included regardless of number of rules set. Figure 1 displays legends for the different type of annotation bars, which are universal and applicable to both apple and potato visualizations.

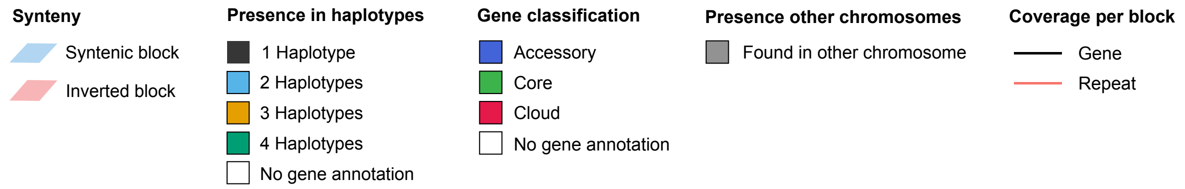

Fig. 1. Legends for different annotation bars
