# Supplementary figures and images for: Exploring intra‐ and intergenomic variation in haplotype‐resolved pangenomes
Source: Plant Biotechnol J. 2025 Jan 5;23(3):874–86. doi: 10.1111/pbi.14545 (PMC11869183; doi:10.1111/pbi.14545)

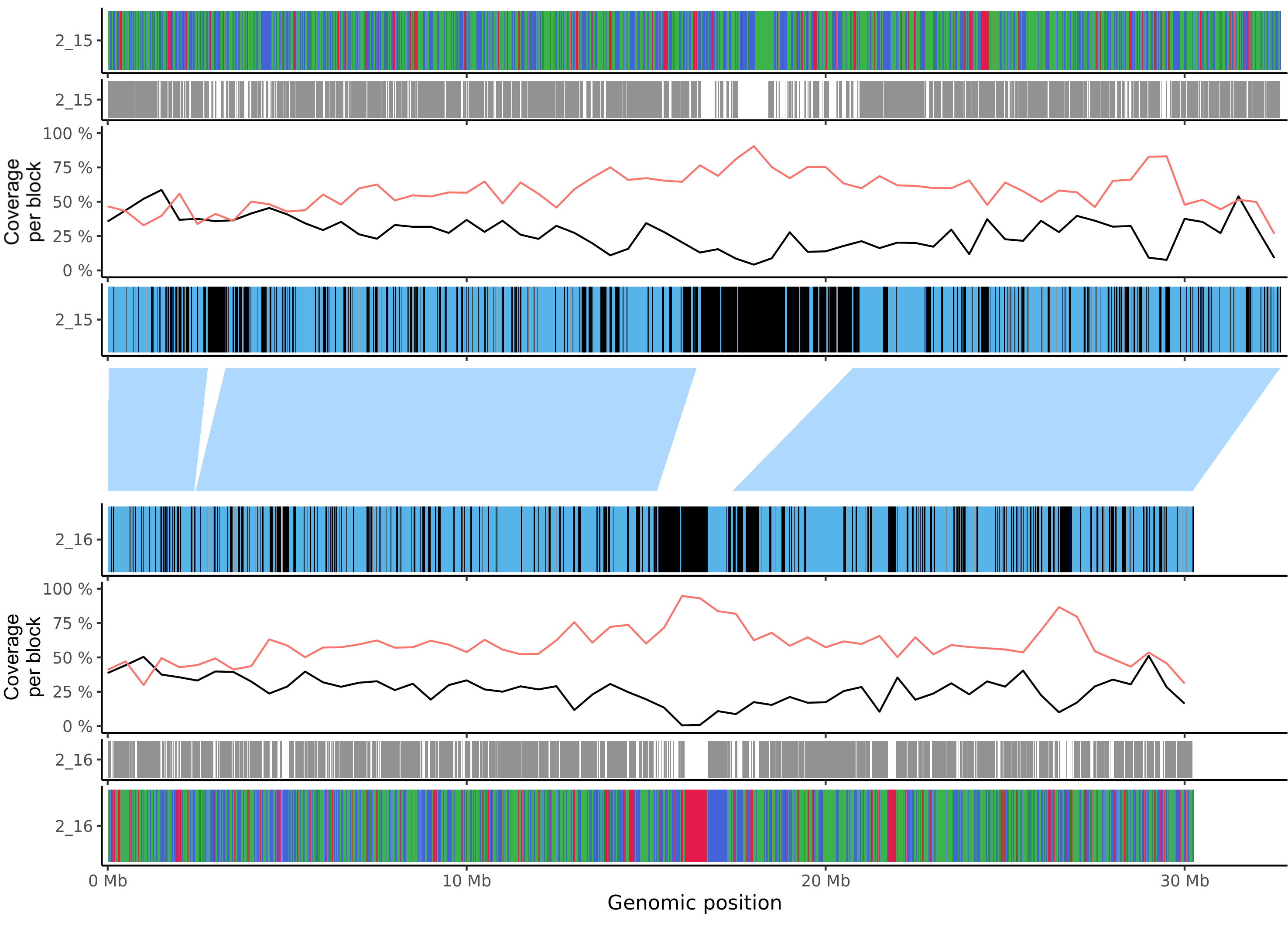

Supplement: Supplementary file 3 — Supplement S3 Supplementary Data. [file PBI-23-874-s002.zip › Supplementary_data/sequence_visualization/Apple/mdomestica_gala_chr_17.png]

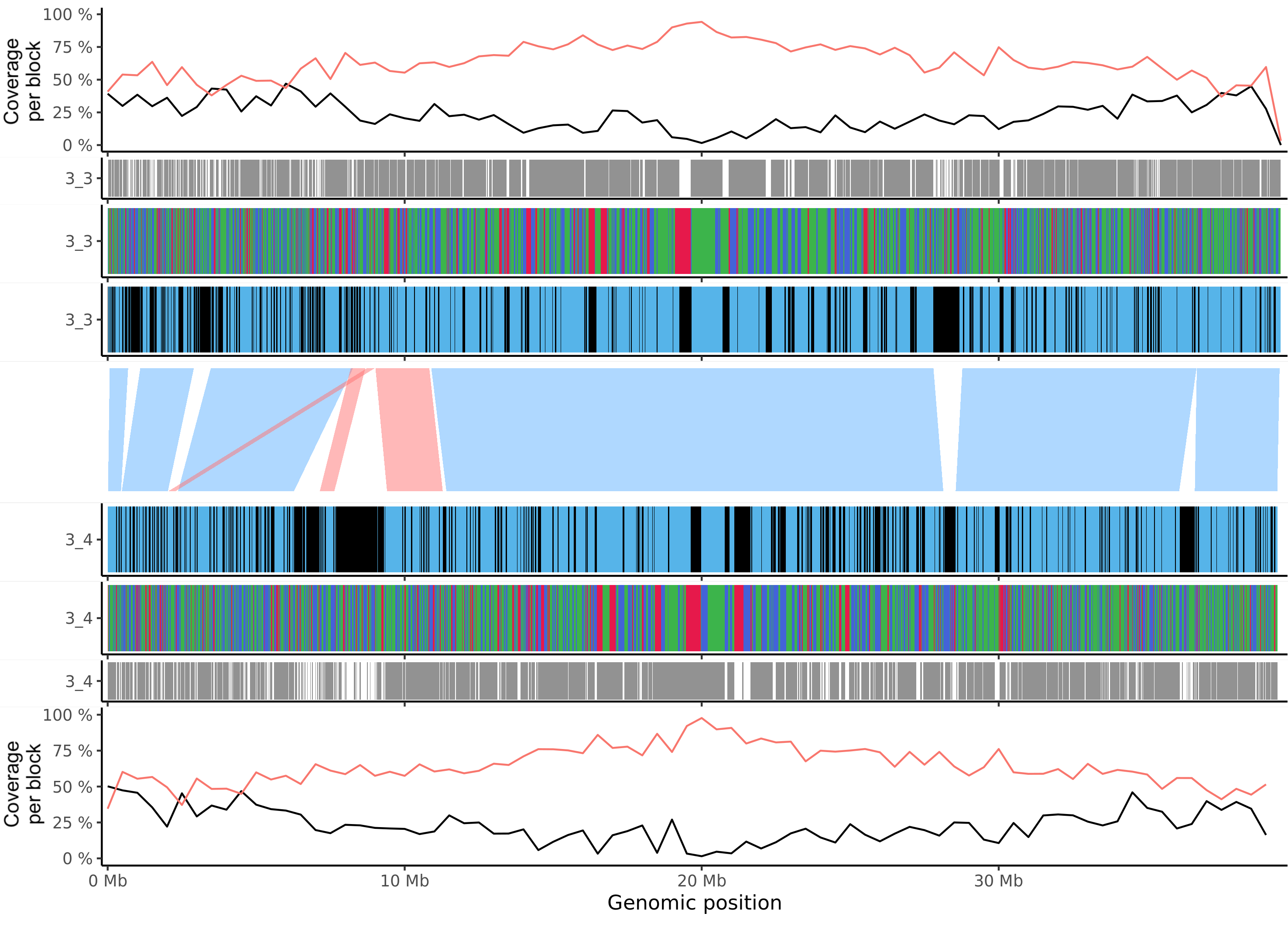

Supplement: Supplementary file 3 — Supplement S3 Supplementary Data. [file PBI-23-874-s002.zip › Supplementary_data/sequence_visualization/Apple/msieversii_chr_11.png]

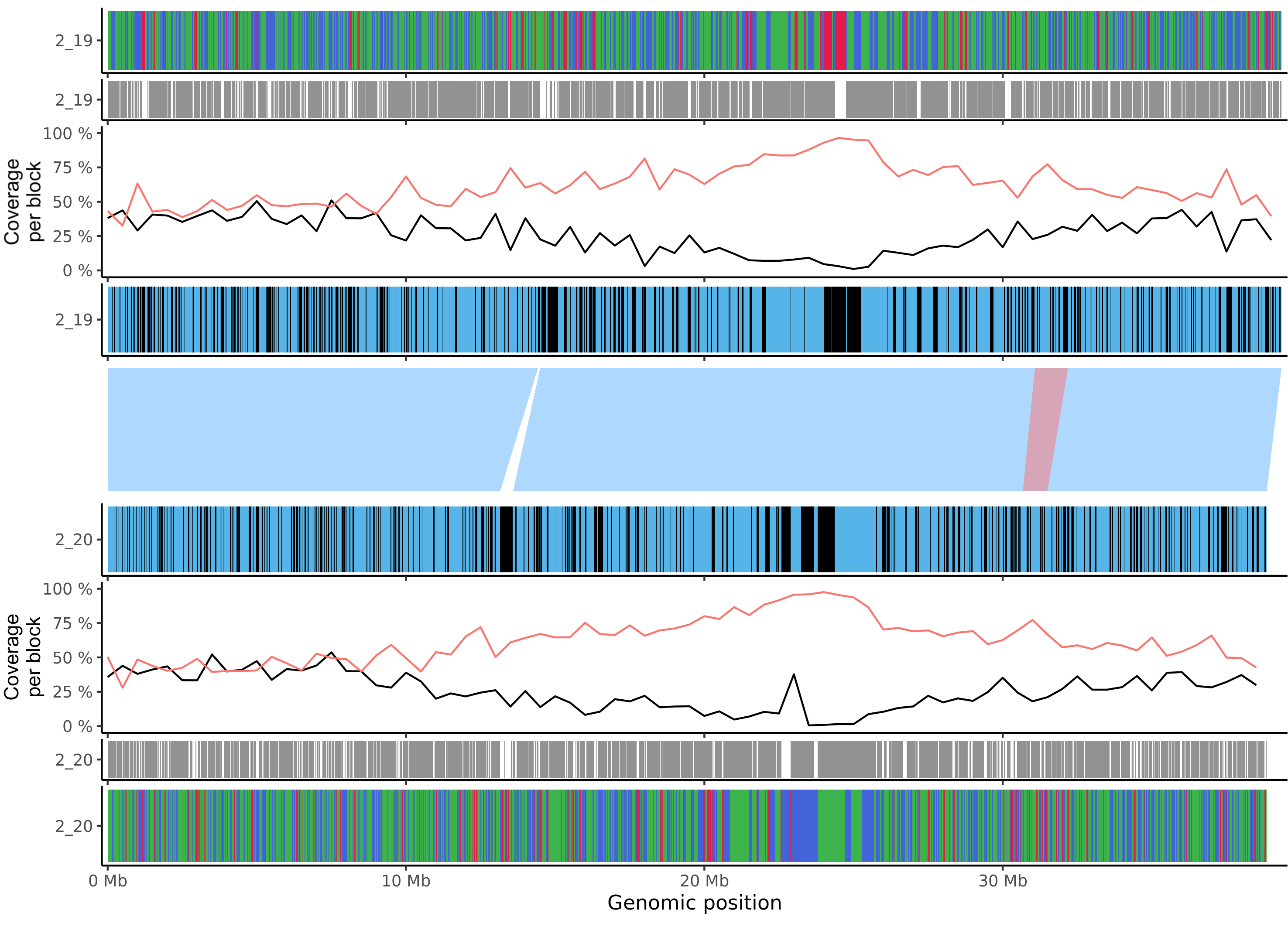

Supplement: Supplementary file 3 — Supplement S3 Supplementary Data. [file PBI-23-874-s002.zip › Supplementary_data/sequence_visualization/Apple/mdomestica_gala_chr_2.png]

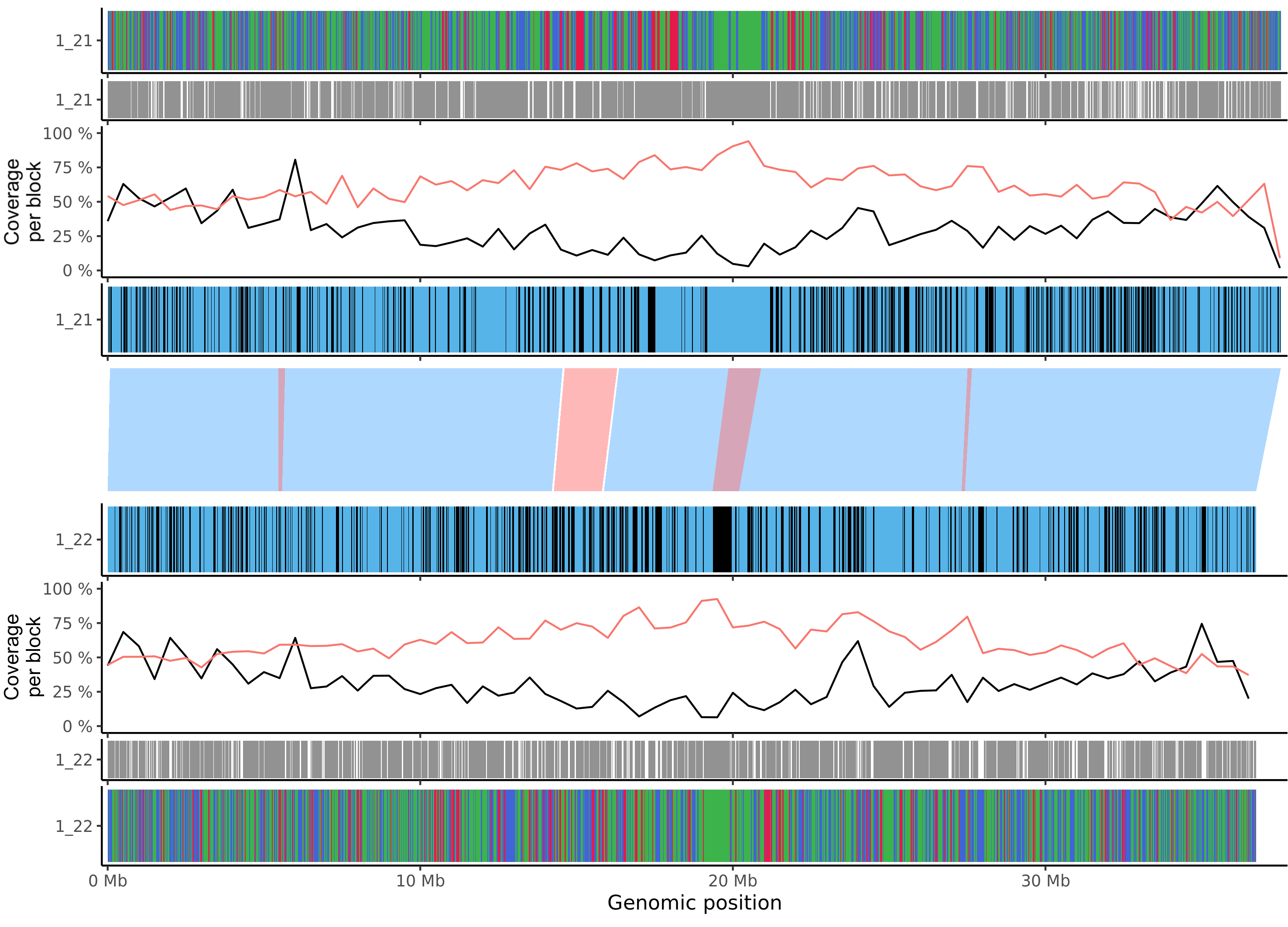

Supplement: Supplementary file 3 — Supplement S3 Supplementary Data. [file PBI-23-874-s002.zip › Supplementary_data/sequence_visualization/Apple/msylvestris_chr_3.png]

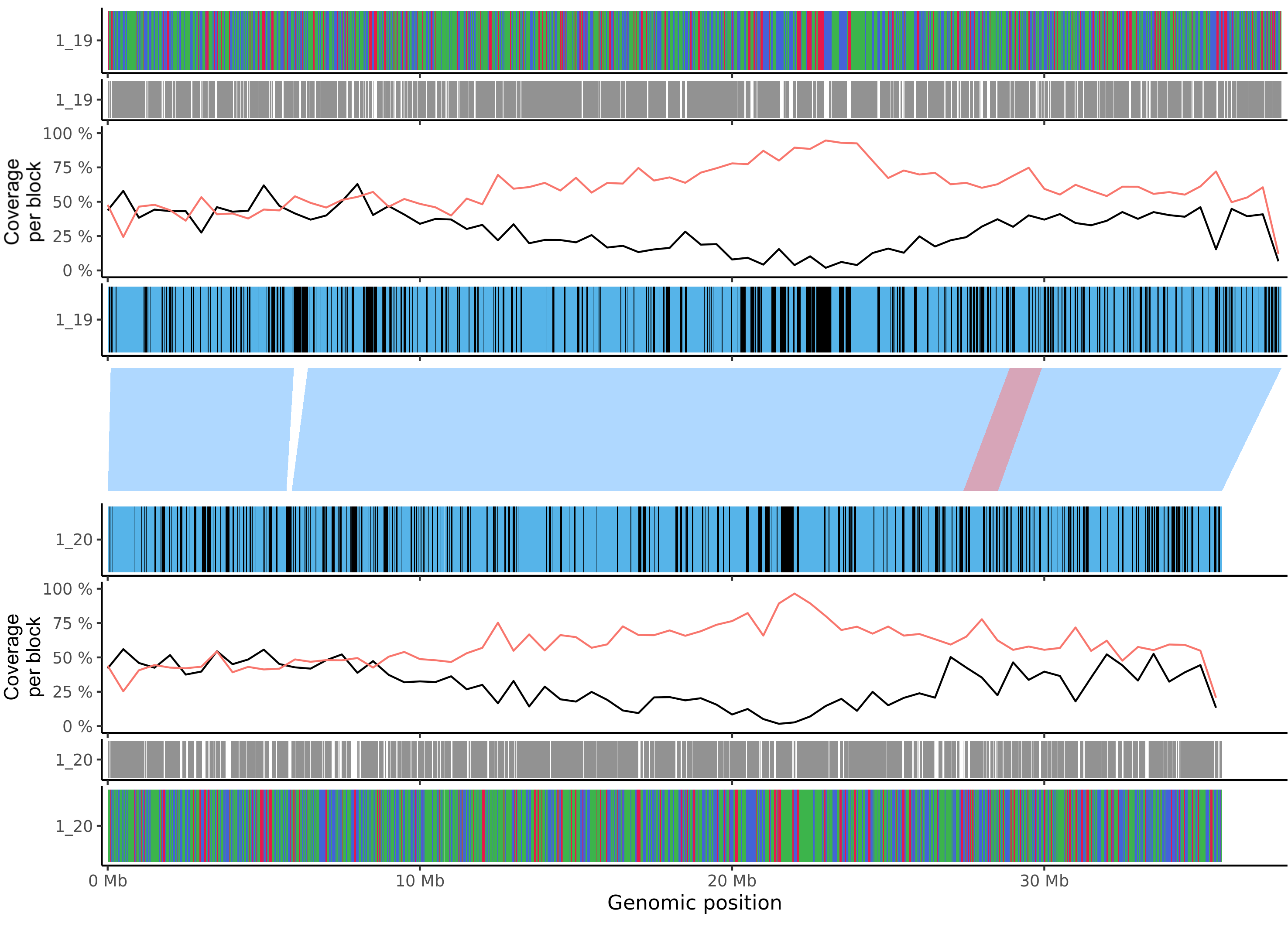

Supplement: Supplementary file 3 — Supplement S3 Supplementary Data. [file PBI-23-874-s002.zip › Supplementary_data/sequence_visualization/Apple/msylvestris_chr_2.png]

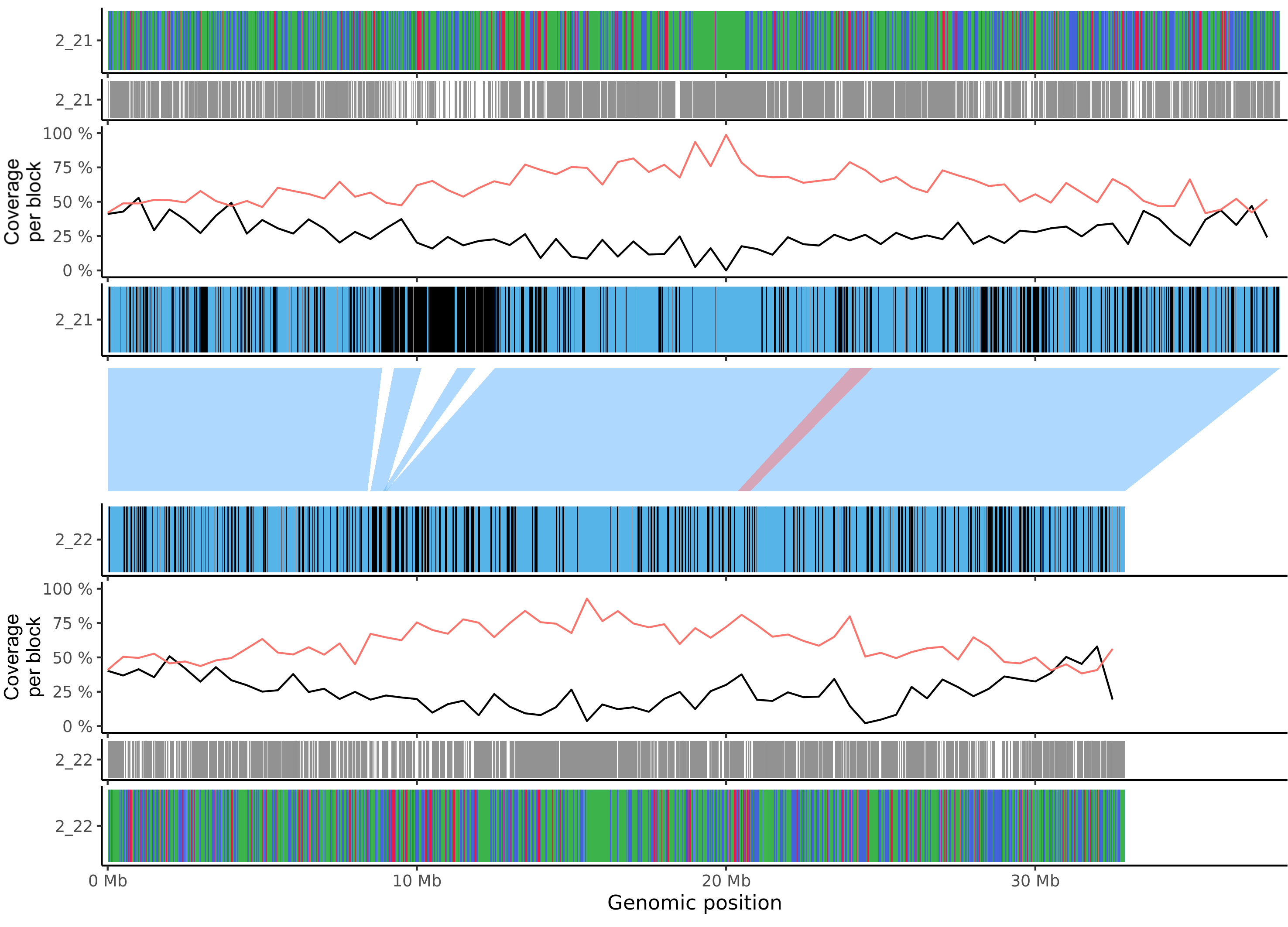

Supplement: Supplementary file 3 — Supplement S3 Supplementary Data. [file PBI-23-874-s002.zip › Supplementary_data/sequence_visualization/Apple/mdomestica_gala_chr_3.png]

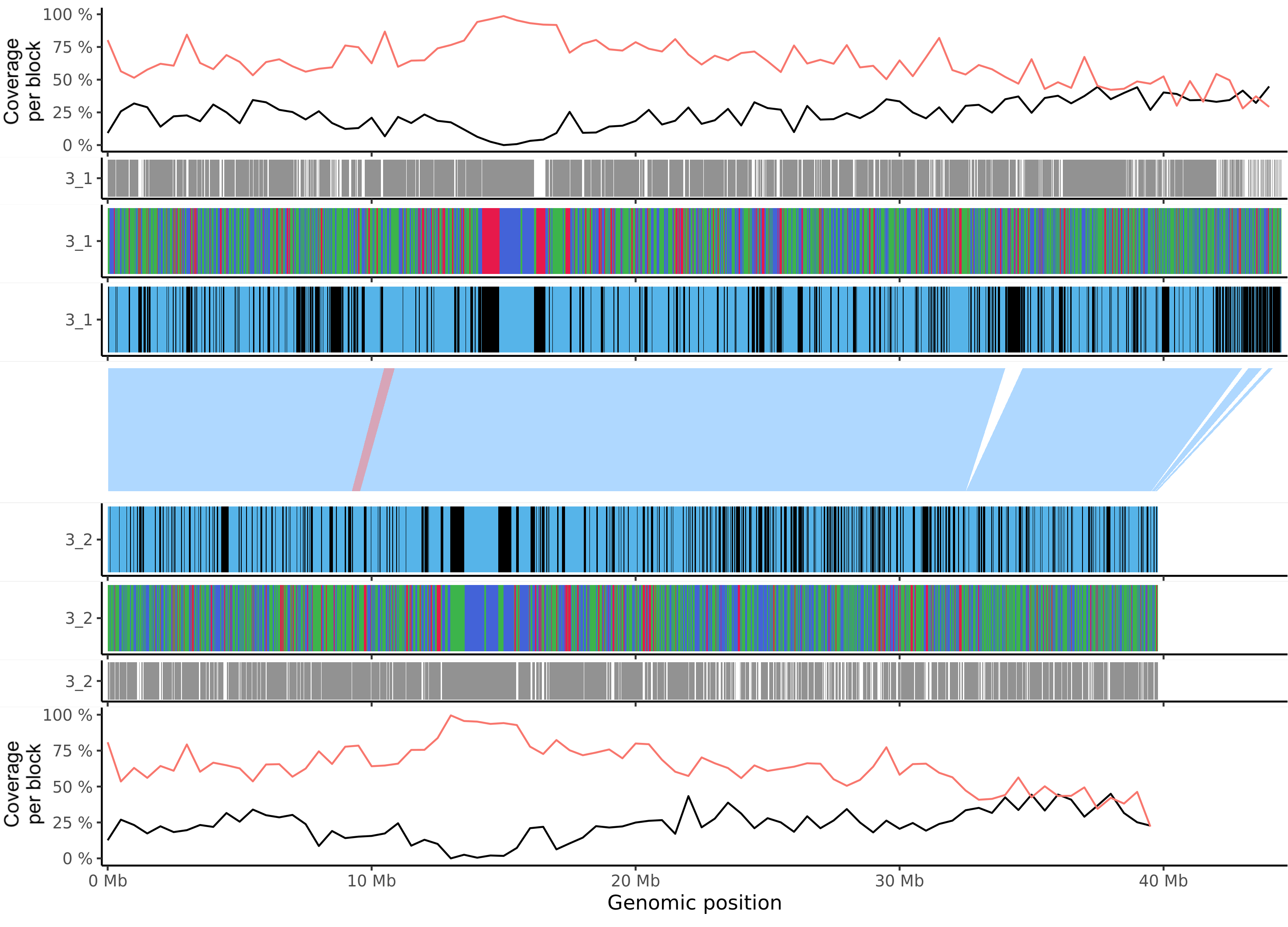

Supplement: Supplementary file 3 — Supplement S3 Supplementary Data. [file PBI-23-874-s002.zip › Supplementary_data/sequence_visualization/Apple/msieversii_chr_10.png]

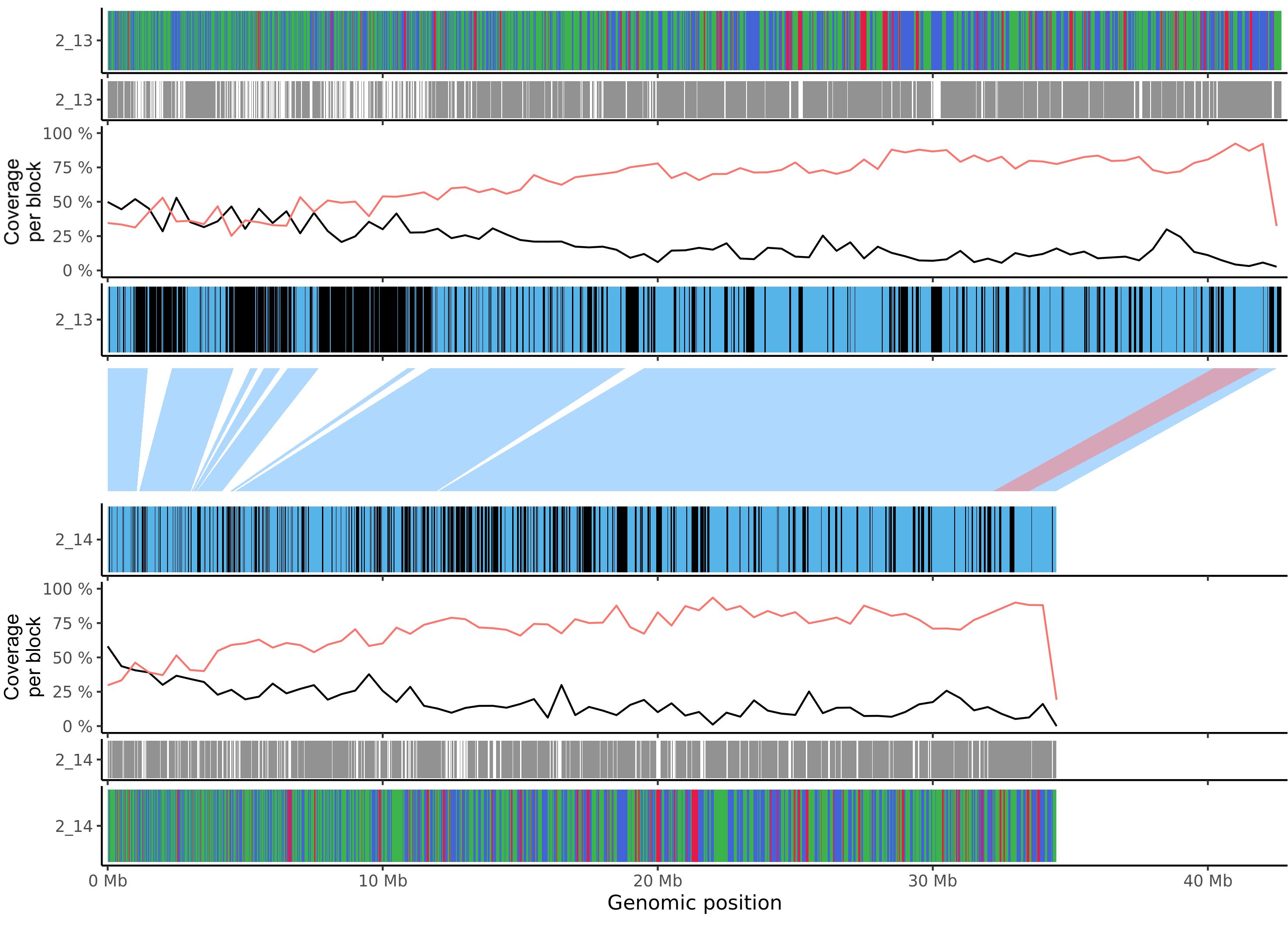

Supplement: Supplementary file 3 — Supplement S3 Supplementary Data. [file PBI-23-874-s002.zip › Supplementary_data/sequence_visualization/Apple/mdomestica_gala_chr_16.png]

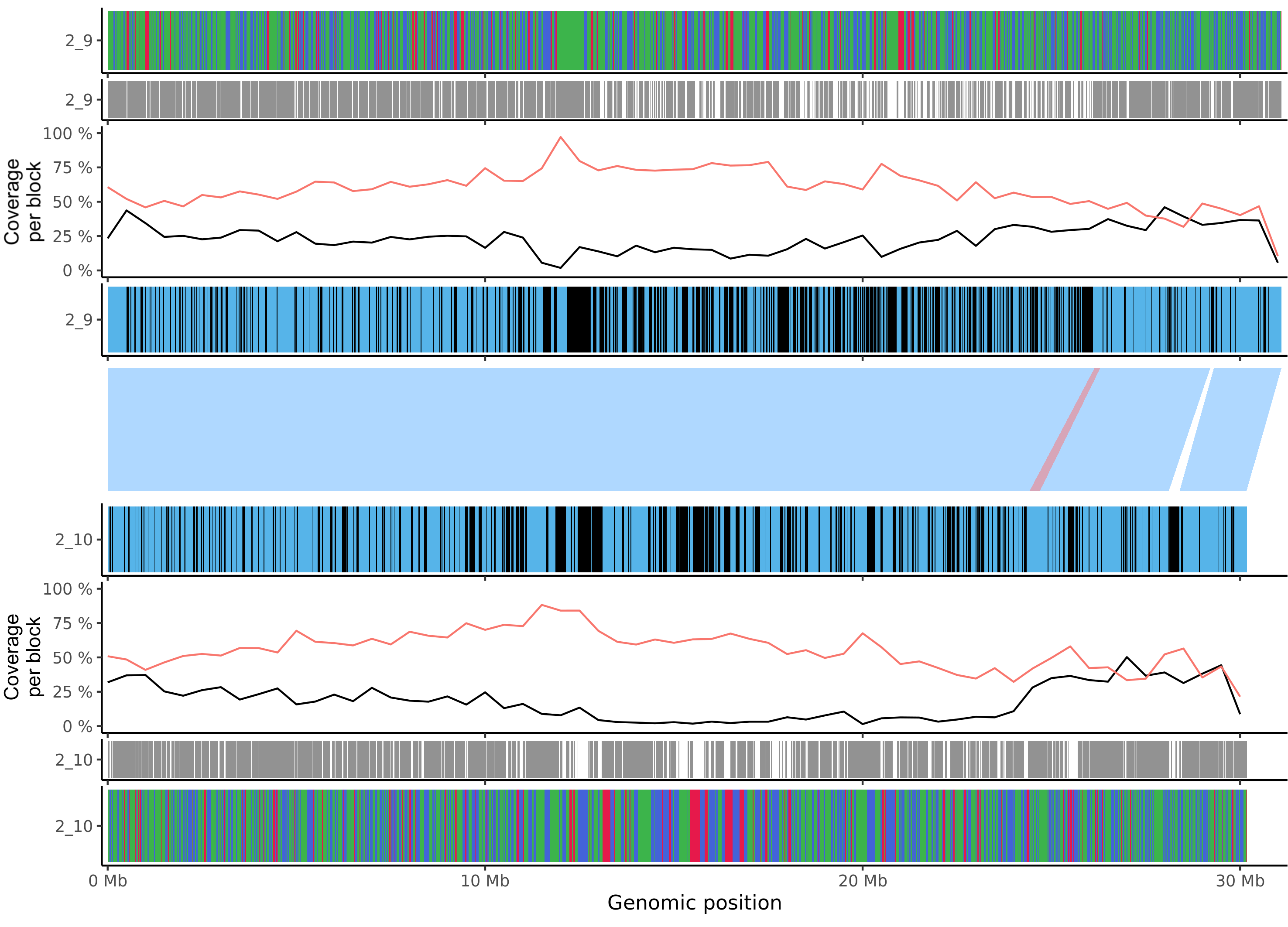

Supplement: Supplementary file 3 — Supplement S3 Supplementary Data. [file PBI-23-874-s002.zip › Supplementary_data/sequence_visualization/Apple/mdomestica_gala_chr_14.png]

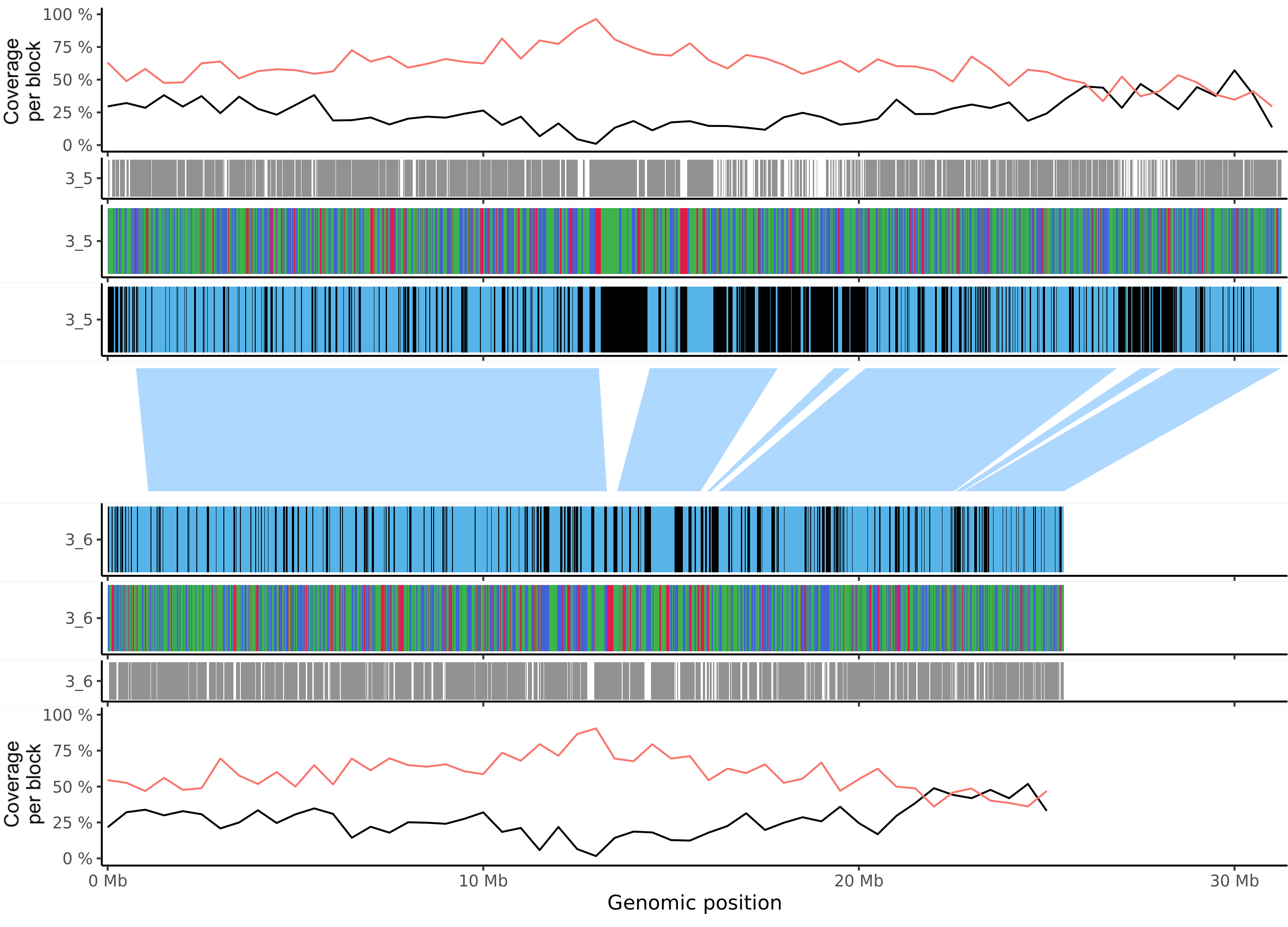

Supplement: Supplementary file 3 — Supplement S3 Supplementary Data. [file PBI-23-874-s002.zip › Supplementary_data/sequence_visualization/Apple/msieversii_chr_12.png]

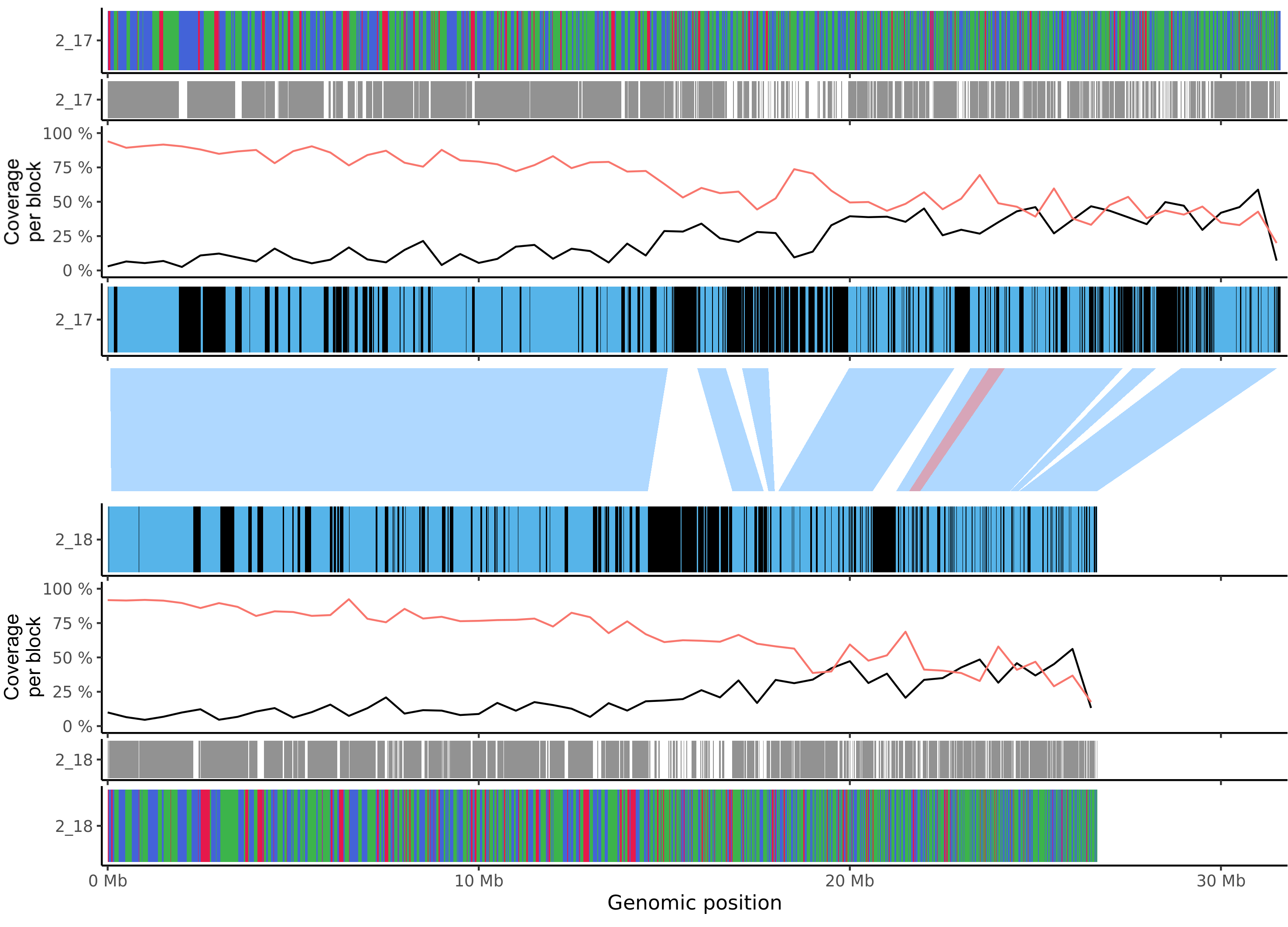

Supplement: Supplementary file 3 — Supplement S3 Supplementary Data. [file PBI-23-874-s002.zip › Supplementary_data/sequence_visualization/Apple/mdomestica_gala_chr_1.png]

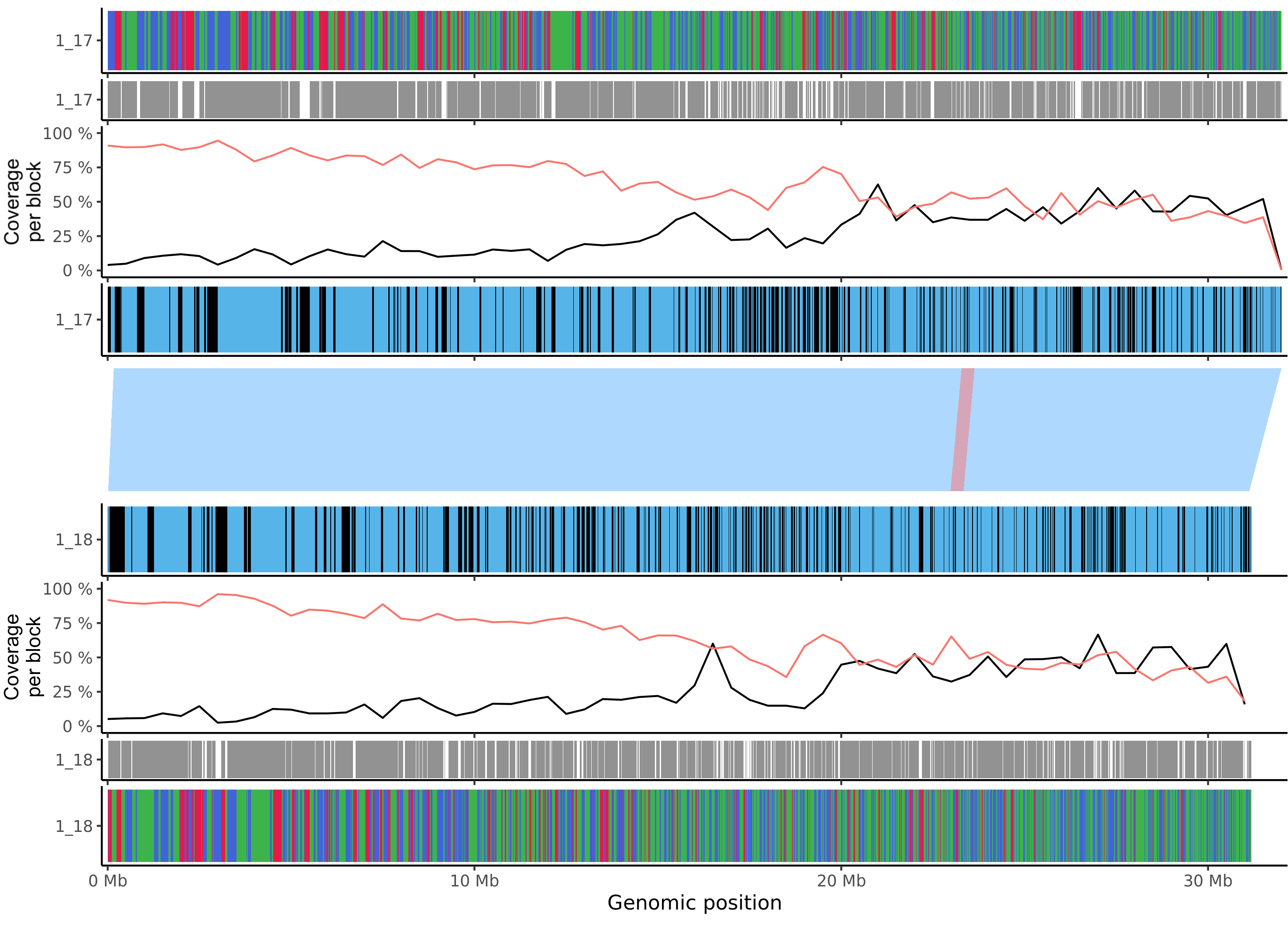

Supplement: Supplementary file 3 — Supplement S3 Supplementary Data. [file PBI-23-874-s002.zip › Supplementary_data/sequence_visualization/Apple/msylvestris_chr_1.png]

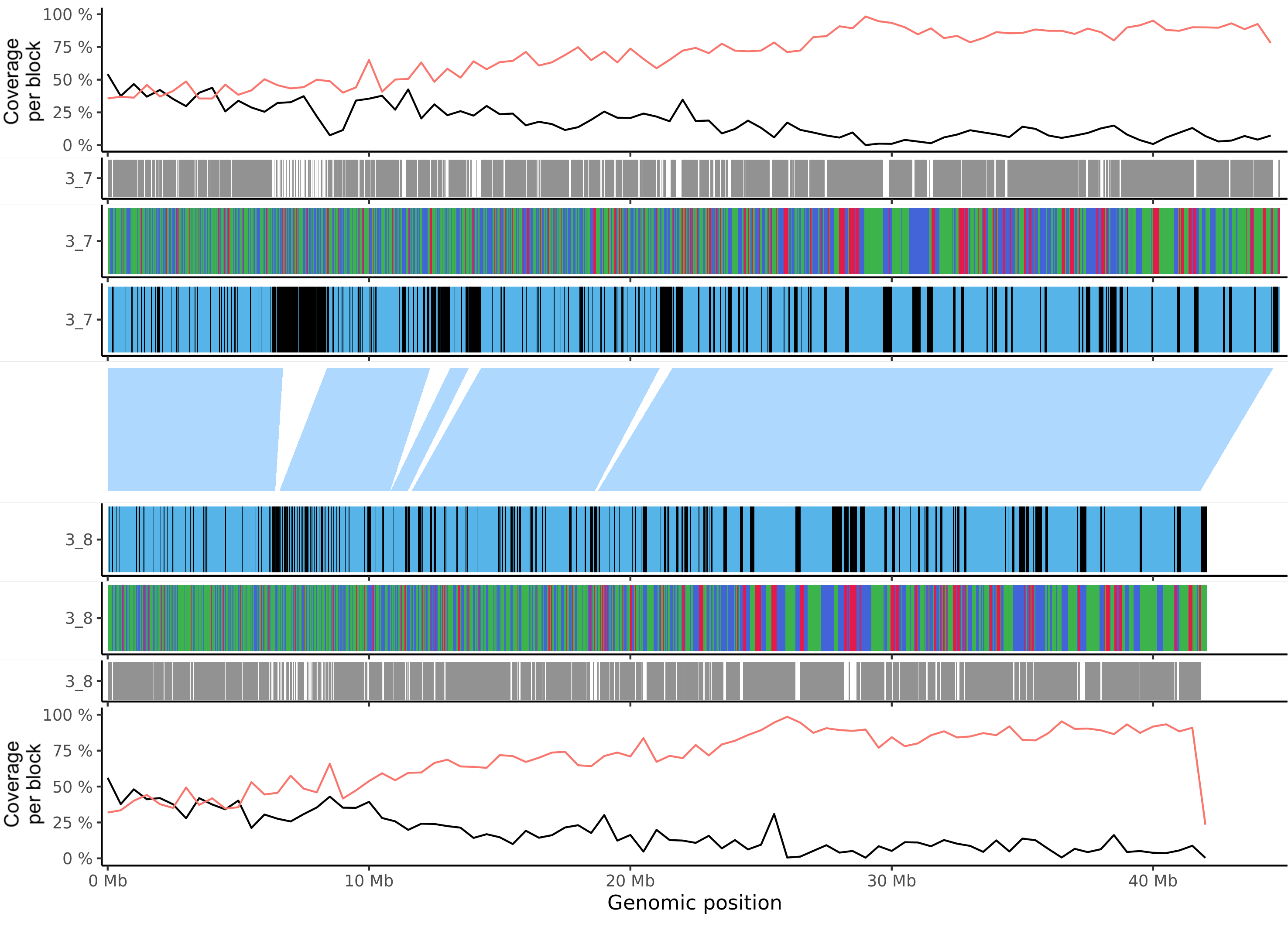

Supplement: Supplementary file 3 — Supplement S3 Supplementary Data. [file PBI-23-874-s002.zip › Supplementary_data/sequence_visualization/Apple/msieversii_chr_13.png]

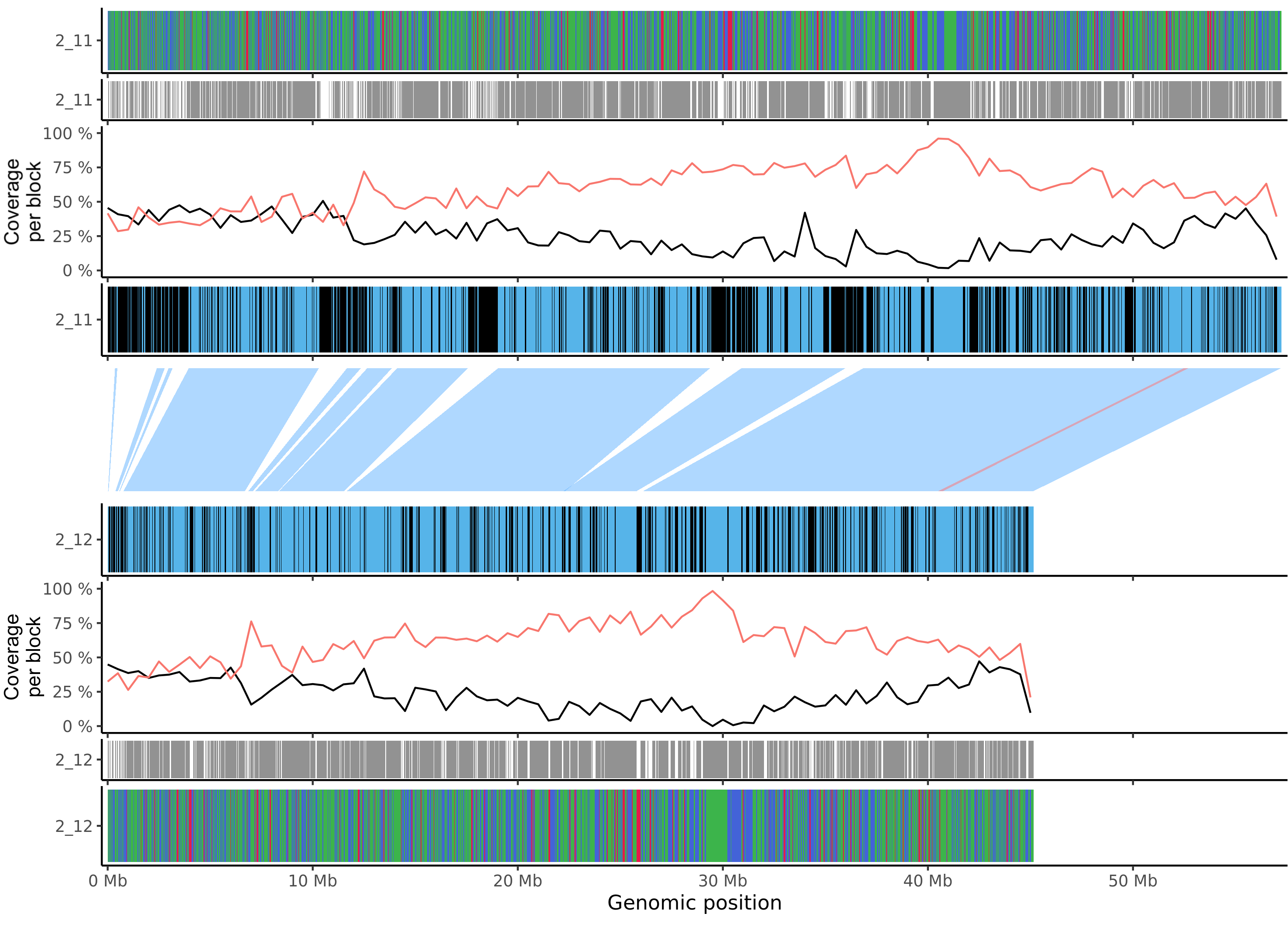

Supplement: Supplementary file 3 — Supplement S3 Supplementary Data. [file PBI-23-874-s002.zip › Supplementary_data/sequence_visualization/Apple/mdomestica_gala_chr_15.png]

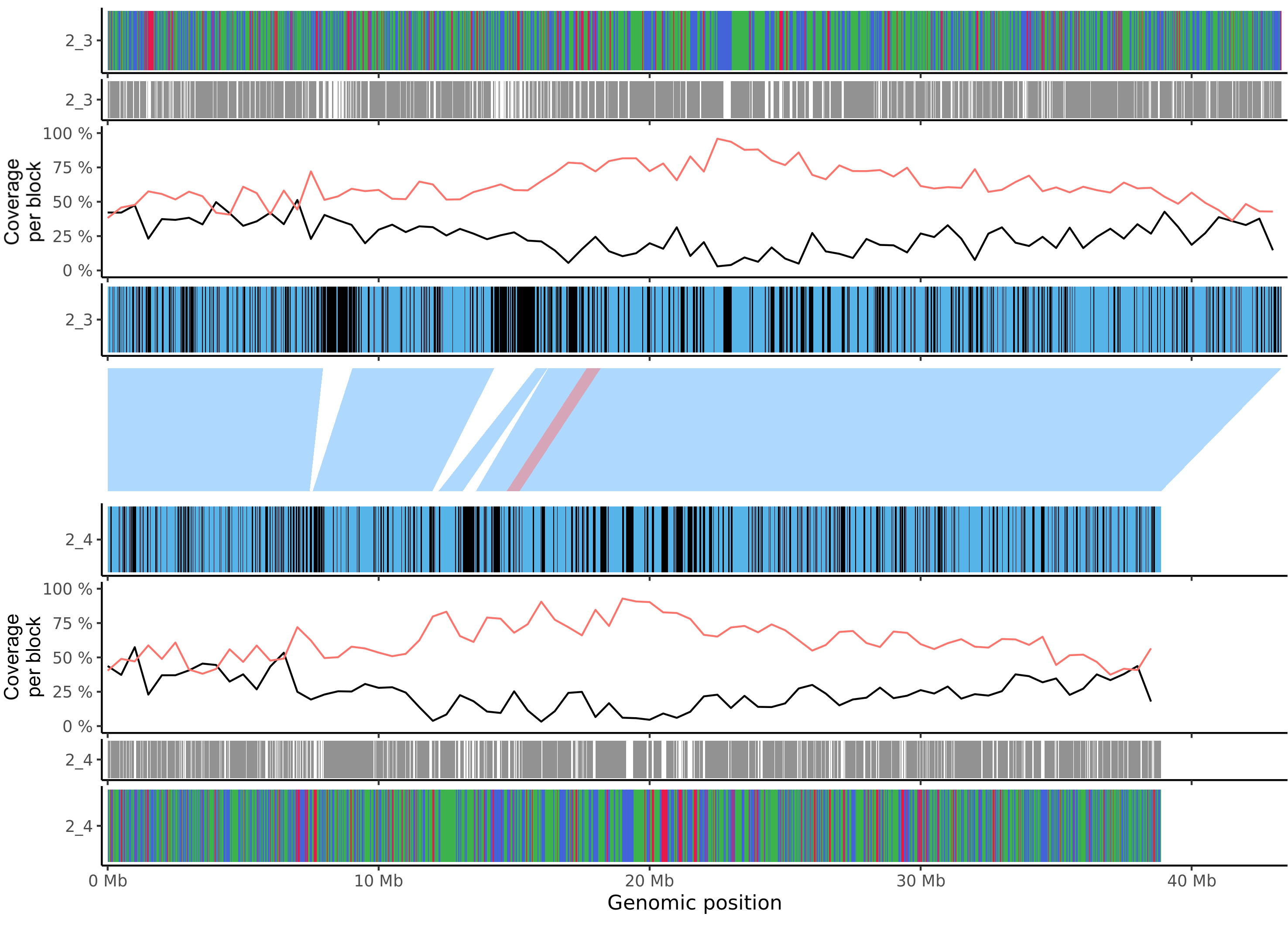

Supplement: Supplementary file 3 — Supplement S3 Supplementary Data. [file PBI-23-874-s002.zip › Supplementary_data/sequence_visualization/Apple/mdomestica_gala_chr_11.png]

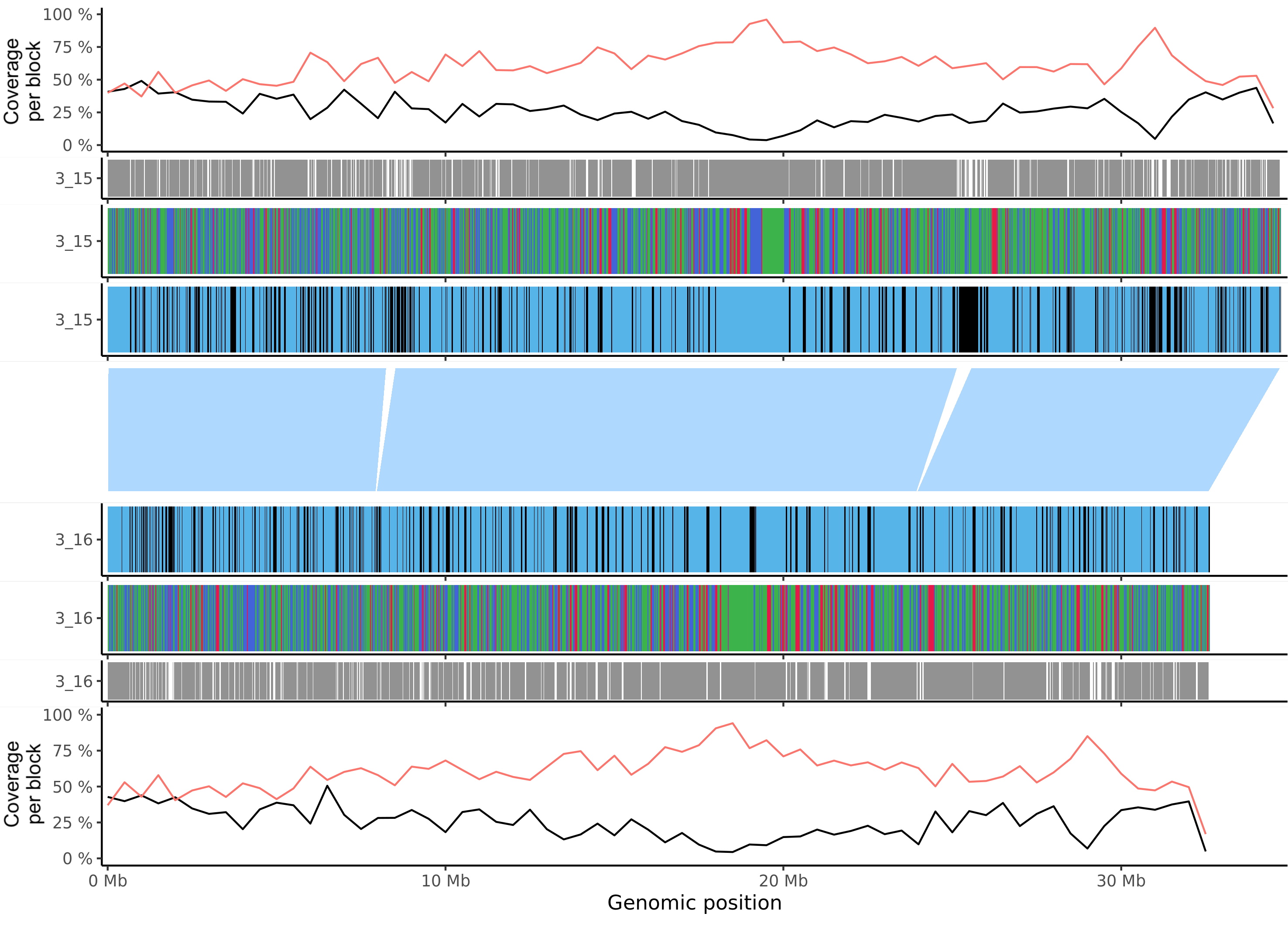

Supplement: Supplementary file 3 — Supplement S3 Supplementary Data. [file PBI-23-874-s002.zip › Supplementary_data/sequence_visualization/Apple/msieversii_chr_17.png]

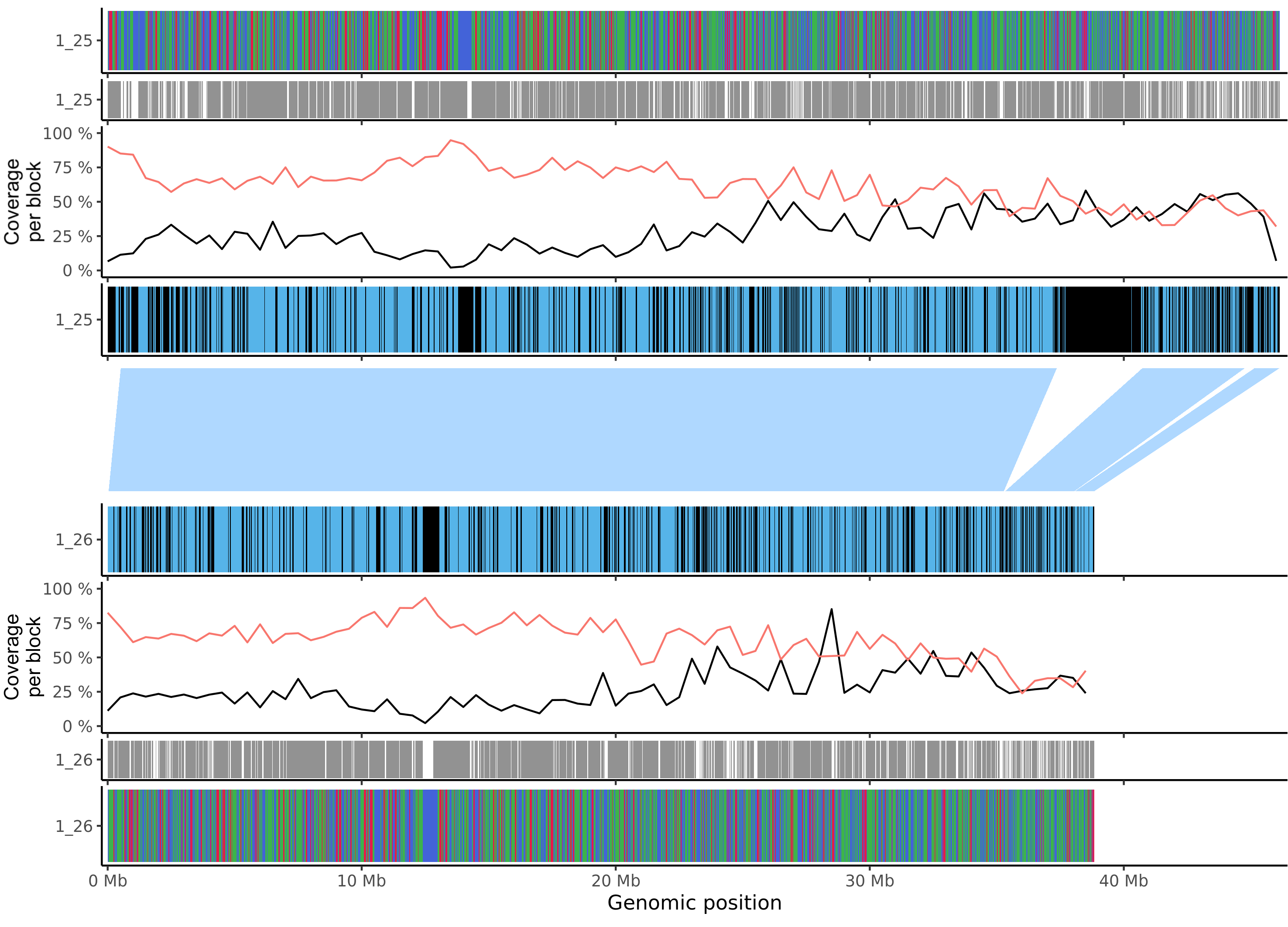

Supplement: Supplementary file 3 — Supplement S3 Supplementary Data. [file PBI-23-874-s002.zip › Supplementary_data/sequence_visualization/Apple/msylvestris_chr_5.png]

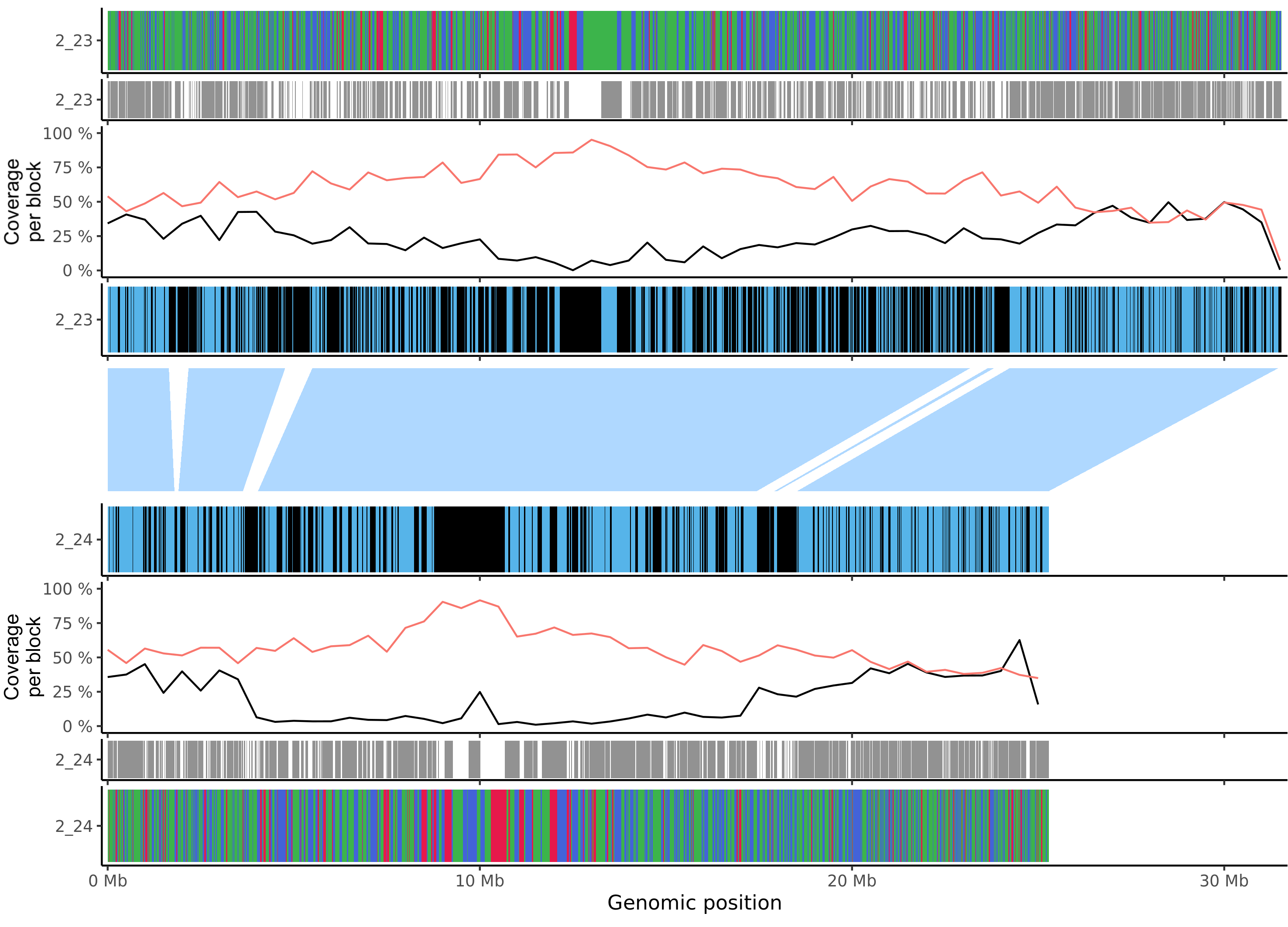

Supplement: Supplementary file 3 — Supplement S3 Supplementary Data. [file PBI-23-874-s002.zip › Supplementary_data/sequence_visualization/Apple/mdomestica_gala_chr_4.png]

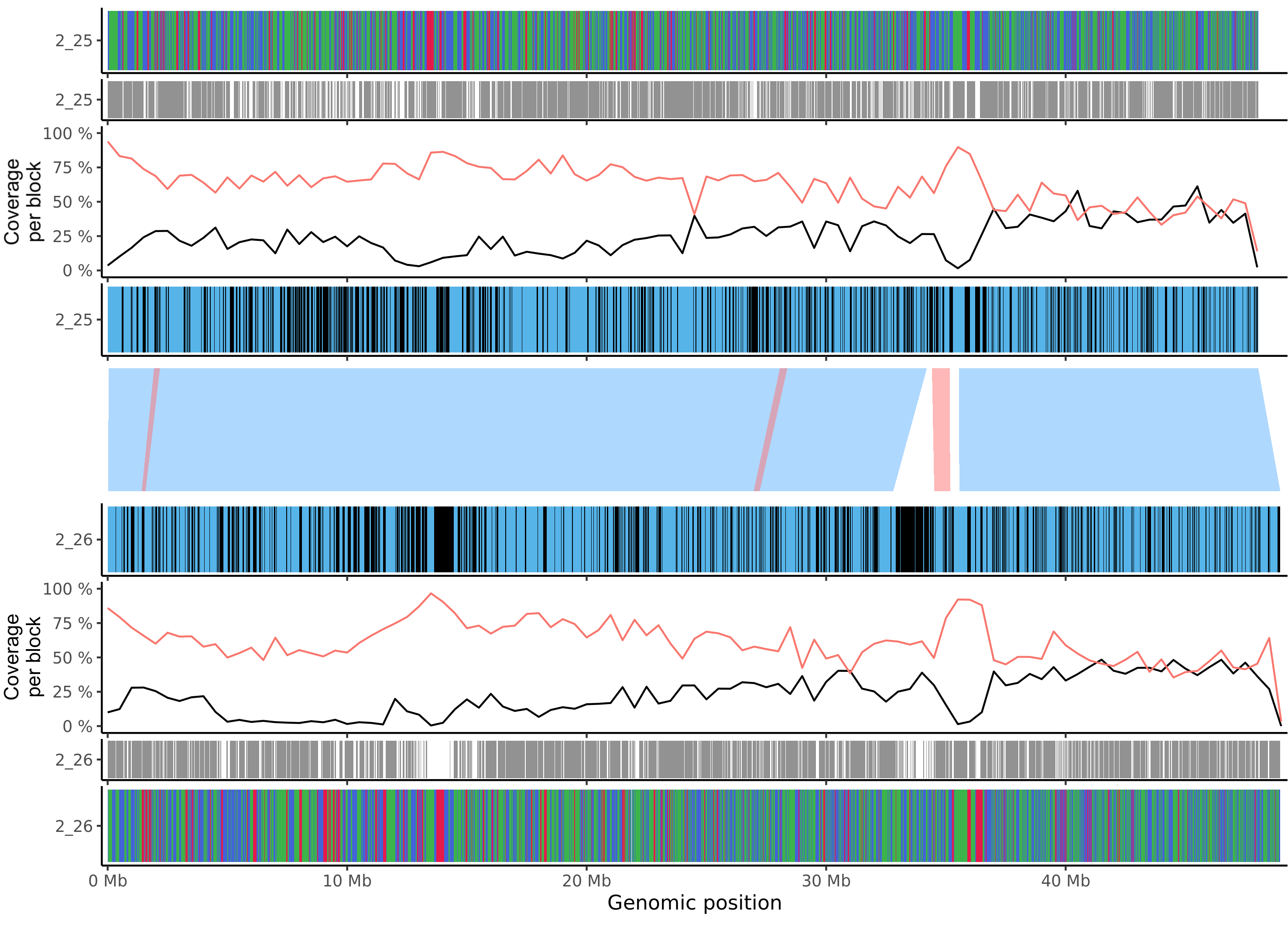

Supplement: Supplementary file 3 — Supplement S3 Supplementary Data. [file PBI-23-874-s002.zip › Supplementary_data/sequence_visualization/Apple/mdomestica_gala_chr_5.png]

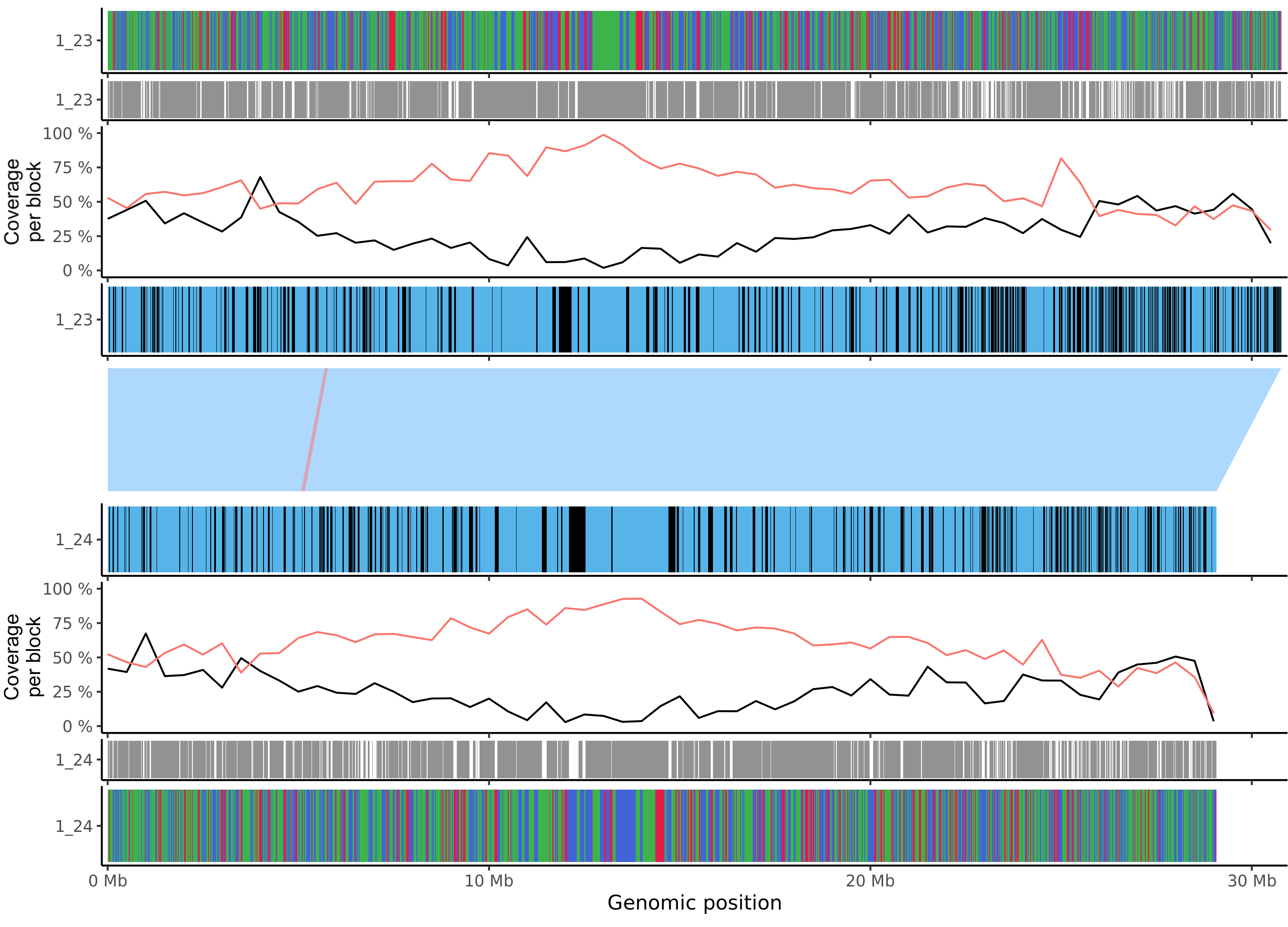

Supplement: Supplementary file 3 — Supplement S3 Supplementary Data. [file PBI-23-874-s002.zip › Supplementary_data/sequence_visualization/Apple/msylvestris_chr_4.png]

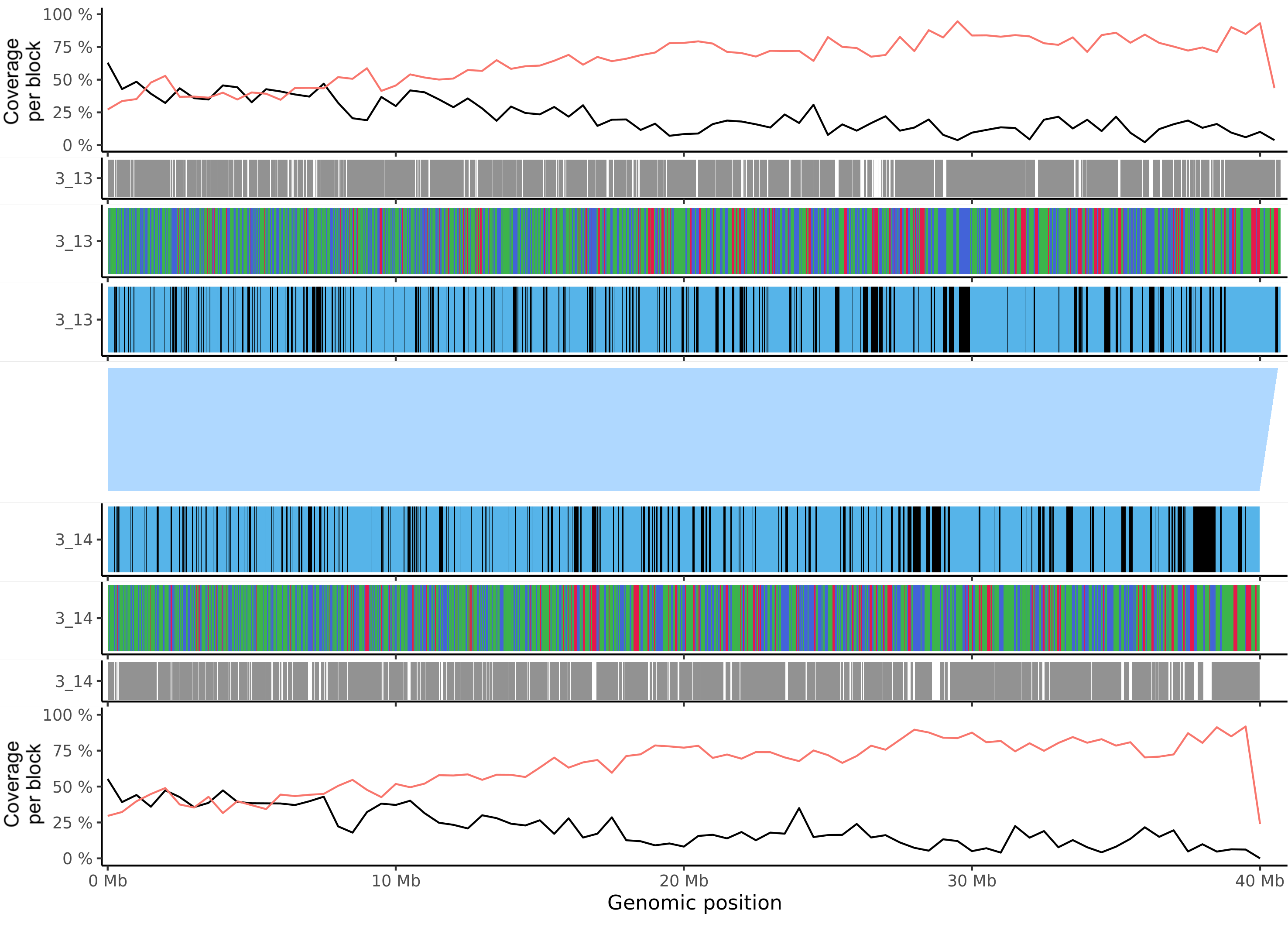

Supplement: Supplementary file 3 — Supplement S3 Supplementary Data. [file PBI-23-874-s002.zip › Supplementary_data/sequence_visualization/Apple/msieversii_chr_16.png]

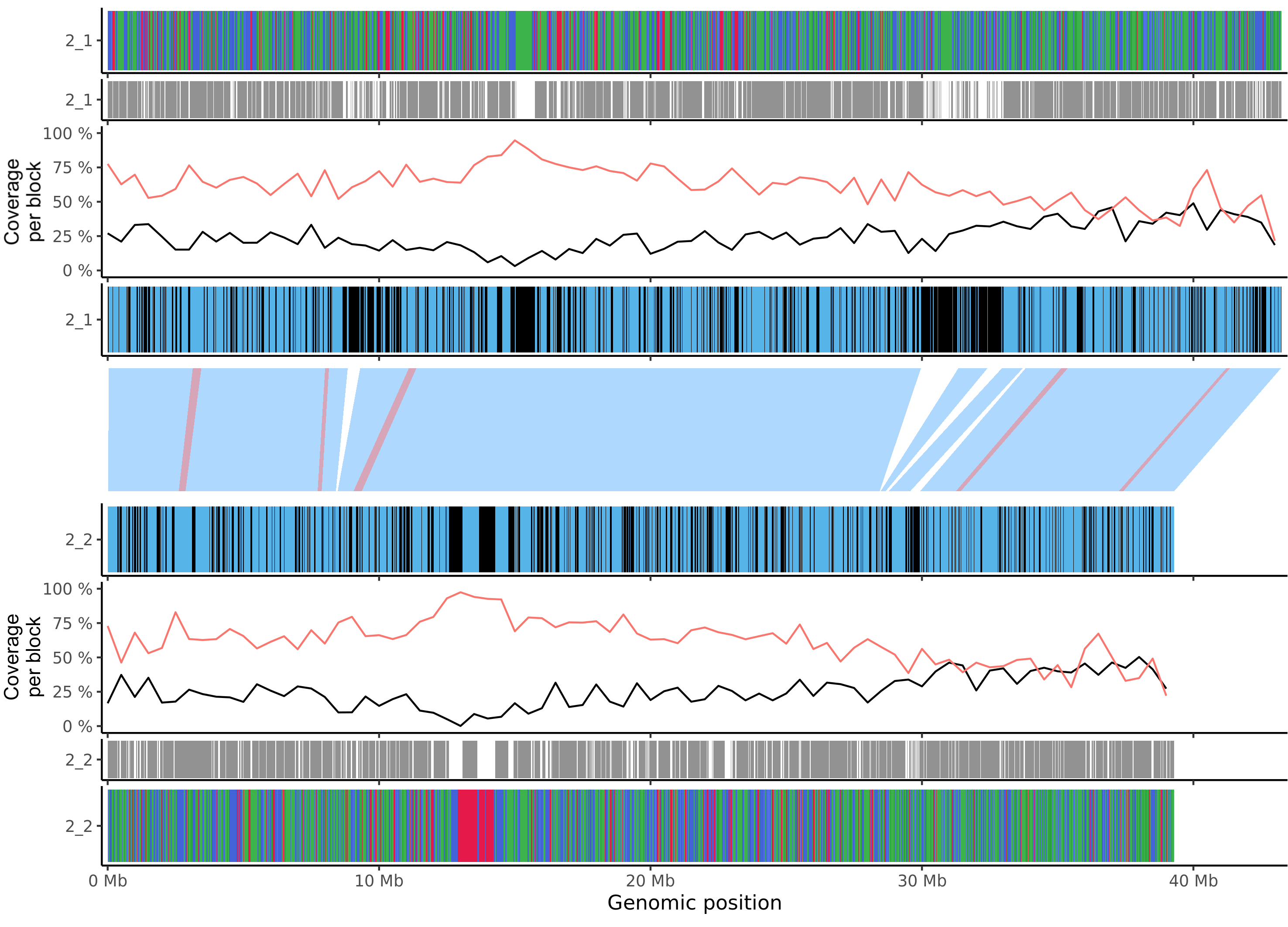

Supplement: Supplementary file 3 — Supplement S3 Supplementary Data. [file PBI-23-874-s002.zip › Supplementary_data/sequence_visualization/Apple/mdomestica_gala_chr_10.png]

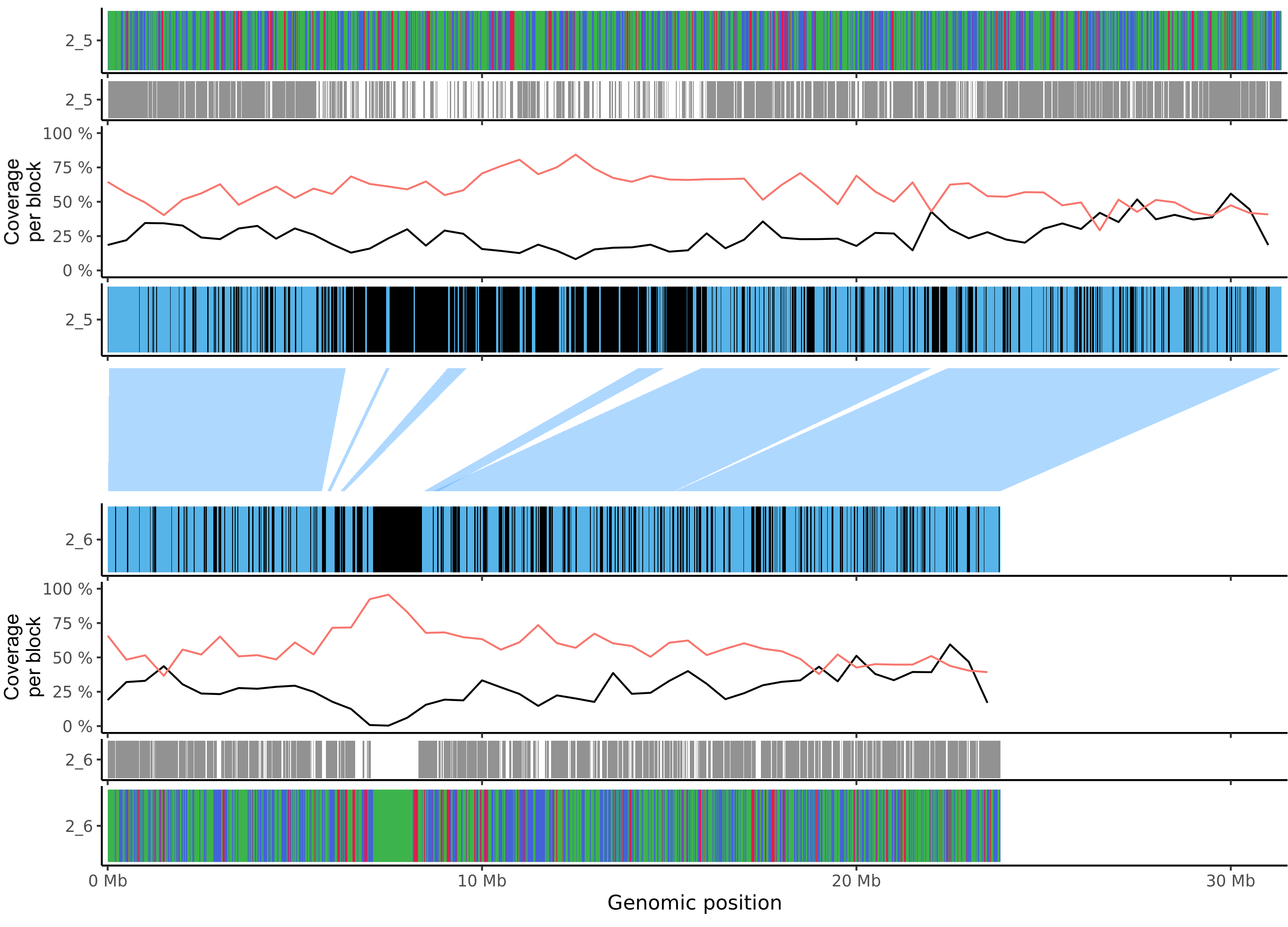

Supplement: Supplementary file 3 — Supplement S3 Supplementary Data. [file PBI-23-874-s002.zip › Supplementary_data/sequence_visualization/Apple/mdomestica_gala_chr_12.png]

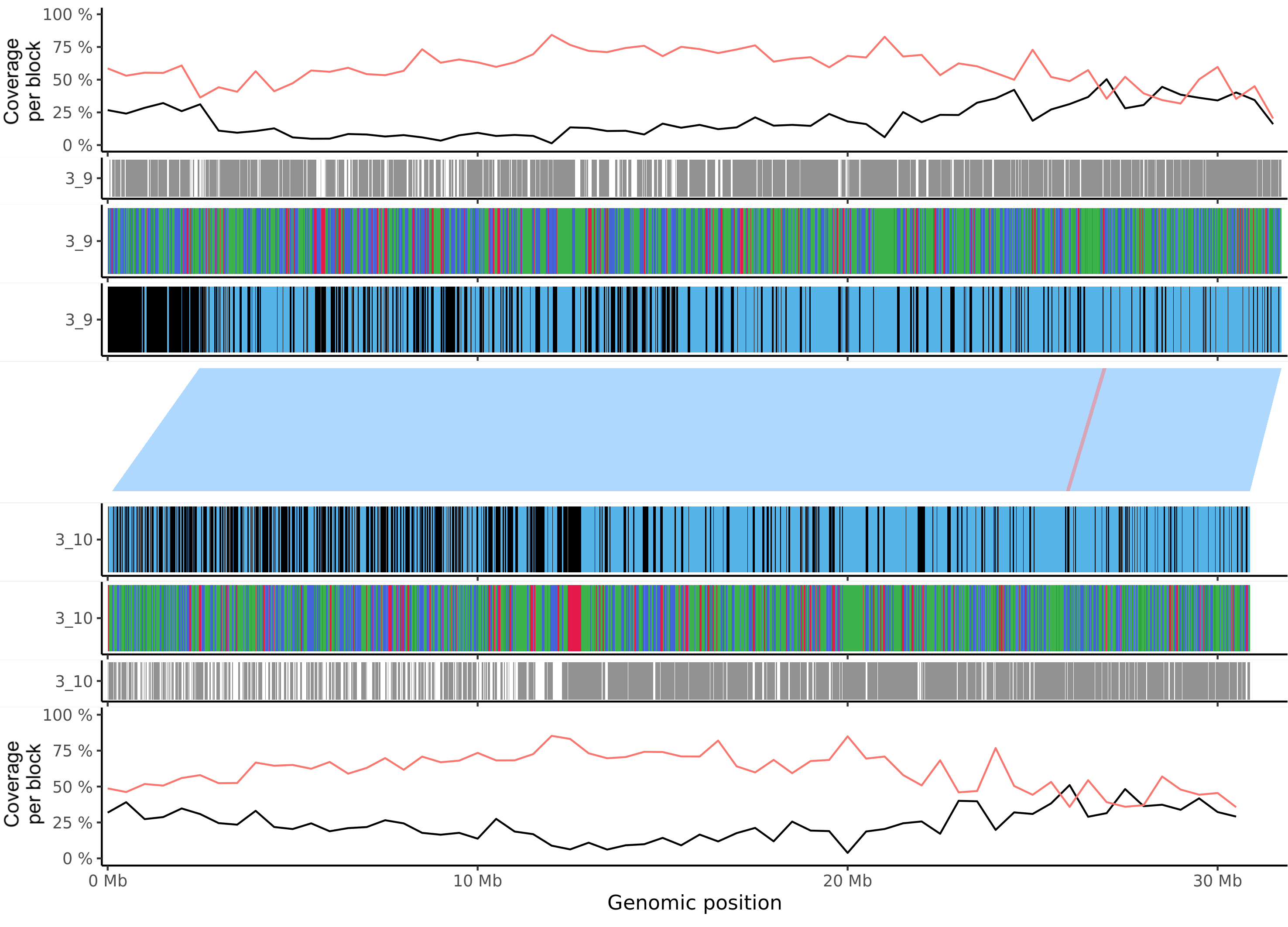

Supplement: Supplementary file 3 — Supplement S3 Supplementary Data. [file PBI-23-874-s002.zip › Supplementary_data/sequence_visualization/Apple/msieversii_chr_14.png]

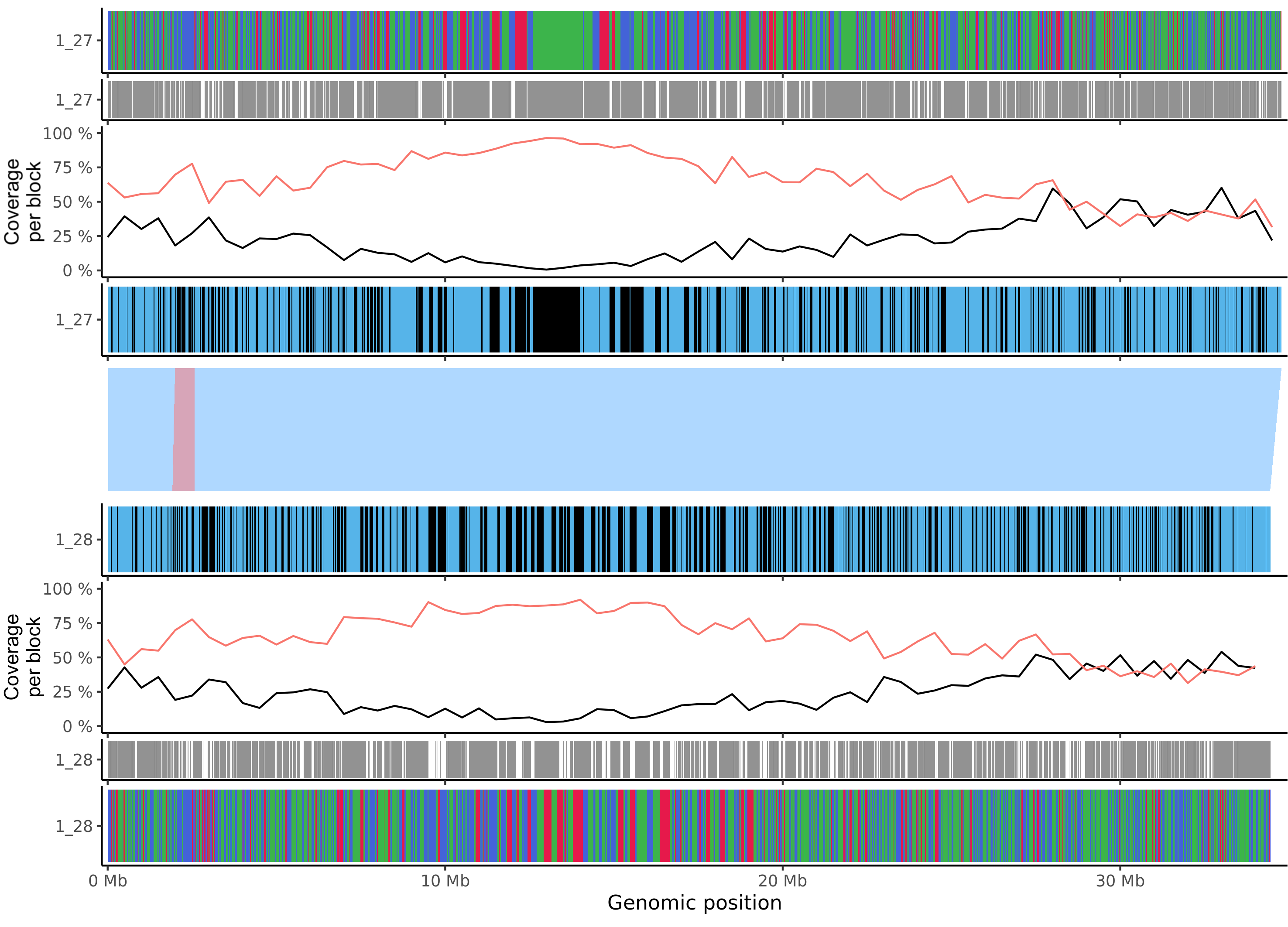

Supplement: Supplementary file 3 — Supplement S3 Supplementary Data. [file PBI-23-874-s002.zip › Supplementary_data/sequence_visualization/Apple/msylvestris_chr_6.png]

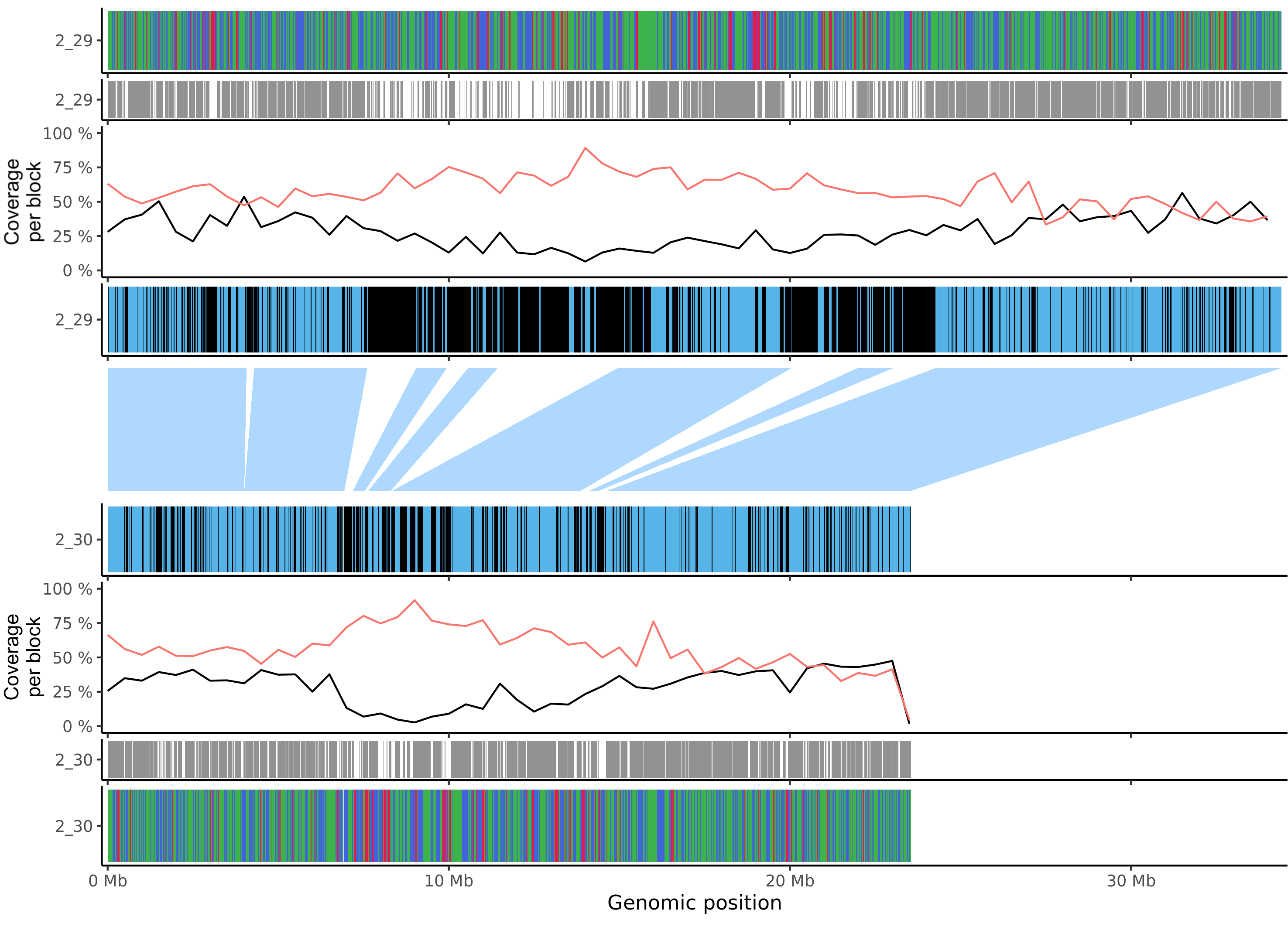

Supplement: Supplementary file 3 — Supplement S3 Supplementary Data. [file PBI-23-874-s002.zip › Supplementary_data/sequence_visualization/Apple/mdomestica_gala_chr_7.png]

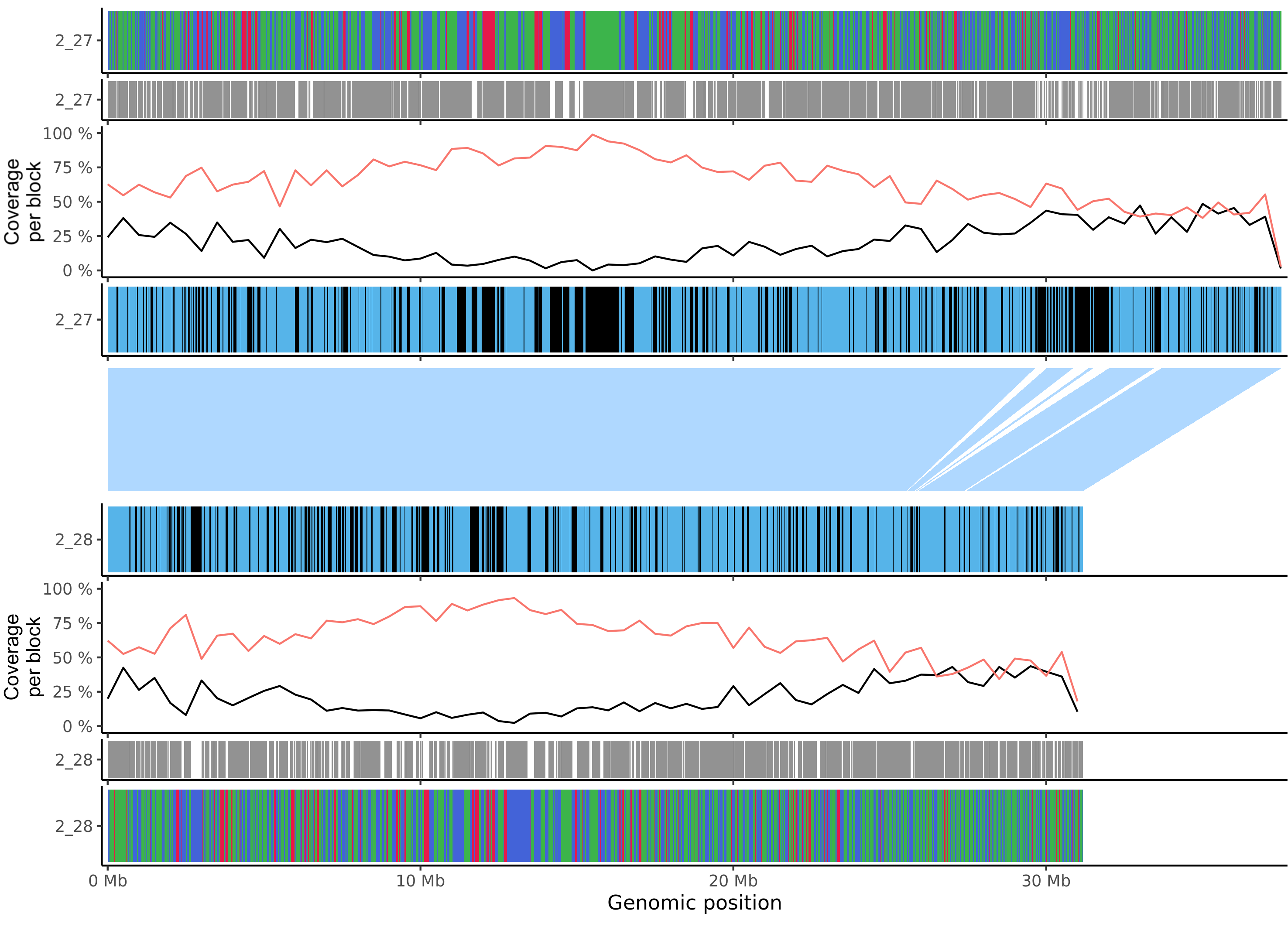

Supplement: Supplementary file 3 — Supplement S3 Supplementary Data. [file PBI-23-874-s002.zip › Supplementary_data/sequence_visualization/Apple/mdomestica_gala_chr_6.png]

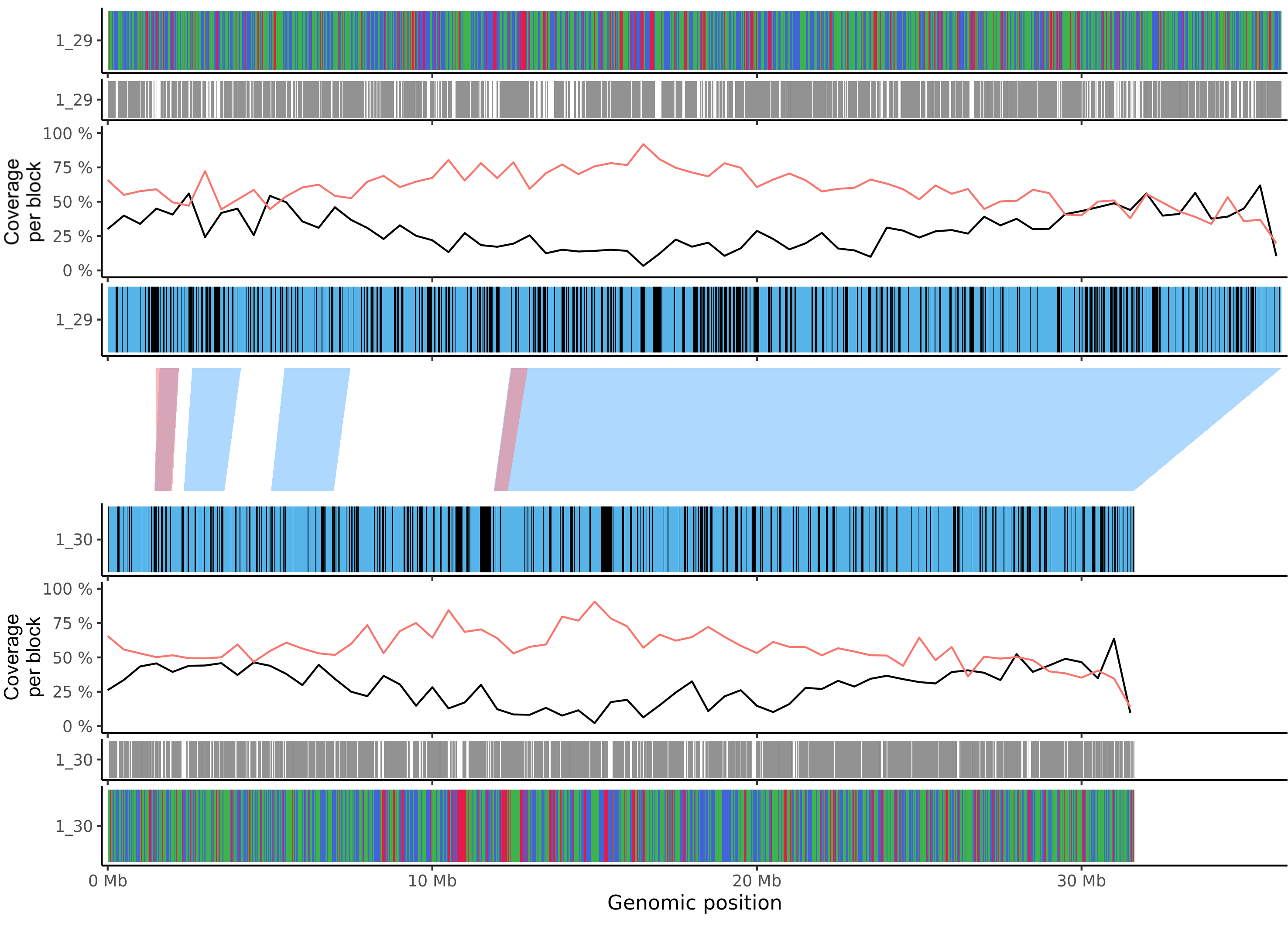

Supplement: Supplementary file 3 — Supplement S3 Supplementary Data. [file PBI-23-874-s002.zip › Supplementary_data/sequence_visualization/Apple/msylvestris_chr_7.png]

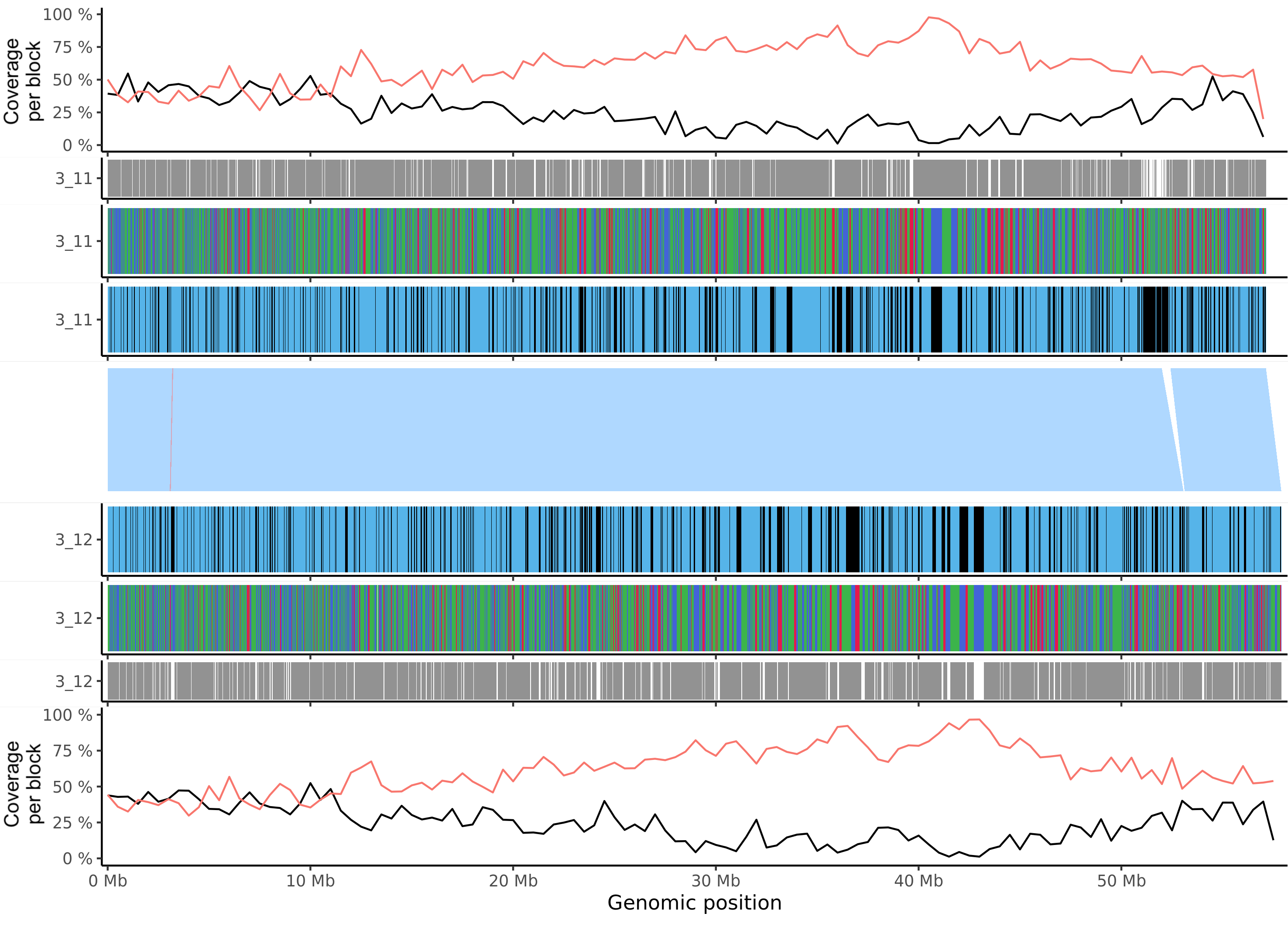

Supplement: Supplementary file 3 — Supplement S3 Supplementary Data. [file PBI-23-874-s002.zip › Supplementary_data/sequence_visualization/Apple/msieversii_chr_15.png]

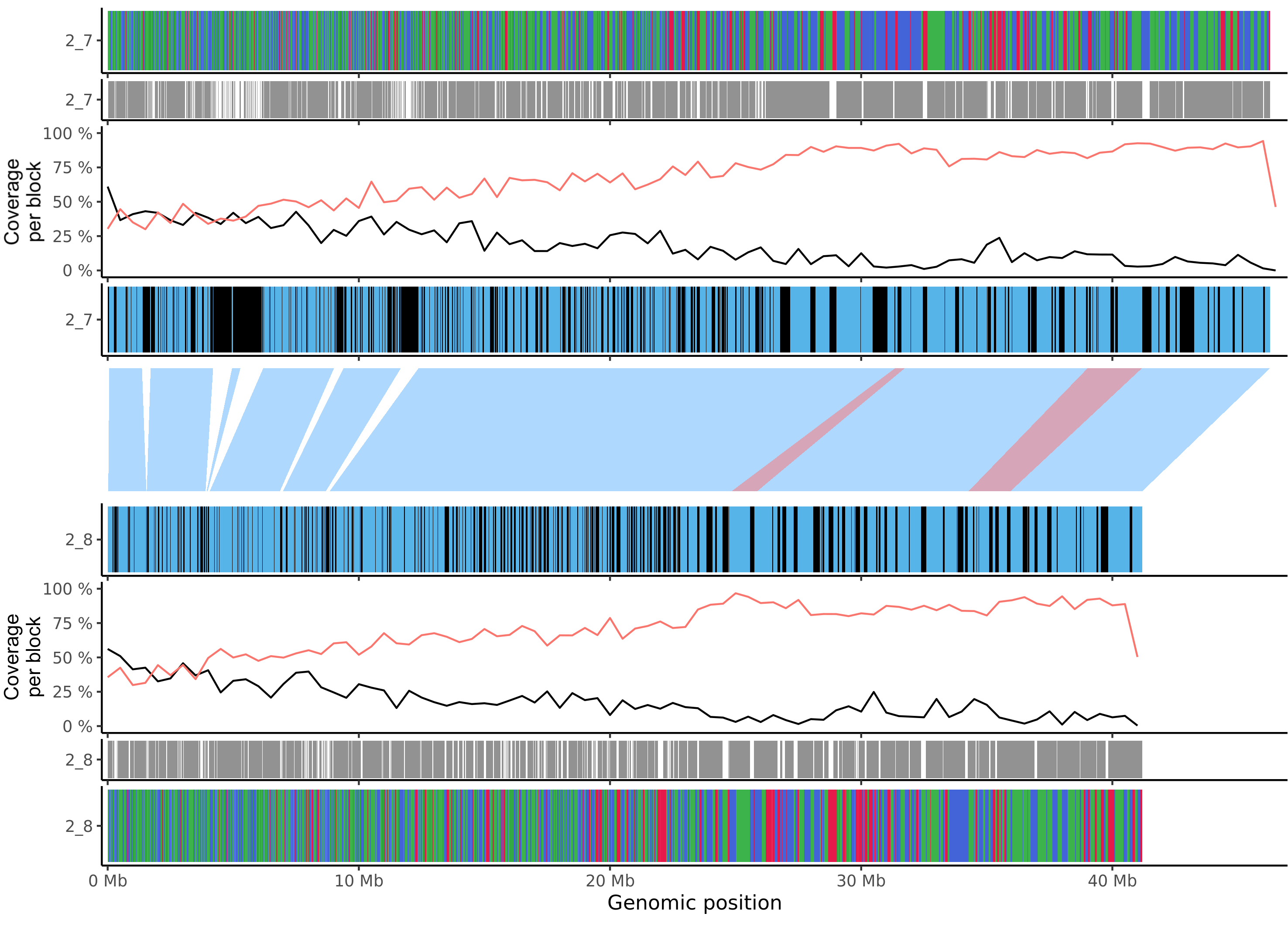

Supplement: Supplementary file 3 — Supplement S3 Supplementary Data. [file PBI-23-874-s002.zip › Supplementary_data/sequence_visualization/Apple/mdomestica_gala_chr_13.png]

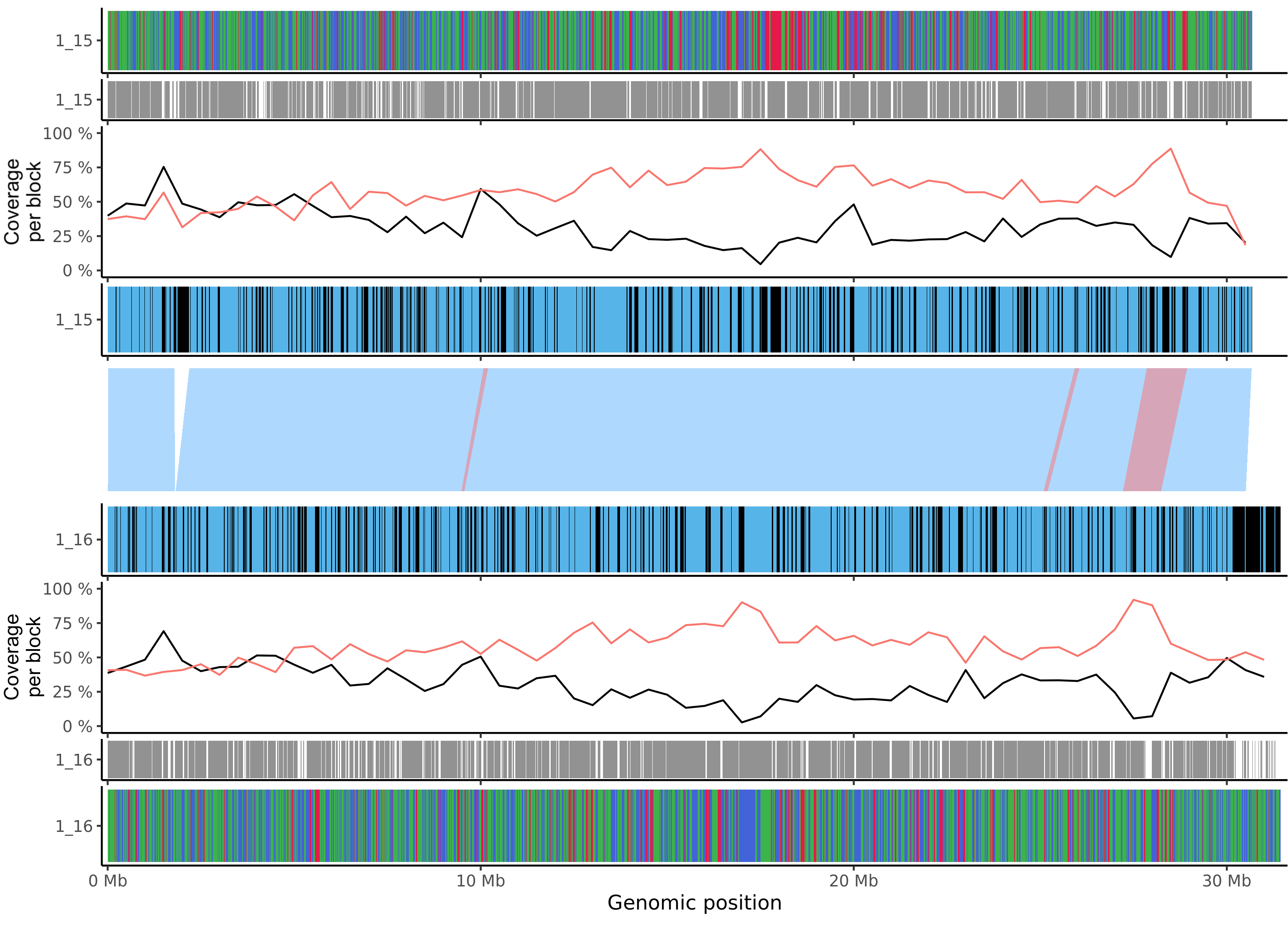

Supplement: Supplementary file 3 — Supplement S3 Supplementary Data. [file PBI-23-874-s002.zip › Supplementary_data/sequence_visualization/Apple/msylvestris_chr_17.png]

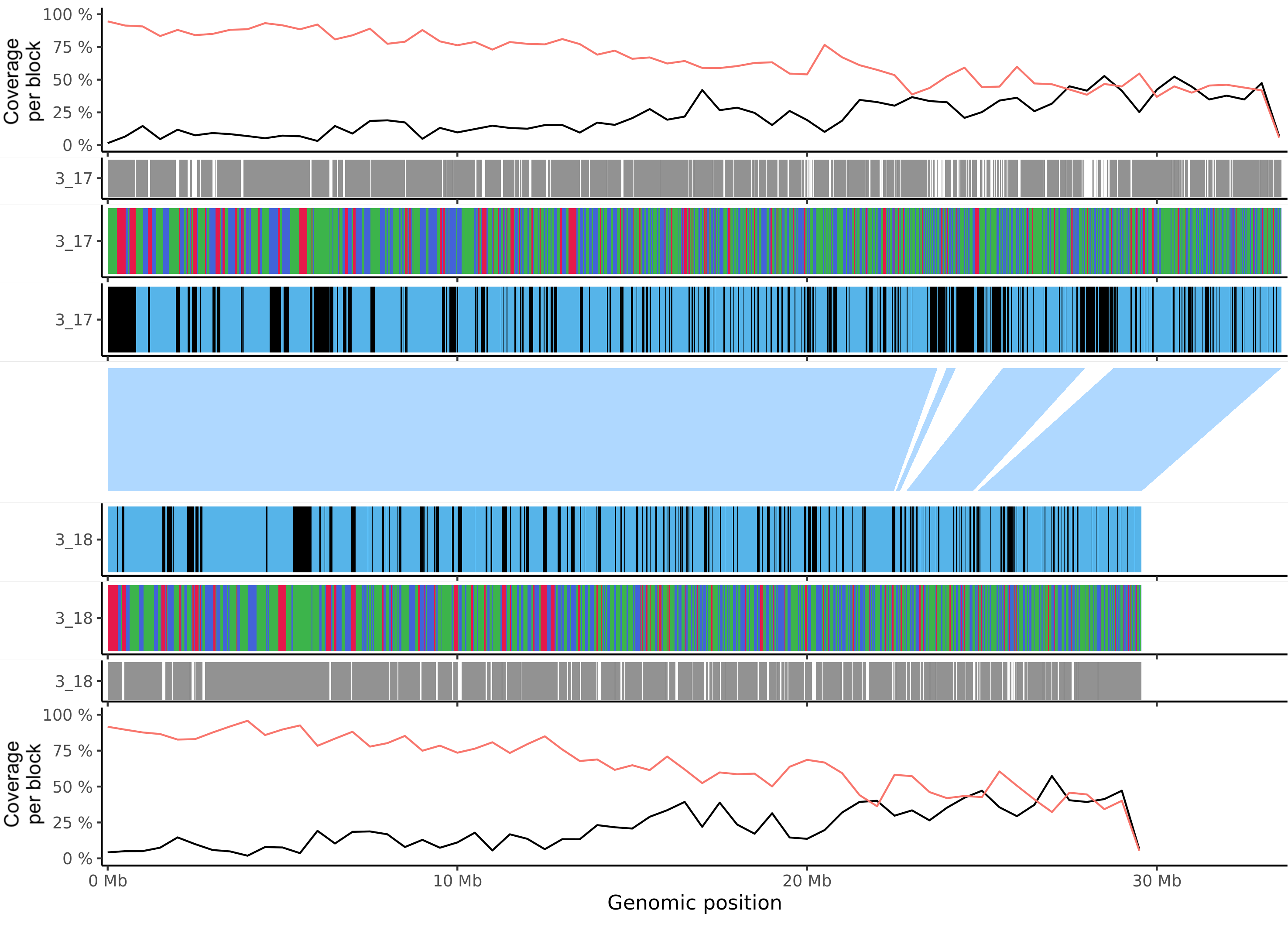

Supplement: Supplementary file 3 — Supplement S3 Supplementary Data. [file PBI-23-874-s002.zip › Supplementary_data/sequence_visualization/Apple/msieversii_chr_1.png]

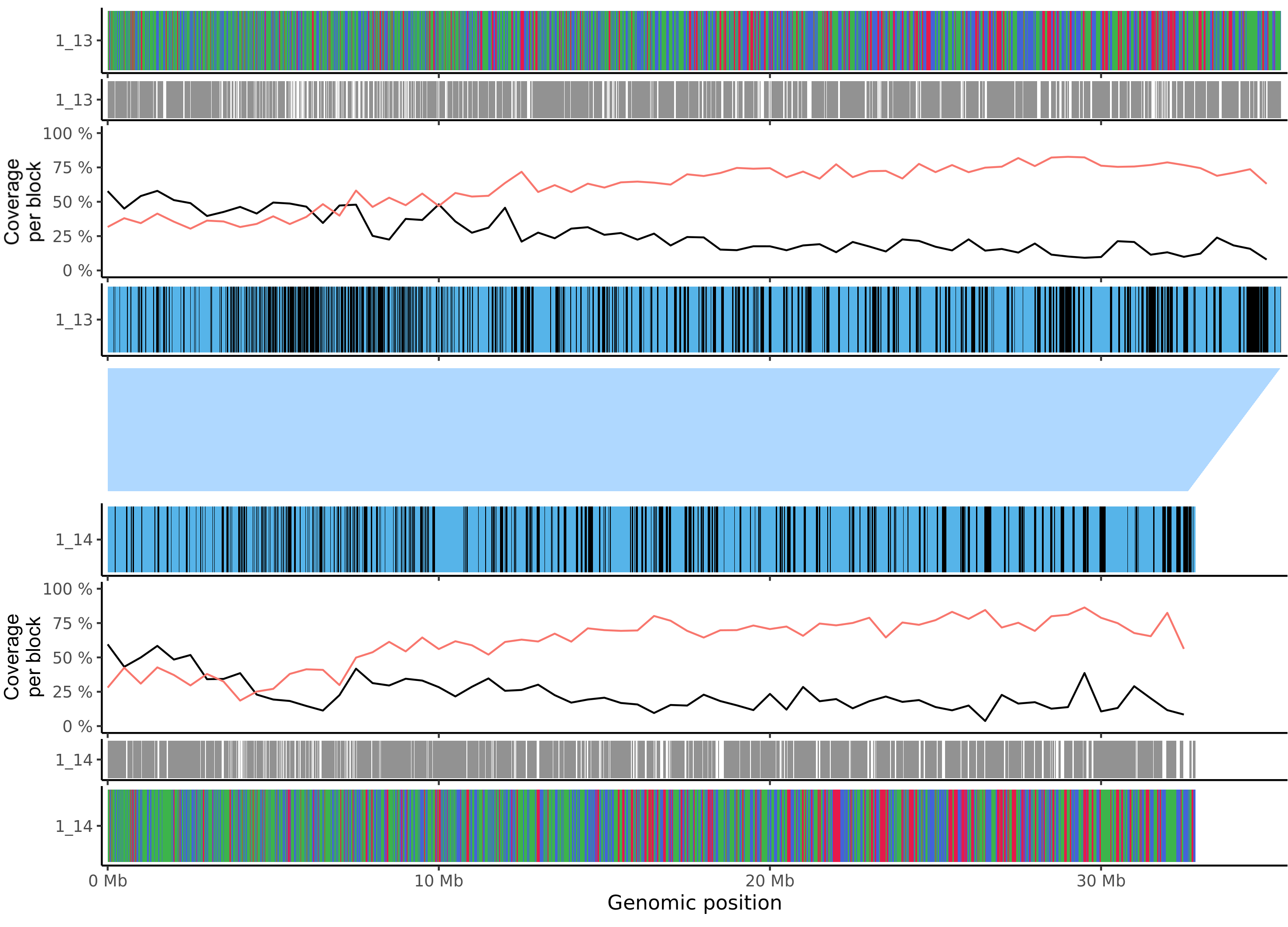

Supplement: Supplementary file 3 — Supplement S3 Supplementary Data. [file PBI-23-874-s002.zip › Supplementary_data/sequence_visualization/Apple/msylvestris_chr_16.png]

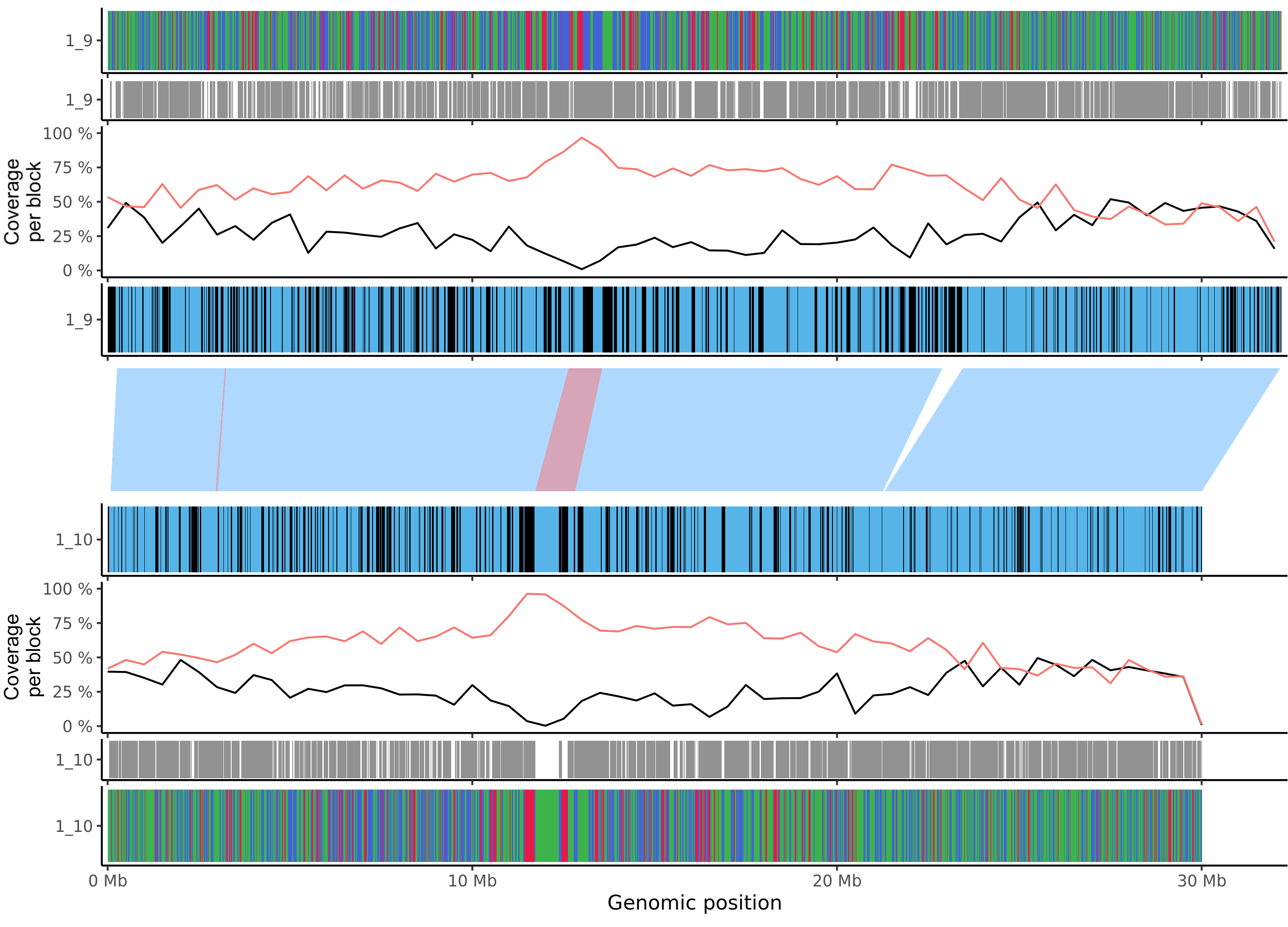

Supplement: Supplementary file 3 — Supplement S3 Supplementary Data. [file PBI-23-874-s002.zip › Supplementary_data/sequence_visualization/Apple/msylvestris_chr_14.png]

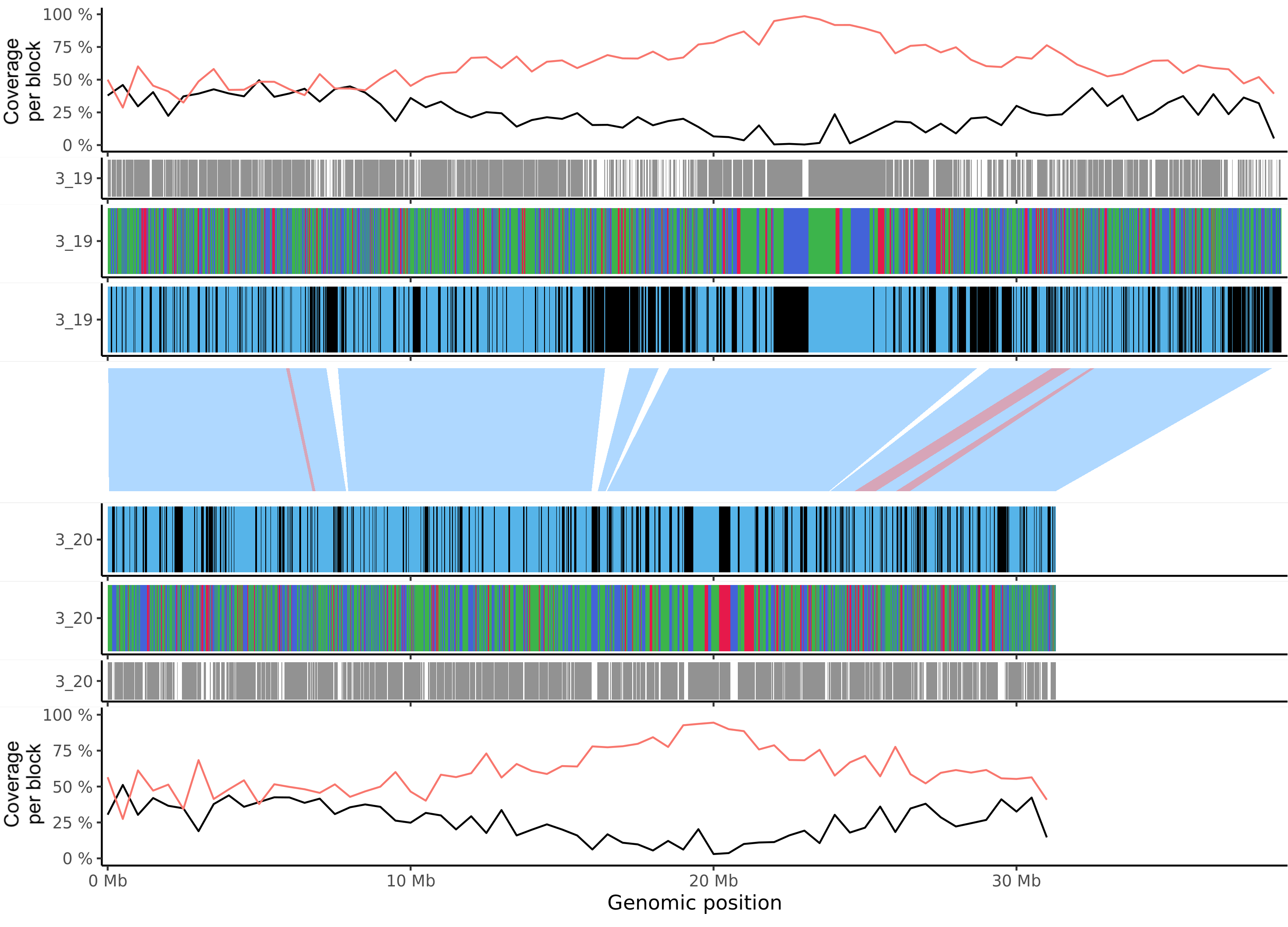

Supplement: Supplementary file 3 — Supplement S3 Supplementary Data. [file PBI-23-874-s002.zip › Supplementary_data/sequence_visualization/Apple/msieversii_chr_2.png]

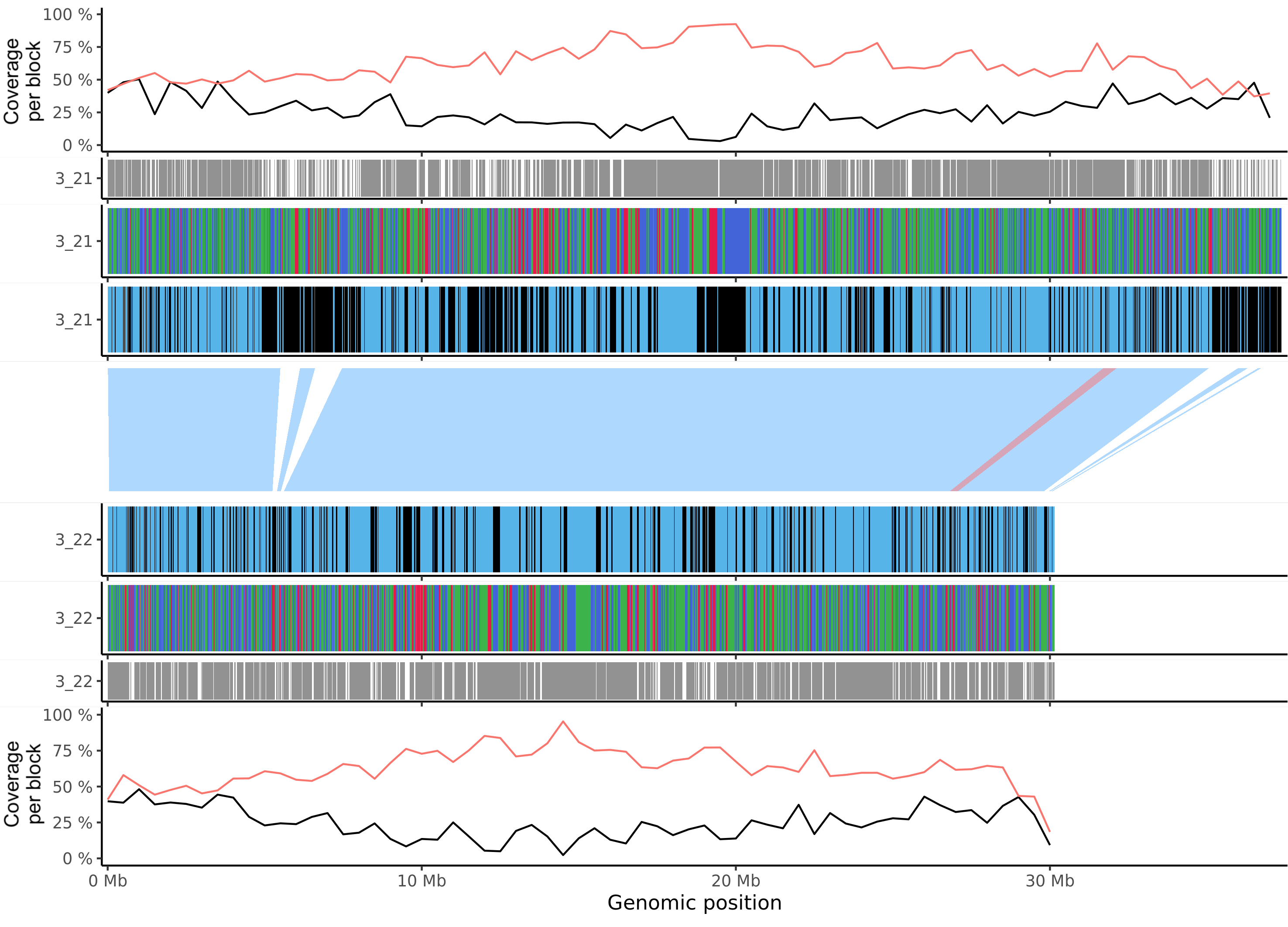

Supplement: Supplementary file 3 — Supplement S3 Supplementary Data. [file PBI-23-874-s002.zip › Supplementary_data/sequence_visualization/Apple/msieversii_chr_3.png]

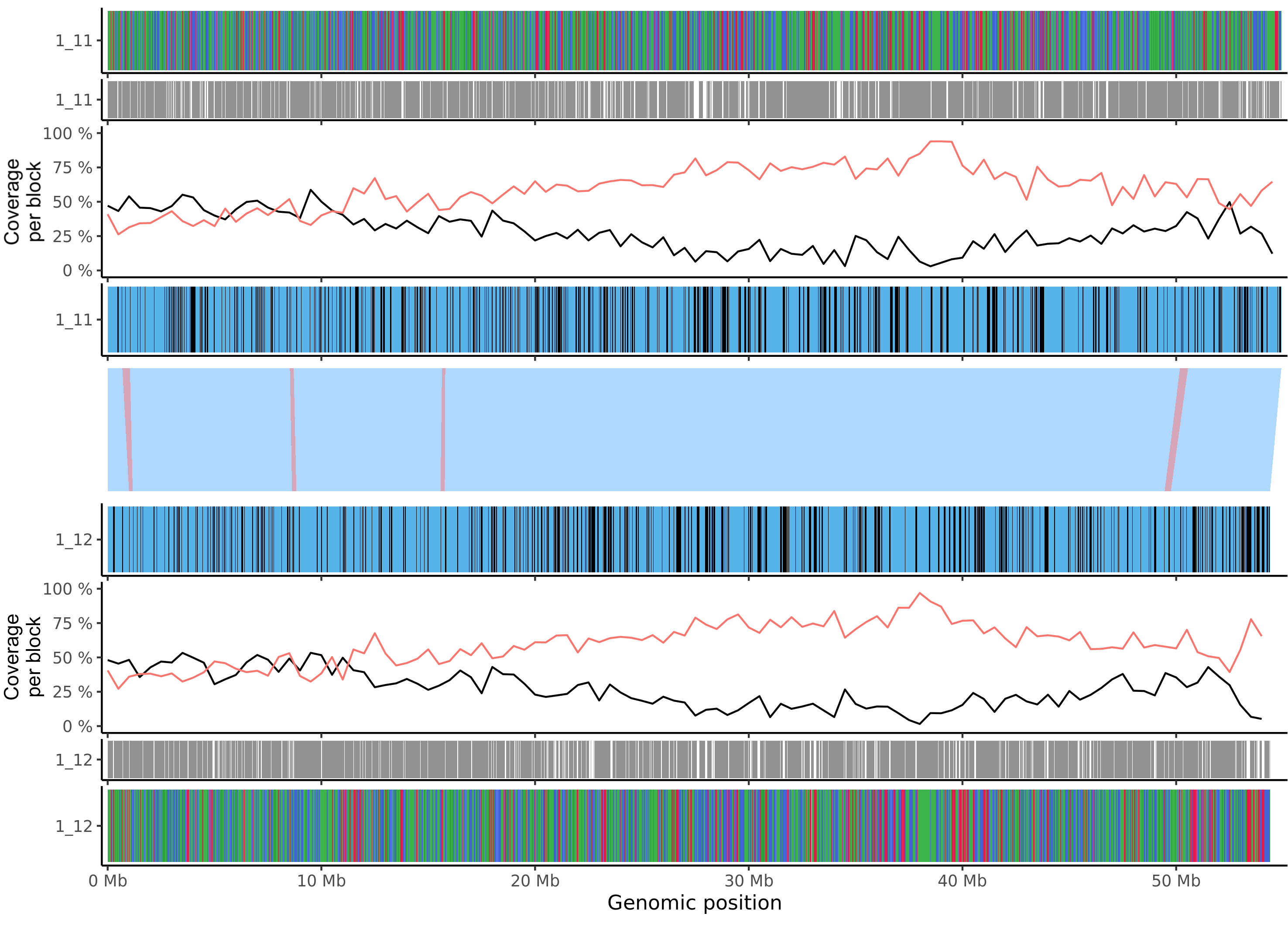

Supplement: Supplementary file 3 — Supplement S3 Supplementary Data. [file PBI-23-874-s002.zip › Supplementary_data/sequence_visualization/Apple/msylvestris_chr_15.png]

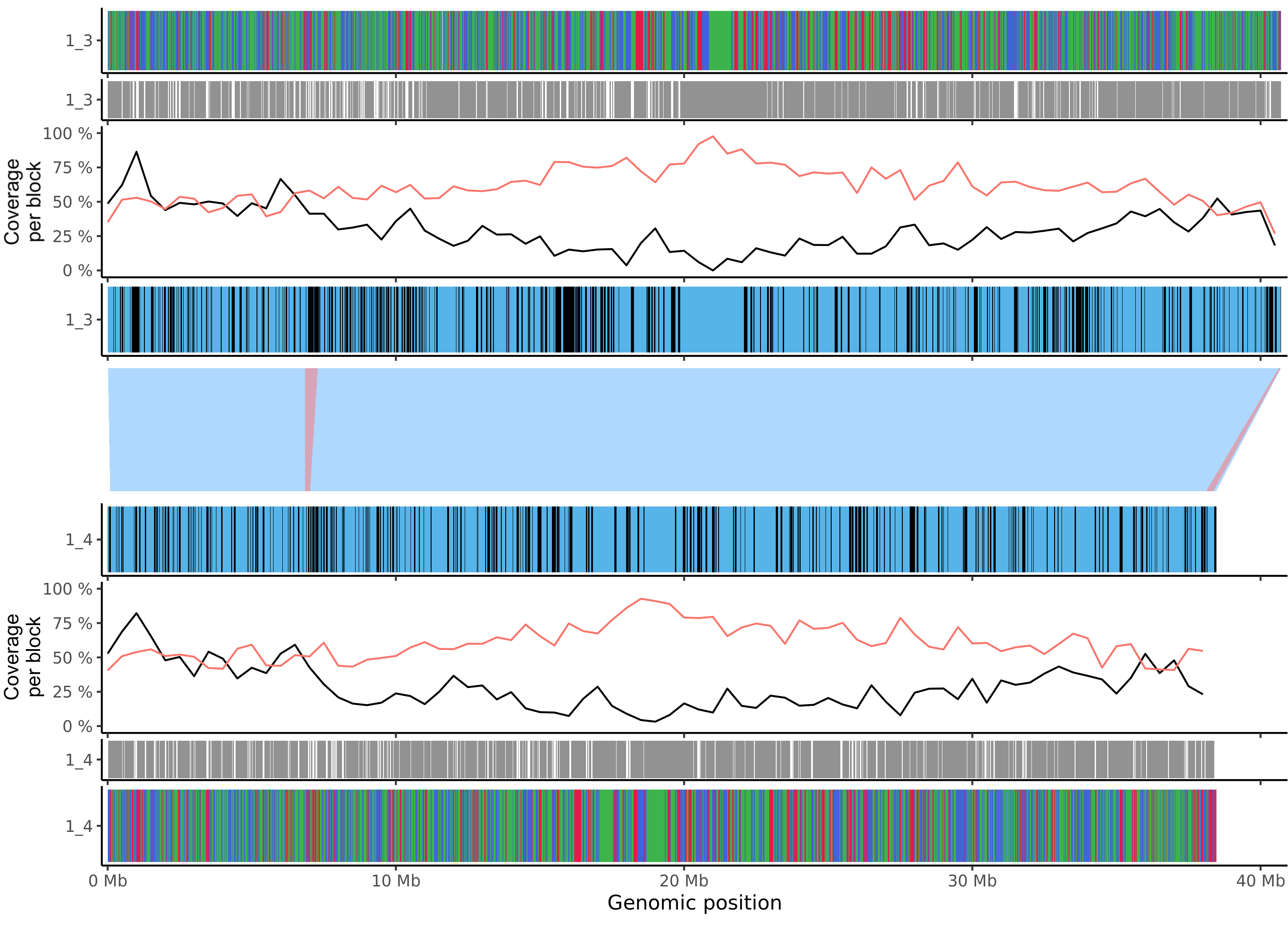

Supplement: Supplementary file 3 — Supplement S3 Supplementary Data. [file PBI-23-874-s002.zip › Supplementary_data/sequence_visualization/Apple/msylvestris_chr_11.png]

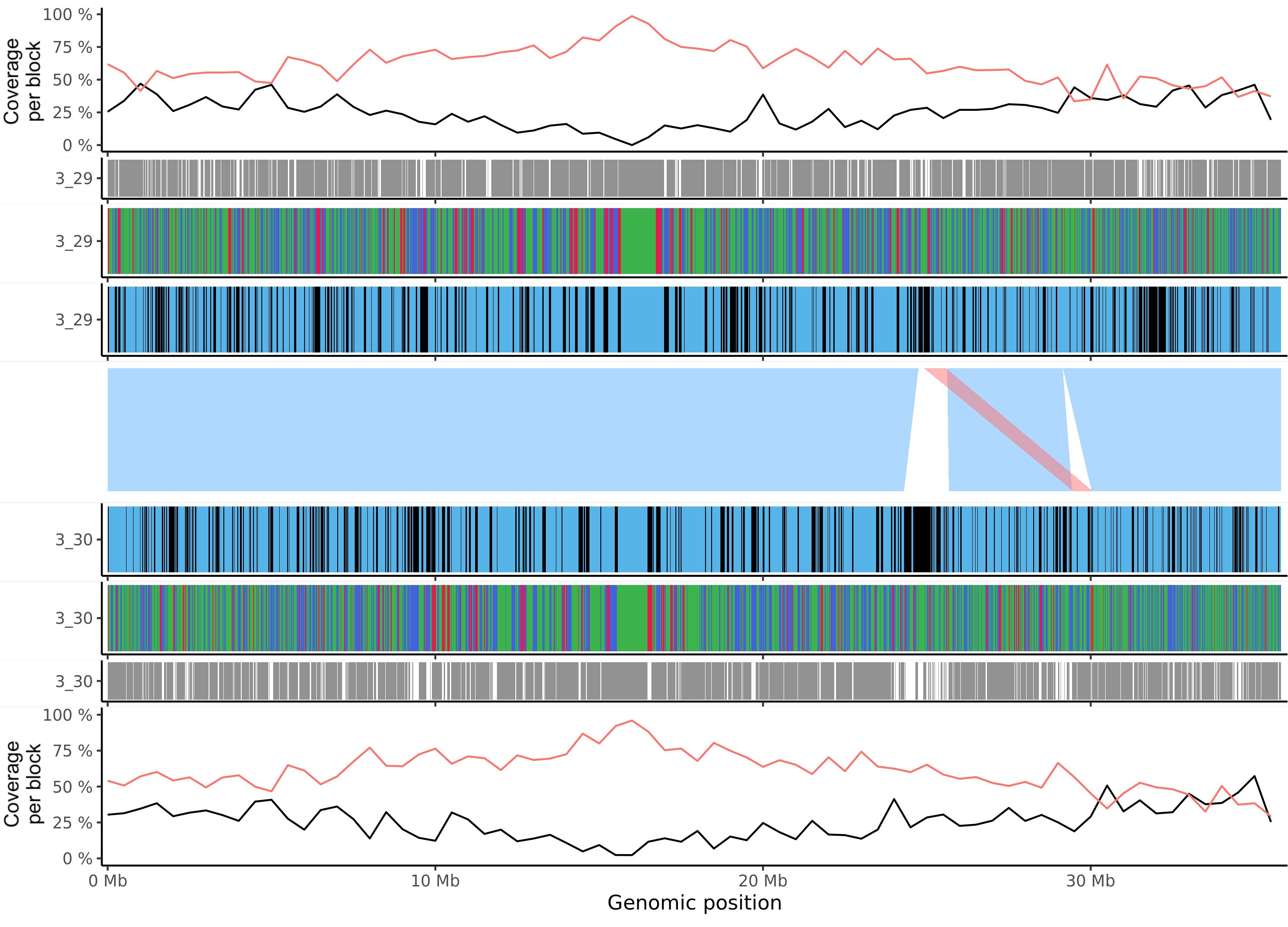

Supplement: Supplementary file 3 — Supplement S3 Supplementary Data. [file PBI-23-874-s002.zip › Supplementary_data/sequence_visualization/Apple/msieversii_chr_7.png]

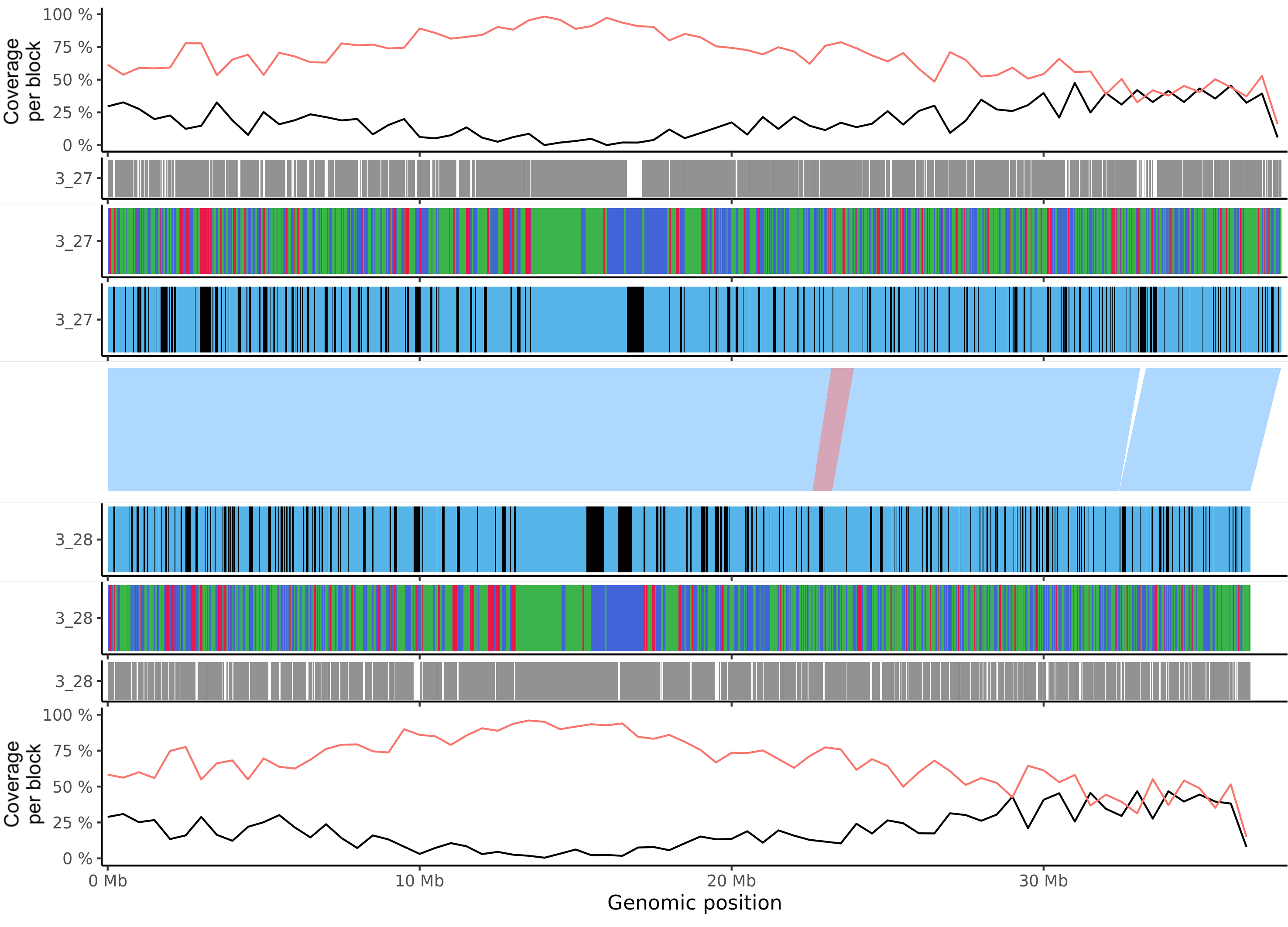

Supplement: Supplementary file 3 — Supplement S3 Supplementary Data. [file PBI-23-874-s002.zip › Supplementary_data/sequence_visualization/Apple/msieversii_chr_6.png]

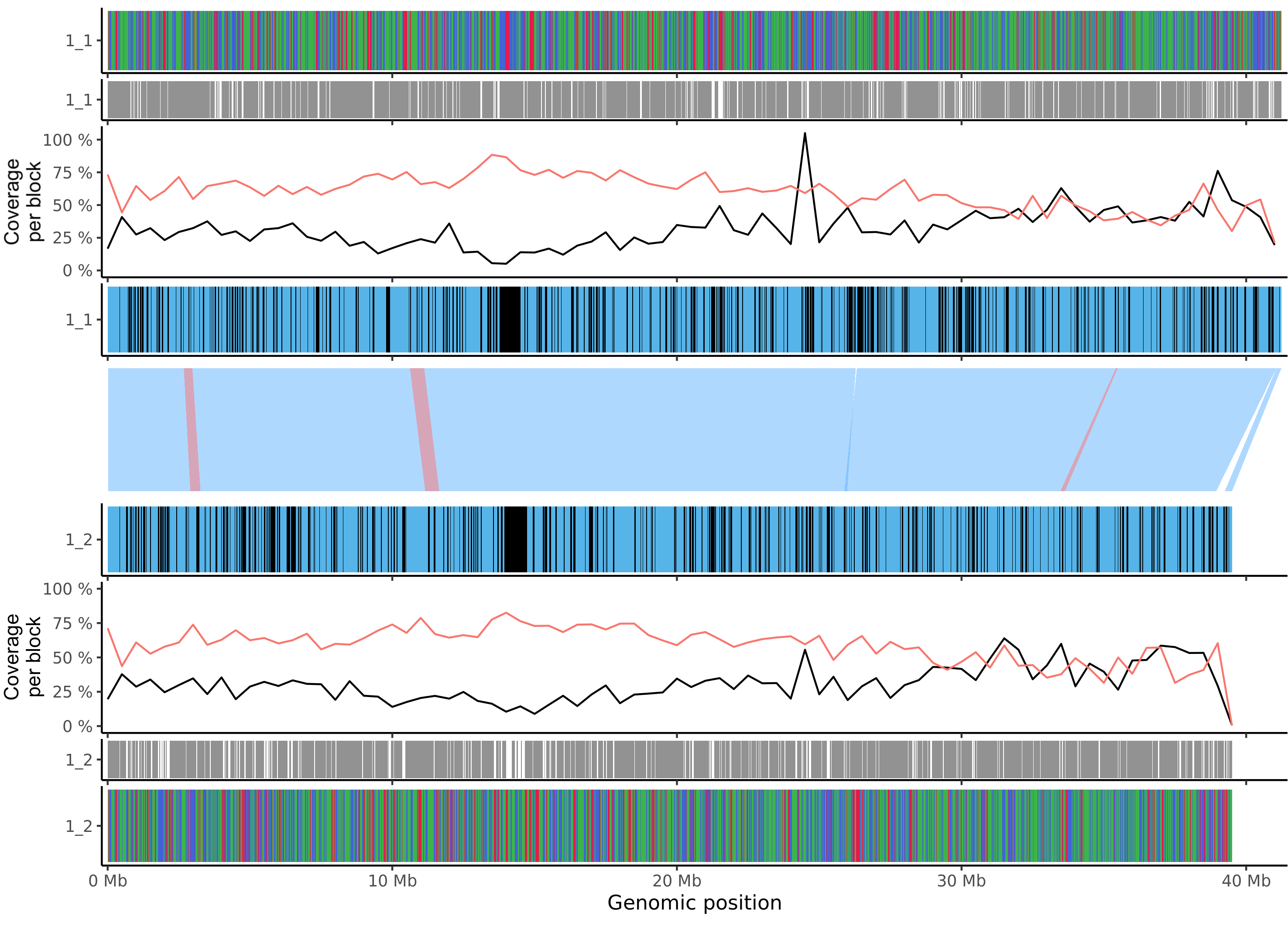

Supplement: Supplementary file 3 — Supplement S3 Supplementary Data. [file PBI-23-874-s002.zip › Supplementary_data/sequence_visualization/Apple/msylvestris_chr_10.png]

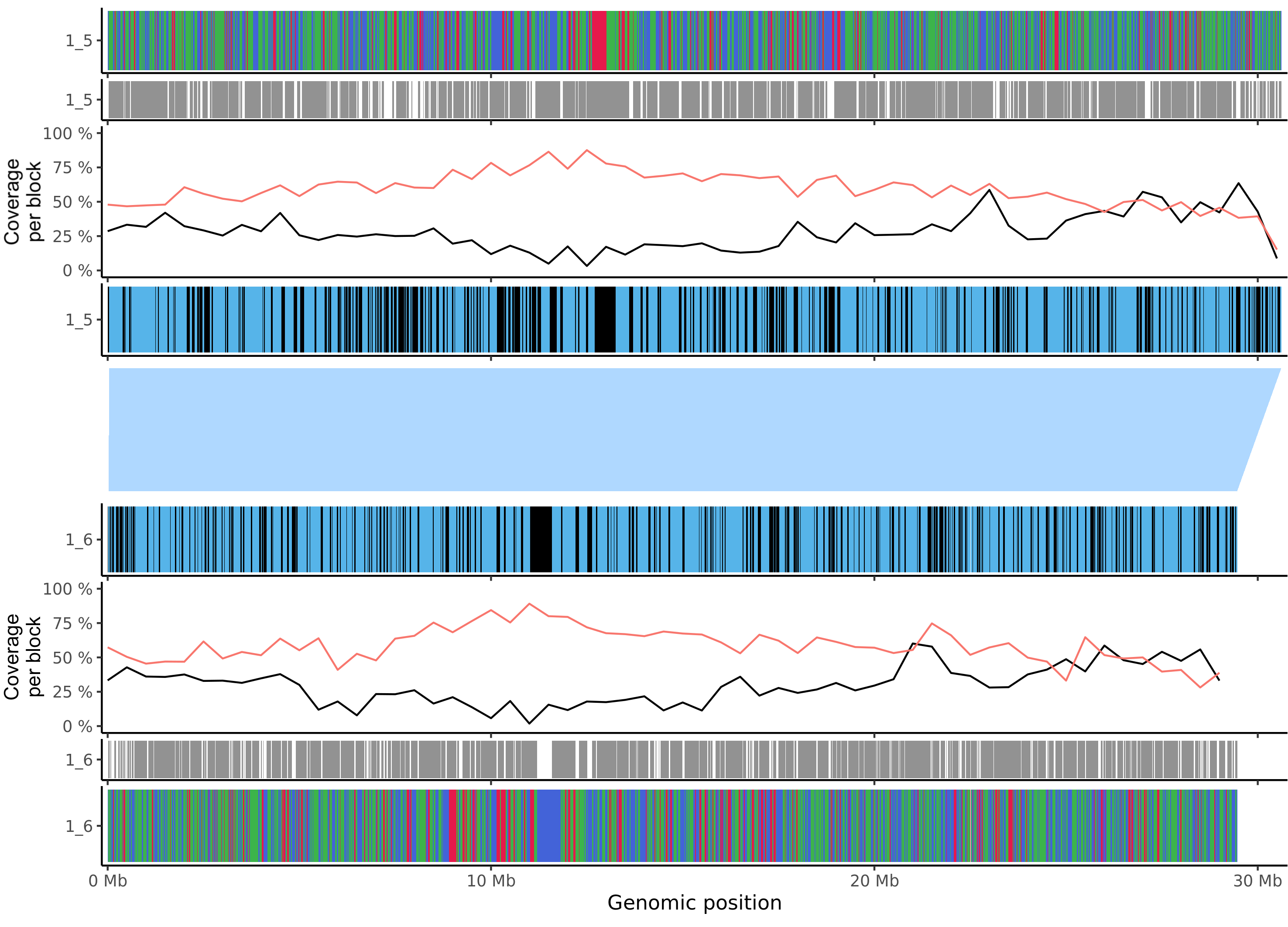

Supplement: Supplementary file 3 — Supplement S3 Supplementary Data. [file PBI-23-874-s002.zip › Supplementary_data/sequence_visualization/Apple/msylvestris_chr_12.png]

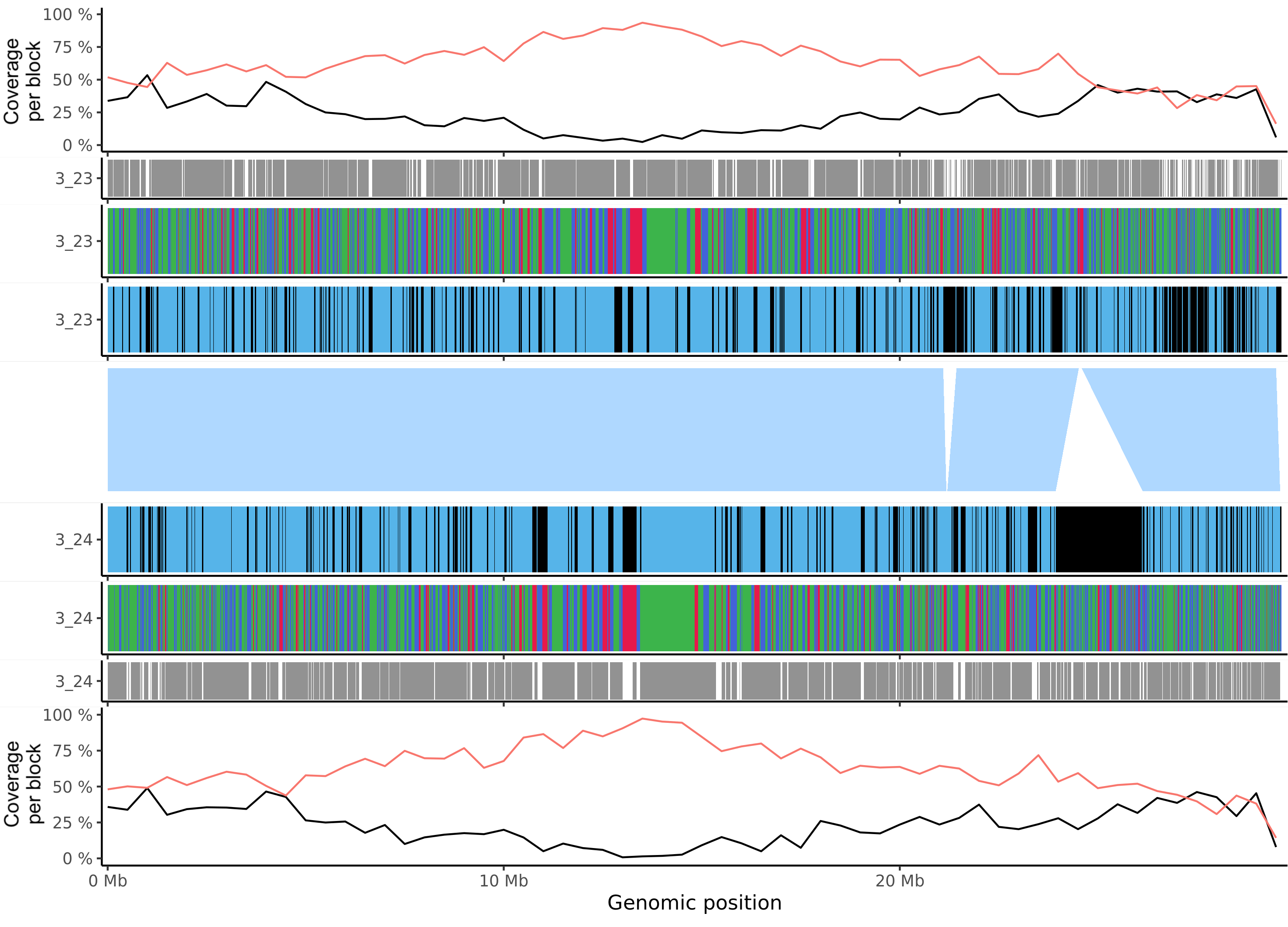

Supplement: Supplementary file 3 — Supplement S3 Supplementary Data. [file PBI-23-874-s002.zip › Supplementary_data/sequence_visualization/Apple/msieversii_chr_4.png]

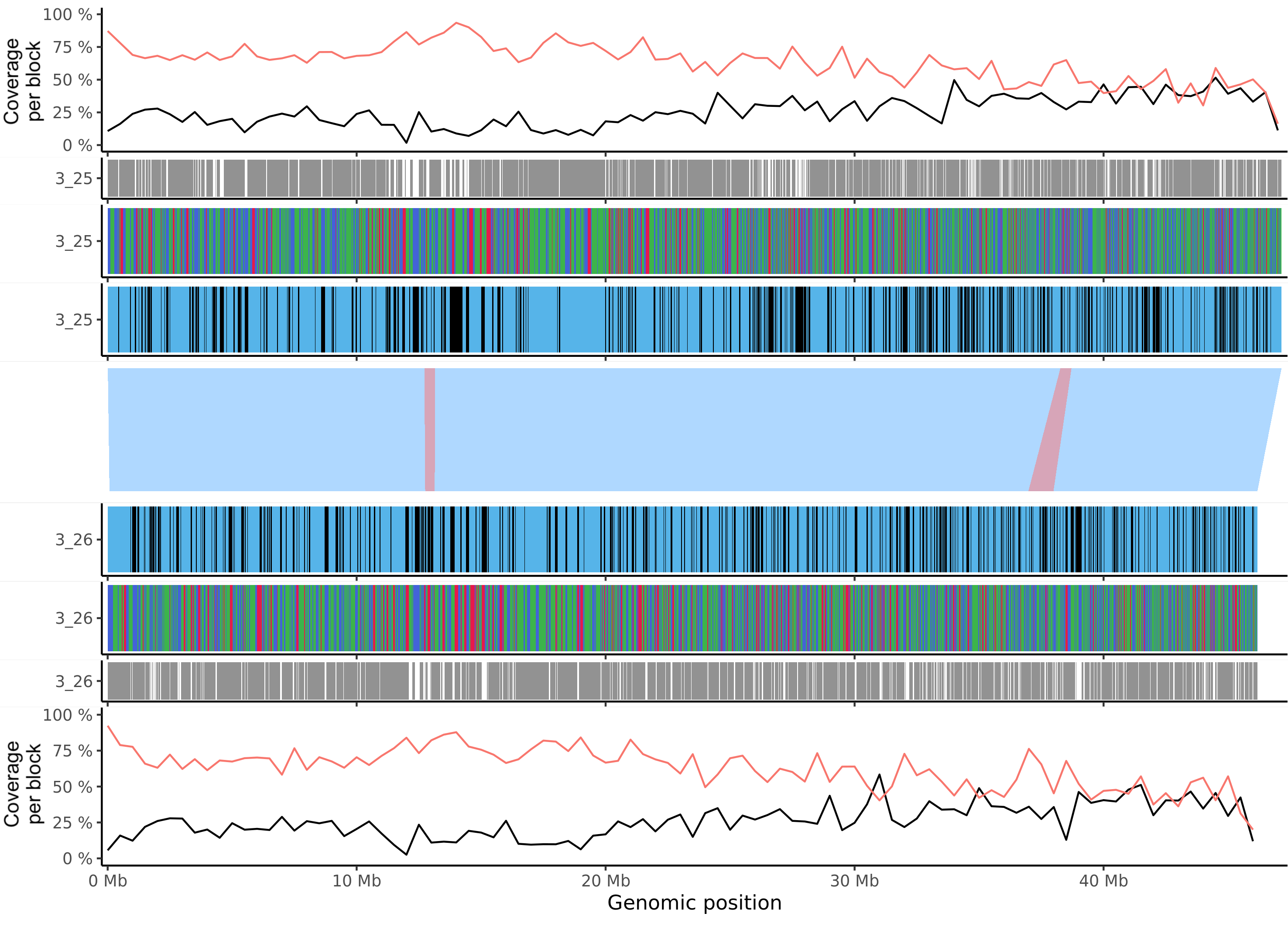

Supplement: Supplementary file 3 — Supplement S3 Supplementary Data. [file PBI-23-874-s002.zip › Supplementary_data/sequence_visualization/Apple/msieversii_chr_5.png]

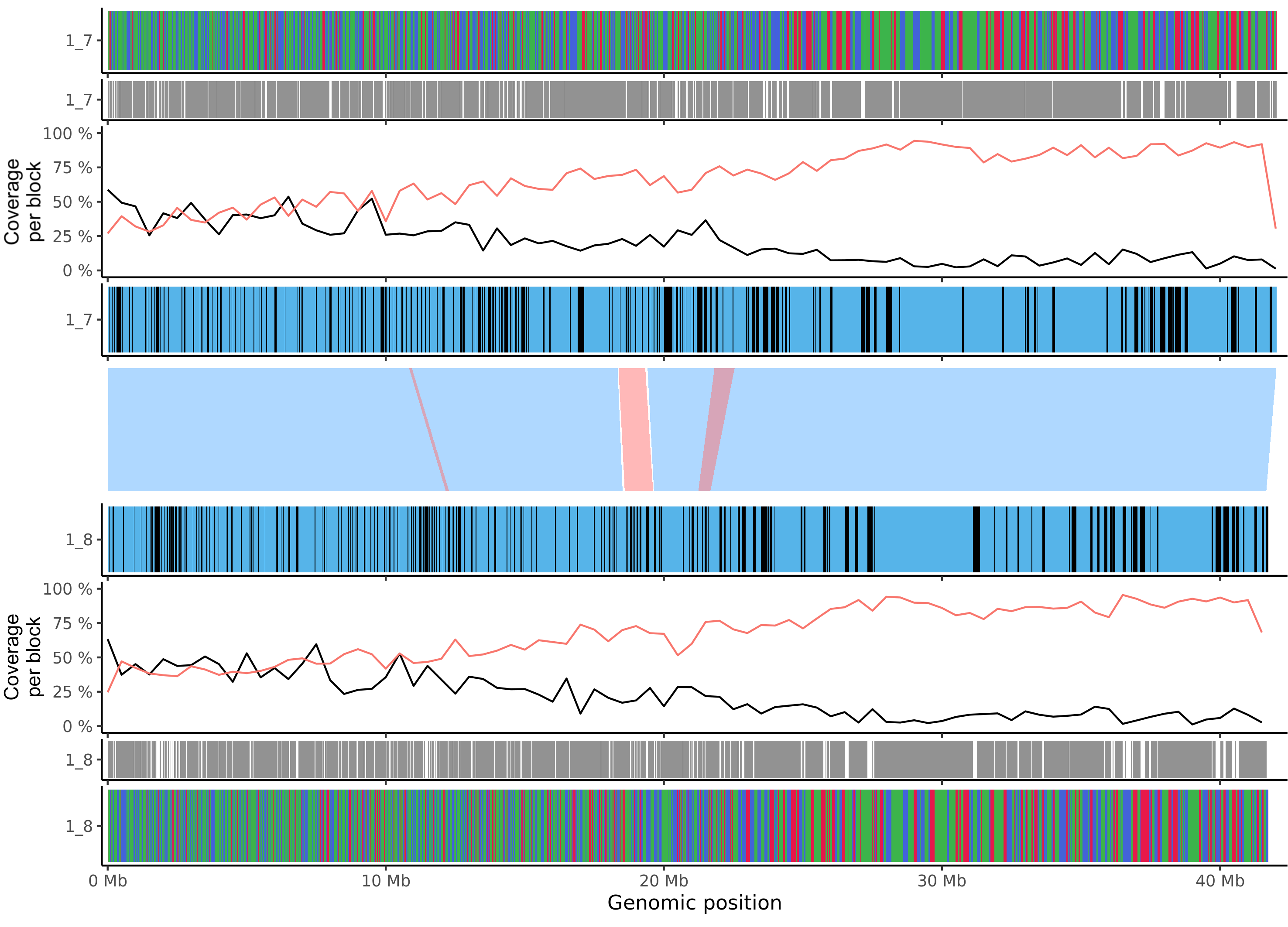

Supplement: Supplementary file 3 — Supplement S3 Supplementary Data. [file PBI-23-874-s002.zip › Supplementary_data/sequence_visualization/Apple/msylvestris_chr_13.png]

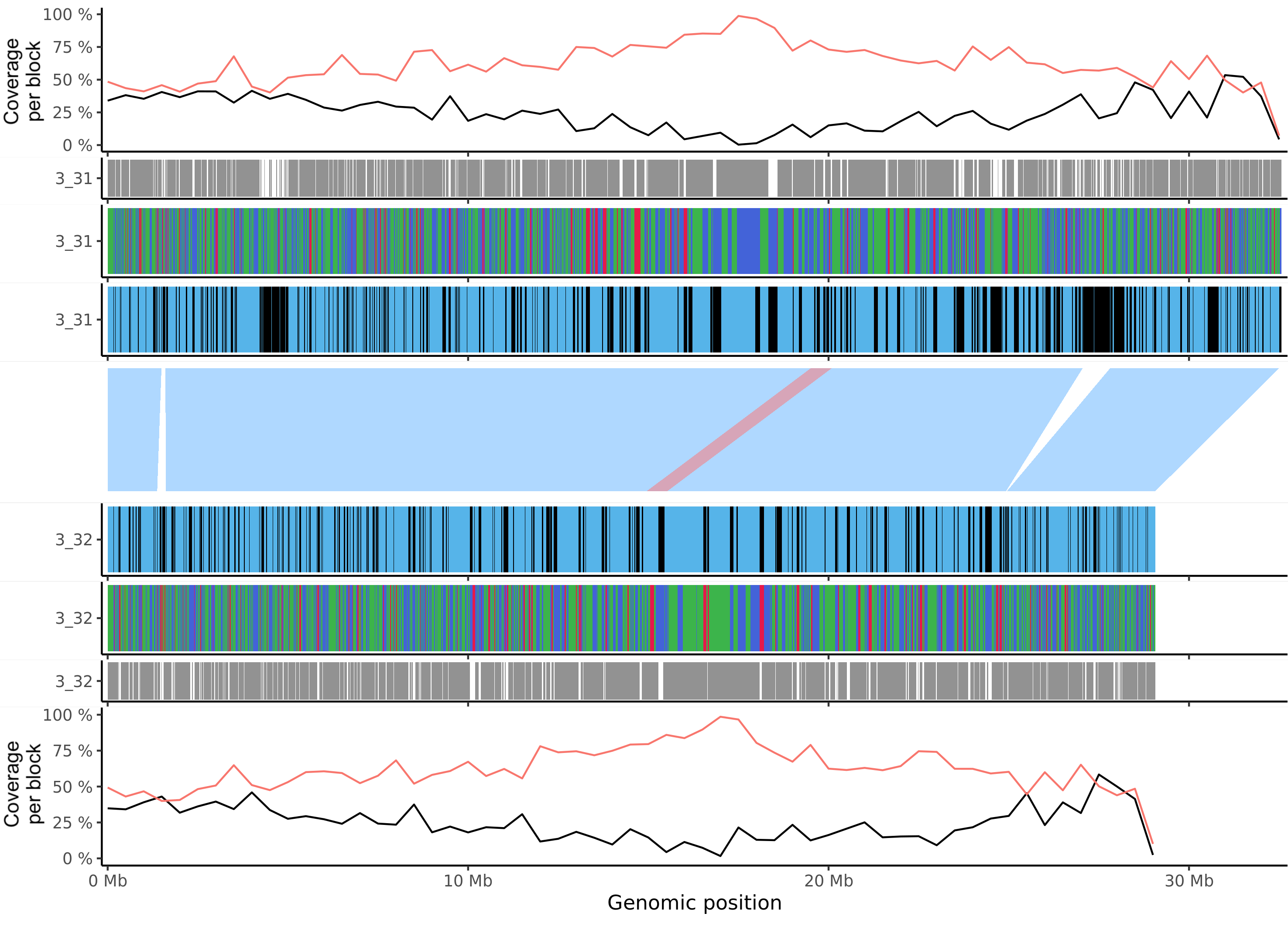

Supplement: Supplementary file 3 — Supplement S3 Supplementary Data. [file PBI-23-874-s002.zip › Supplementary_data/sequence_visualization/Apple/msieversii_chr_8.png]

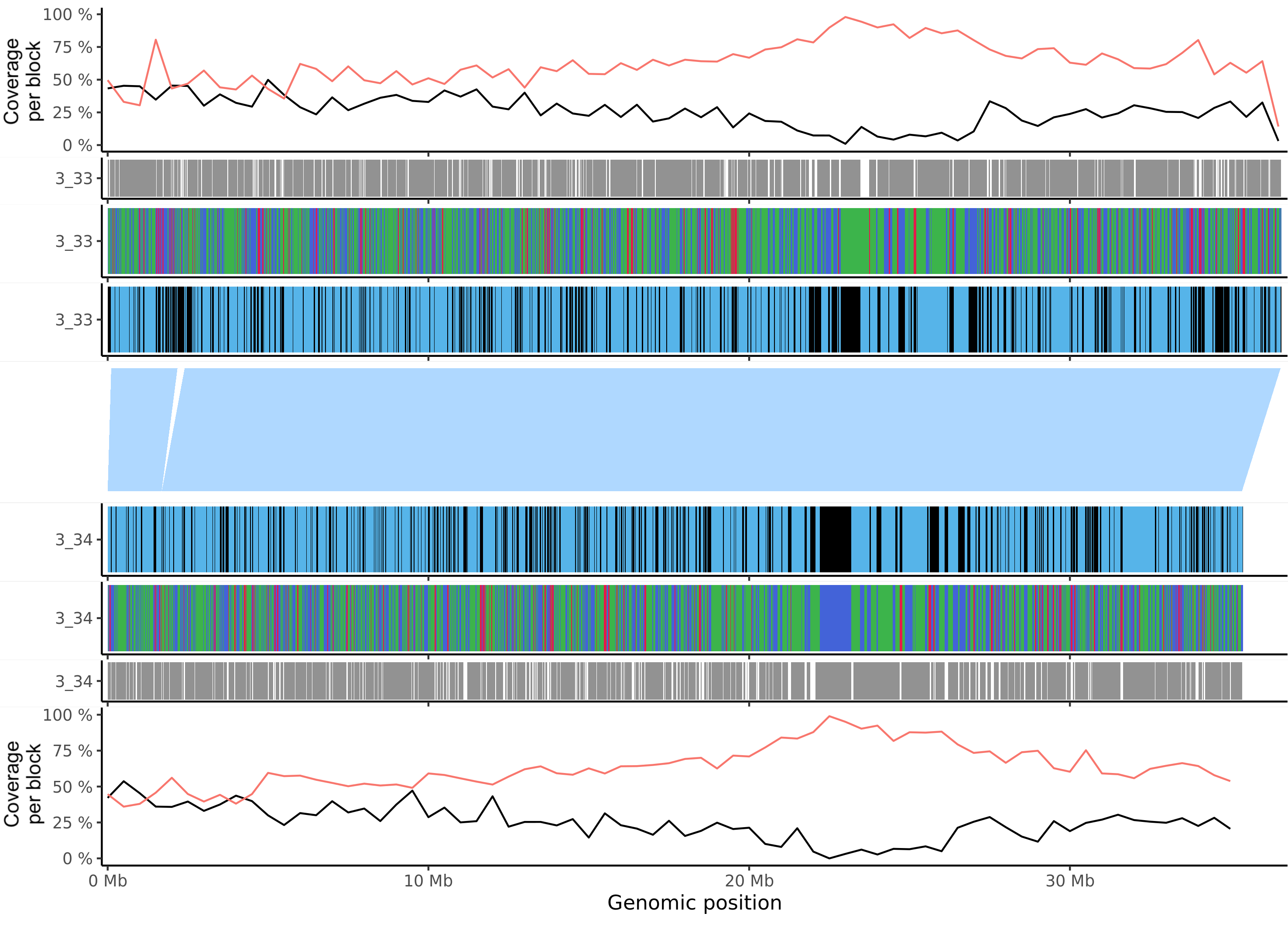

Supplement: Supplementary file 3 — Supplement S3 Supplementary Data. [file PBI-23-874-s002.zip › Supplementary_data/sequence_visualization/Apple/msieversii_chr_9.png]

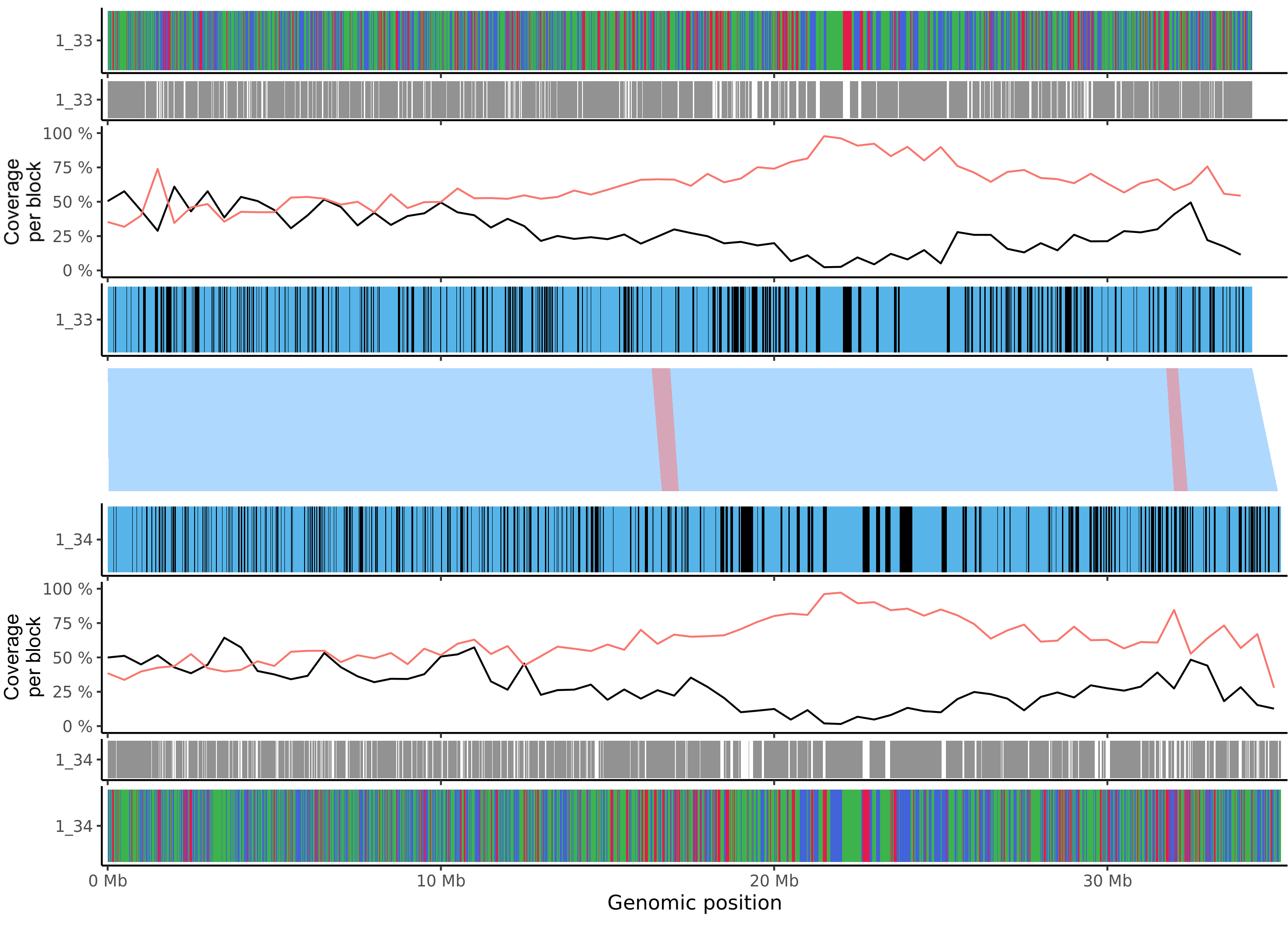

Supplement: Supplementary file 3 — Supplement S3 Supplementary Data. [file PBI-23-874-s002.zip › Supplementary_data/sequence_visualization/Apple/msylvestris_chr_9.png]

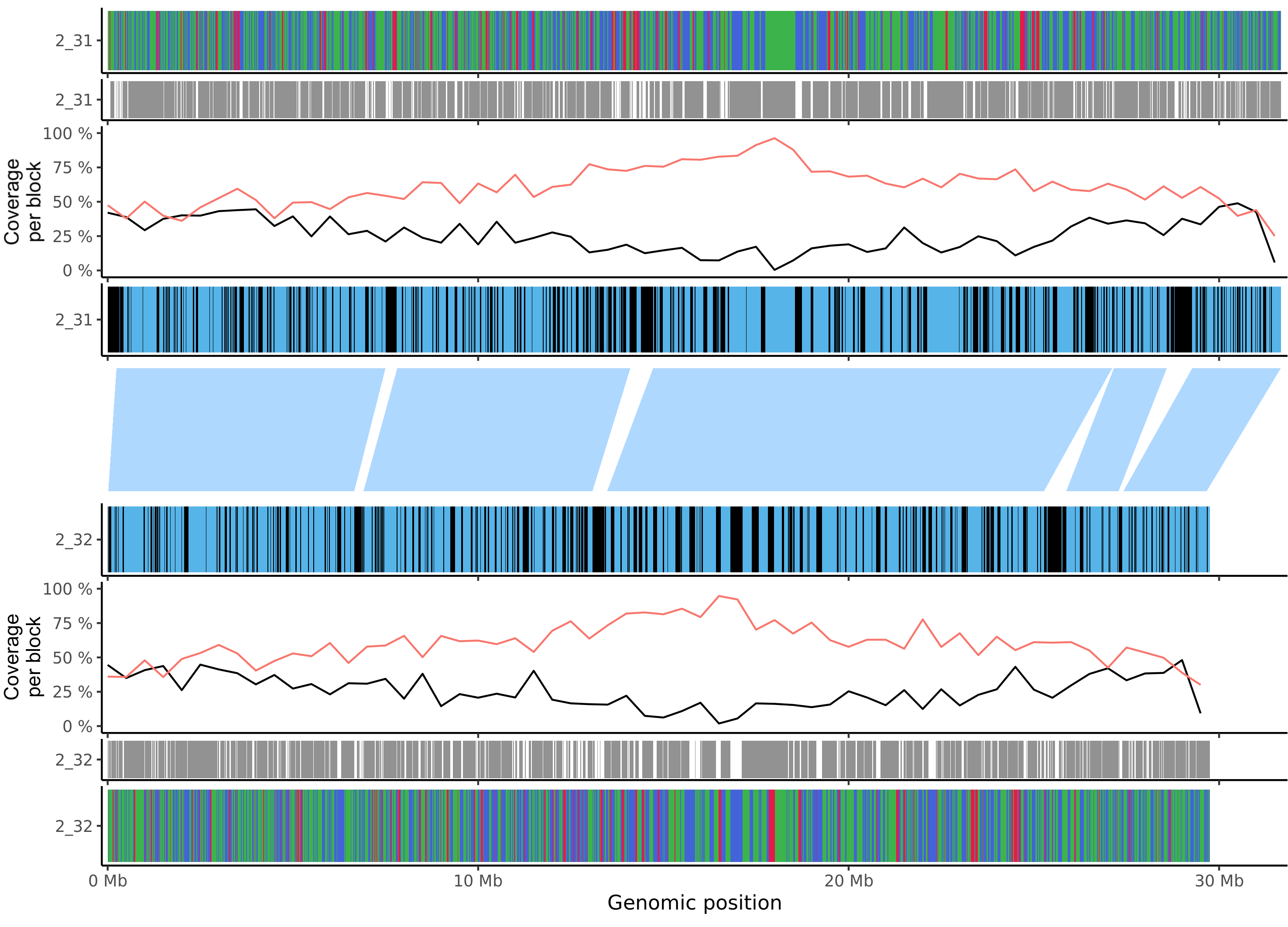

Supplement: Supplementary file 3 — Supplement S3 Supplementary Data. [file PBI-23-874-s002.zip › Supplementary_data/sequence_visualization/Apple/mdomestica_gala_chr_8.png]

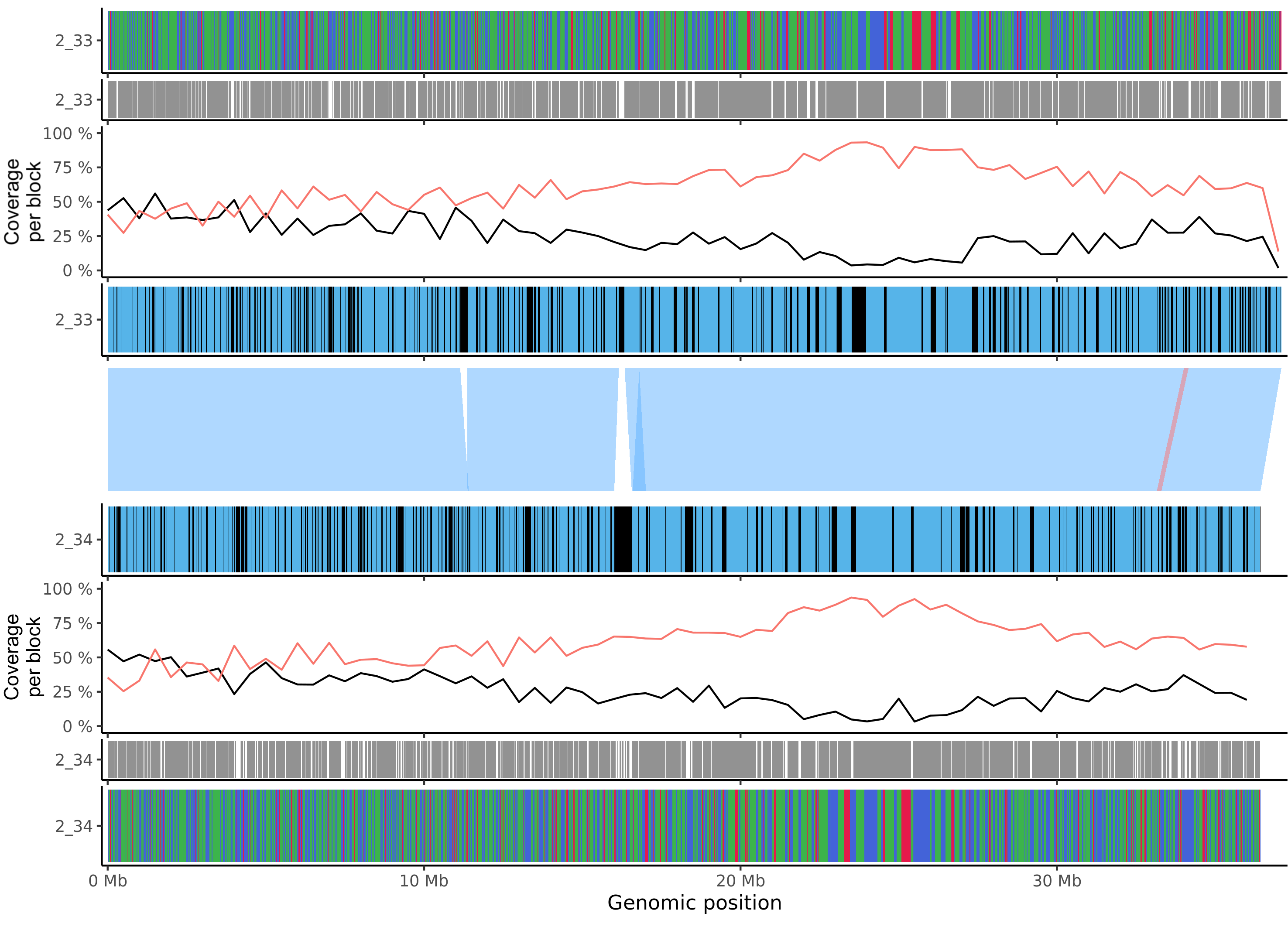

Supplement: Supplementary file 3 — Supplement S3 Supplementary Data. [file PBI-23-874-s002.zip › Supplementary_data/sequence_visualization/Apple/mdomestica_gala_chr_9.png]

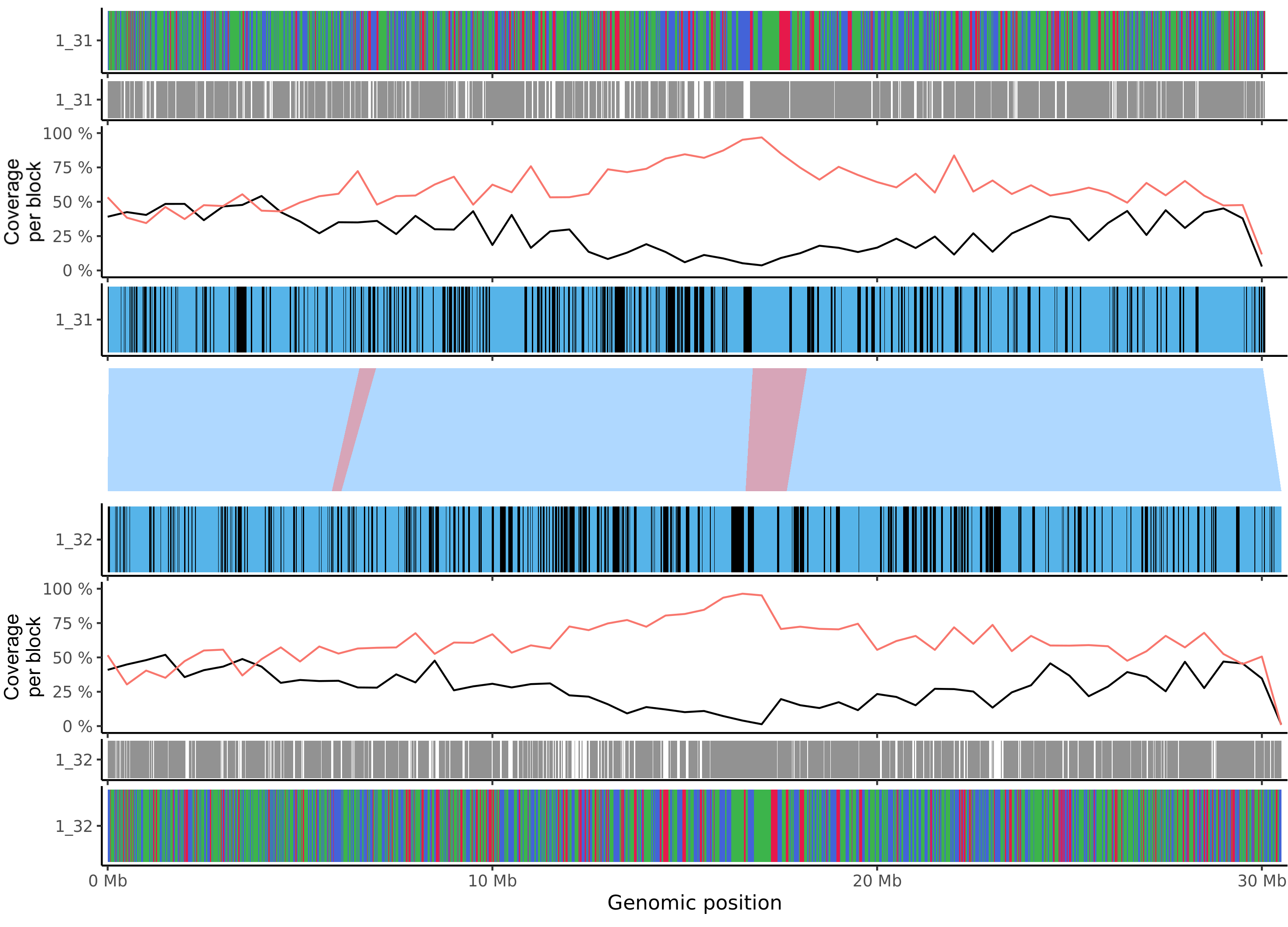

Supplement: Supplementary file 3 — Supplement S3 Supplementary Data. [file PBI-23-874-s002.zip › Supplementary_data/sequence_visualization/Apple/msylvestris_chr_8.png]

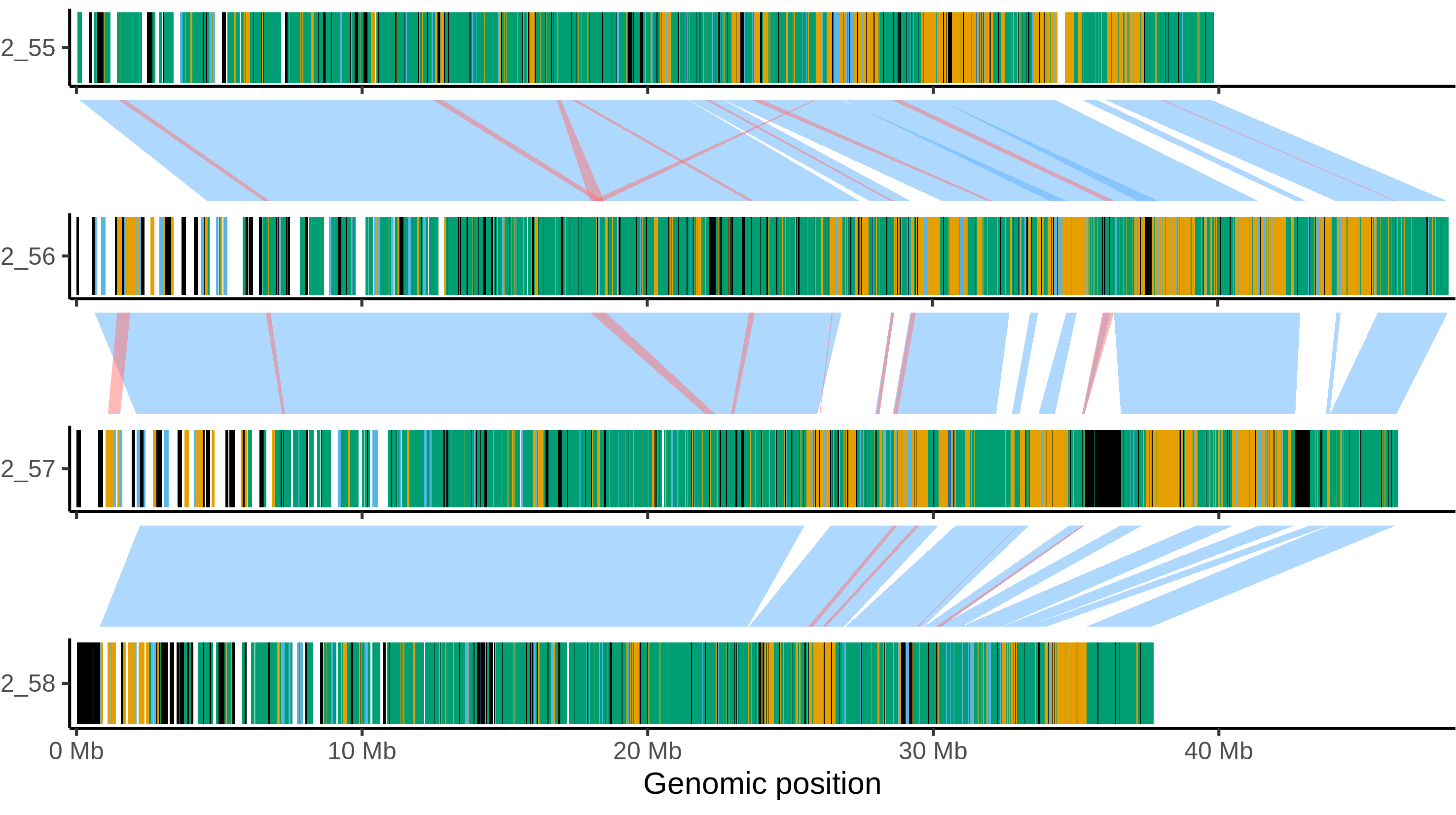

Supplement: Supplementary file 3 — Supplement S3 Supplementary Data. [file PBI-23-874-s002.zip › Supplementary_data/sequence_visualization/Potato/Atlantic_chr_2.png]

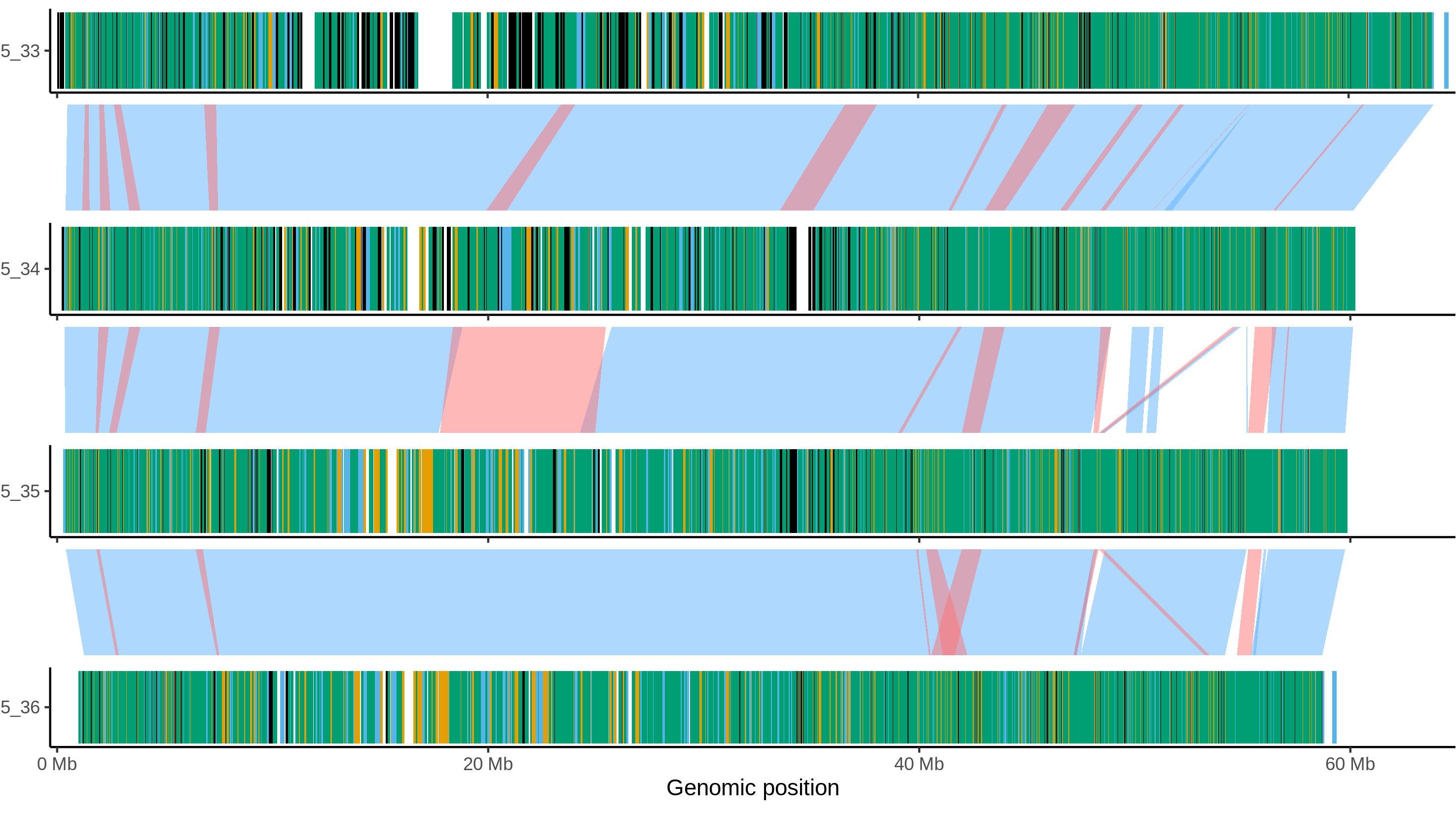

Supplement: Supplementary file 3 — Supplement S3 Supplementary Data. [file PBI-23-874-s002.zip › Supplementary_data/sequence_visualization/Potato/C88_chr_6.png]

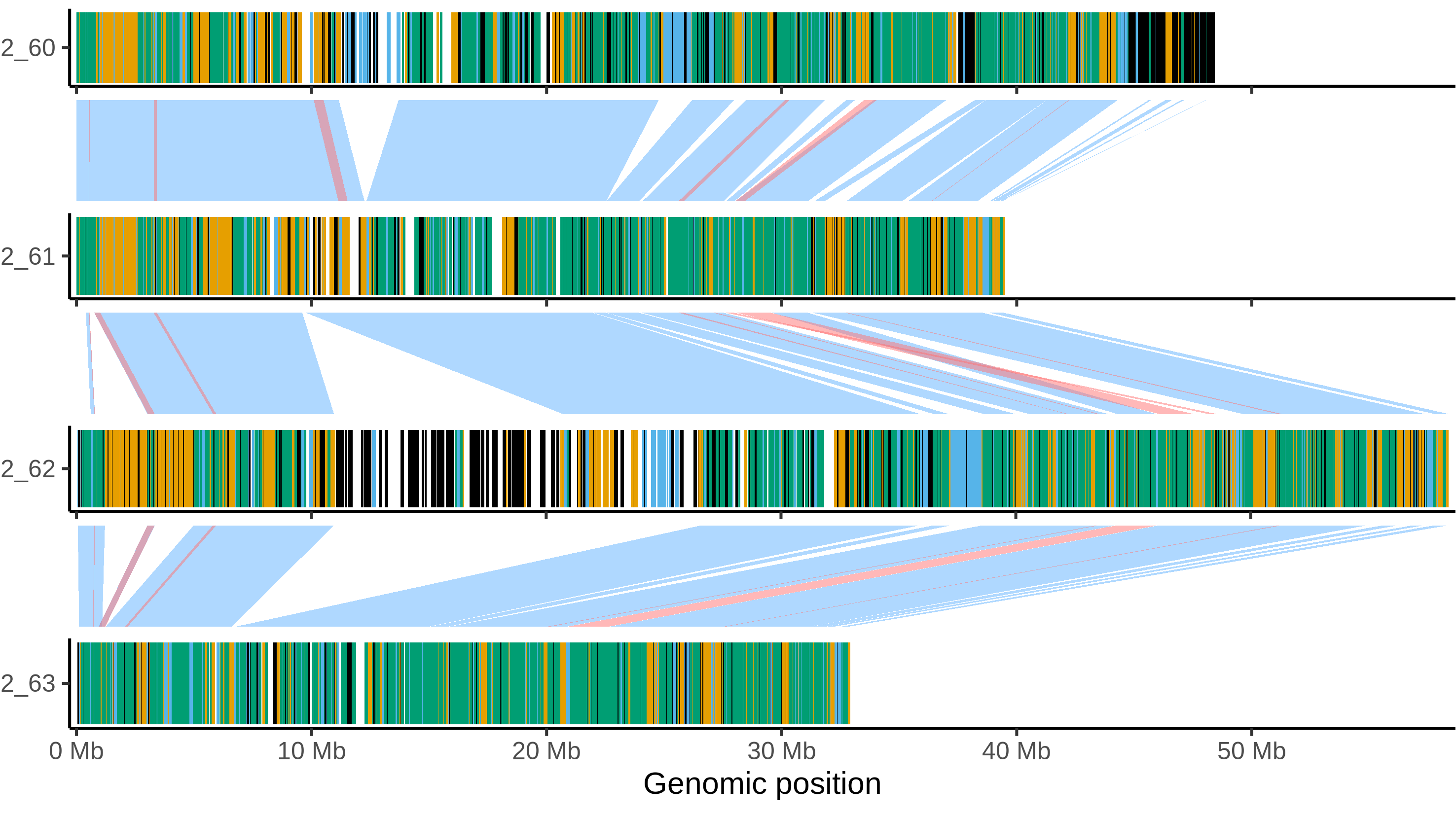

Supplement: Supplementary file 3 — Supplement S3 Supplementary Data. [file PBI-23-874-s002.zip › Supplementary_data/sequence_visualization/Potato/Atlantic_chr_3.png]

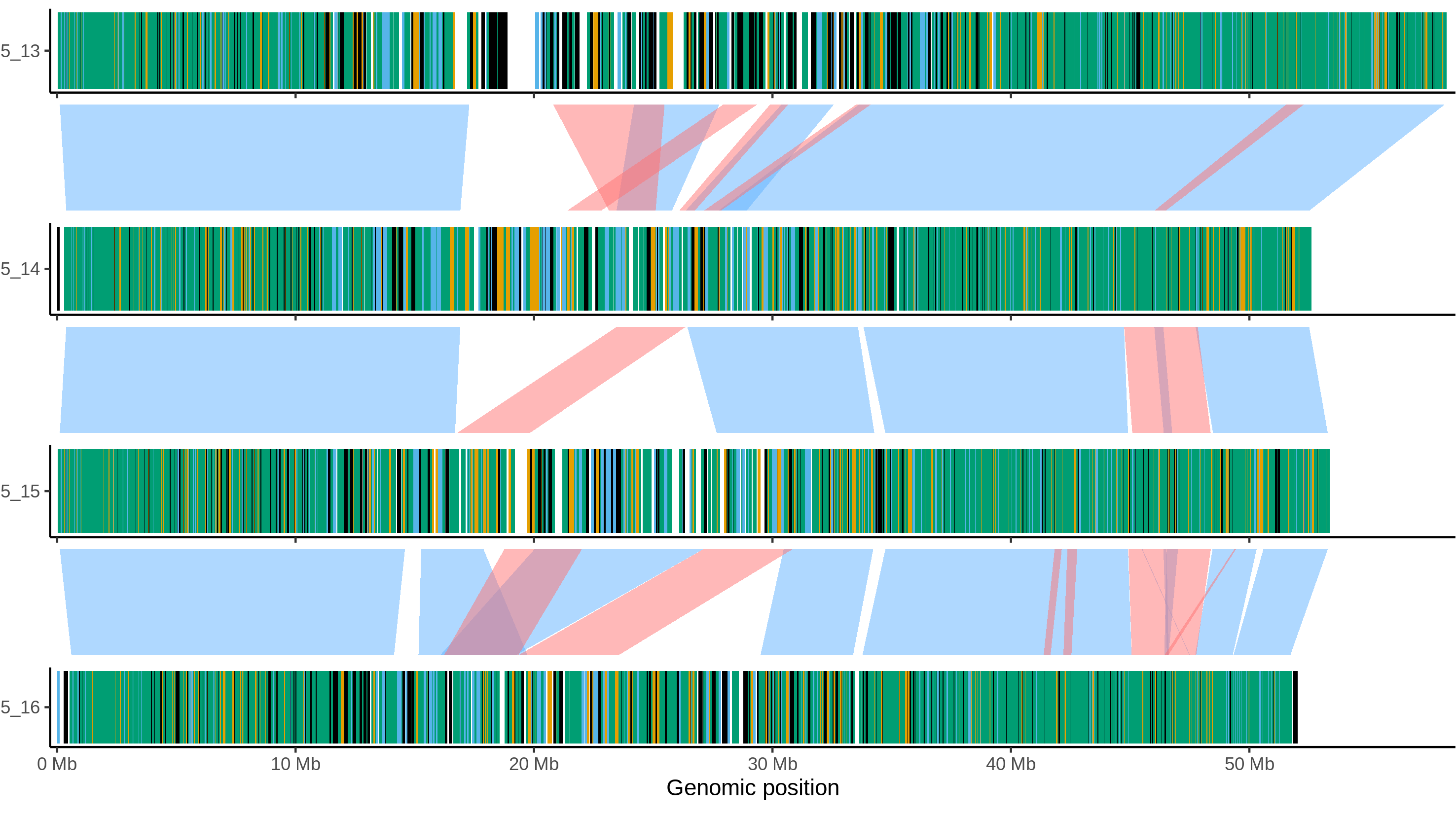

Supplement: Supplementary file 3 — Supplement S3 Supplementary Data. [file PBI-23-874-s002.zip › Supplementary_data/sequence_visualization/Potato/C88_chr_7.png]

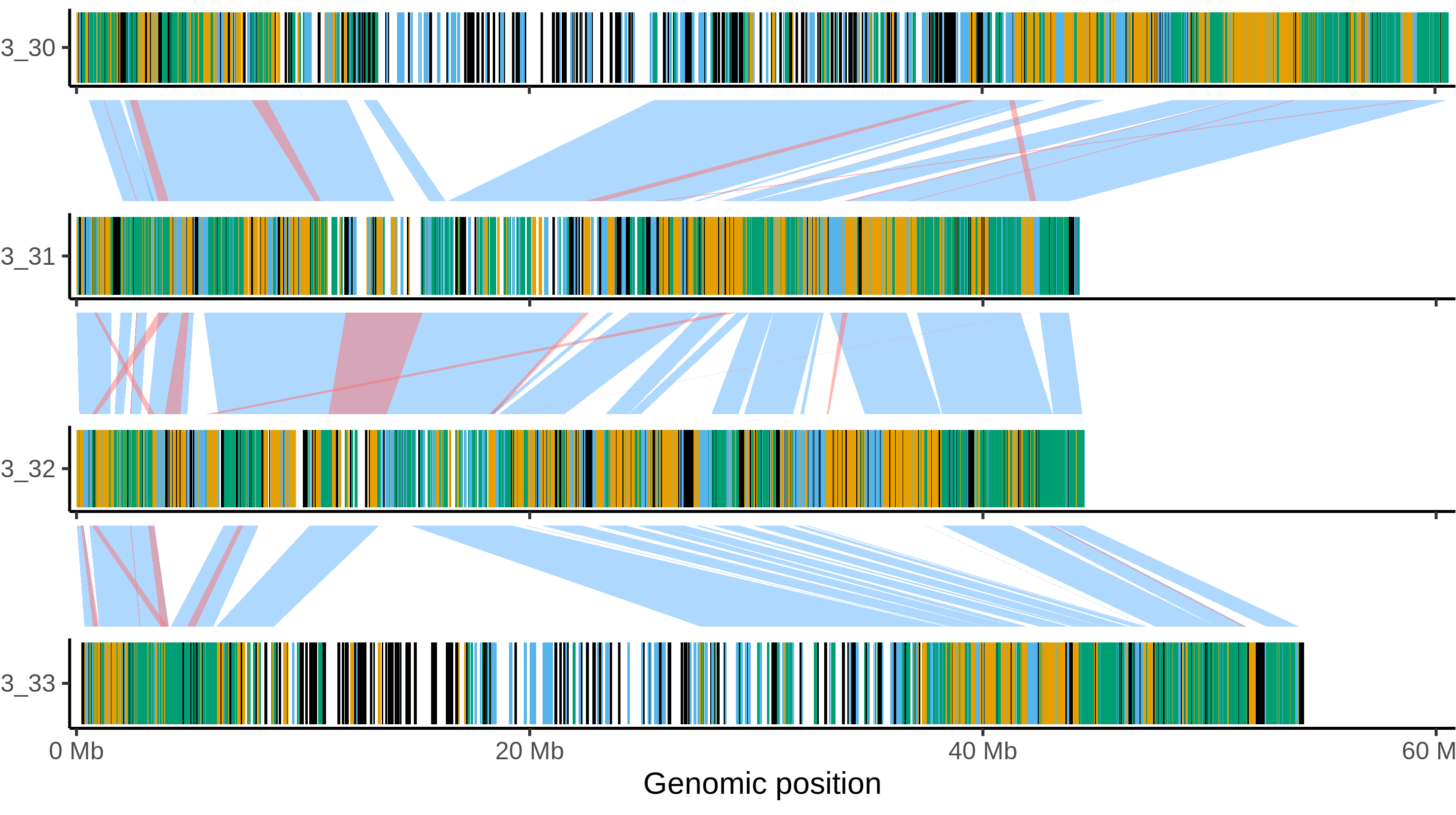

Supplement: Supplementary file 3 — Supplement S3 Supplementary Data. [file PBI-23-874-s002.zip › Supplementary_data/sequence_visualization/Potato/Castle_russet_chr_1.png]

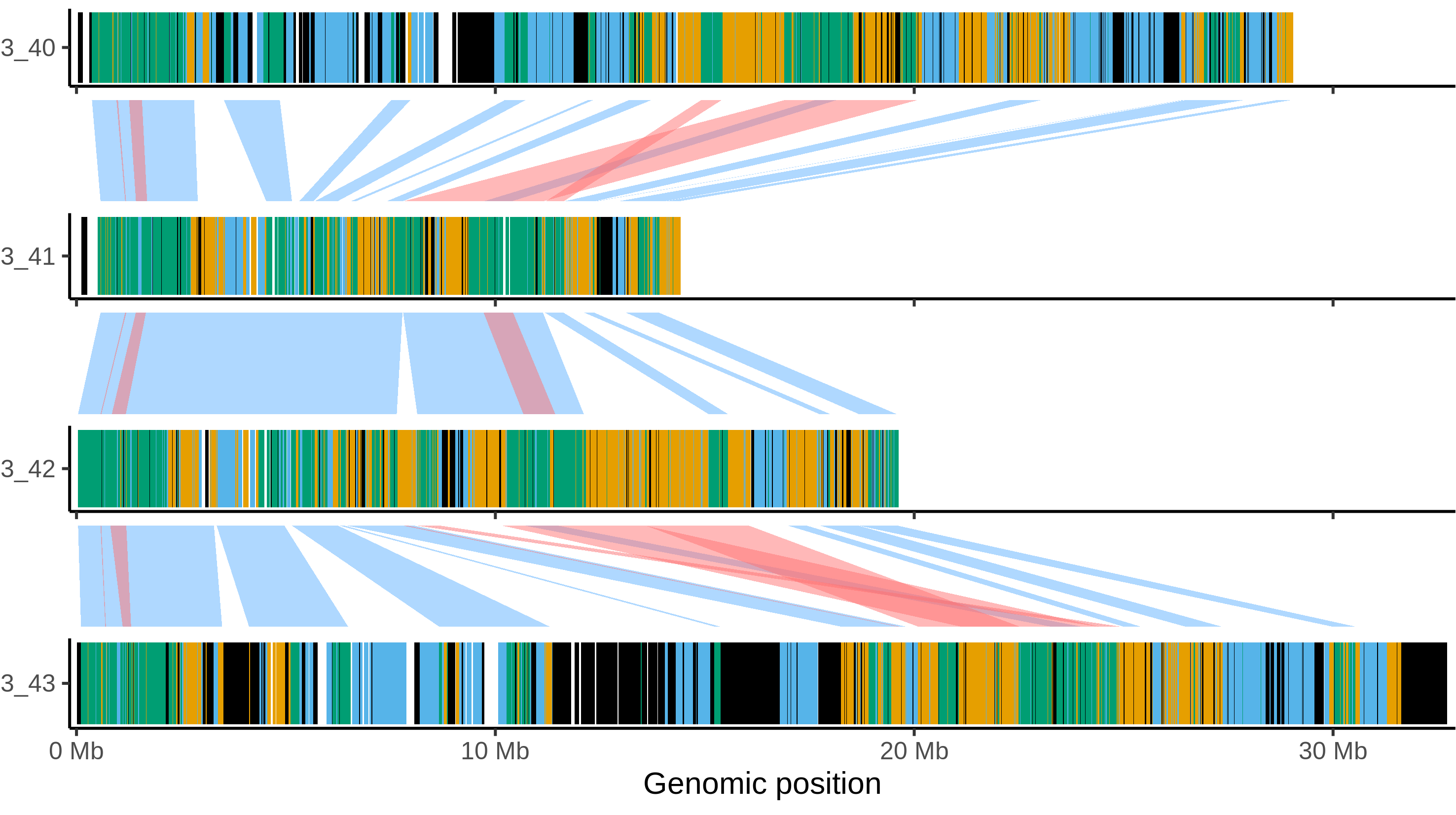

Supplement: Supplementary file 3 — Supplement S3 Supplementary Data. [file PBI-23-874-s002.zip › Supplementary_data/sequence_visualization/Potato/Castle_russet_chr_3.png]

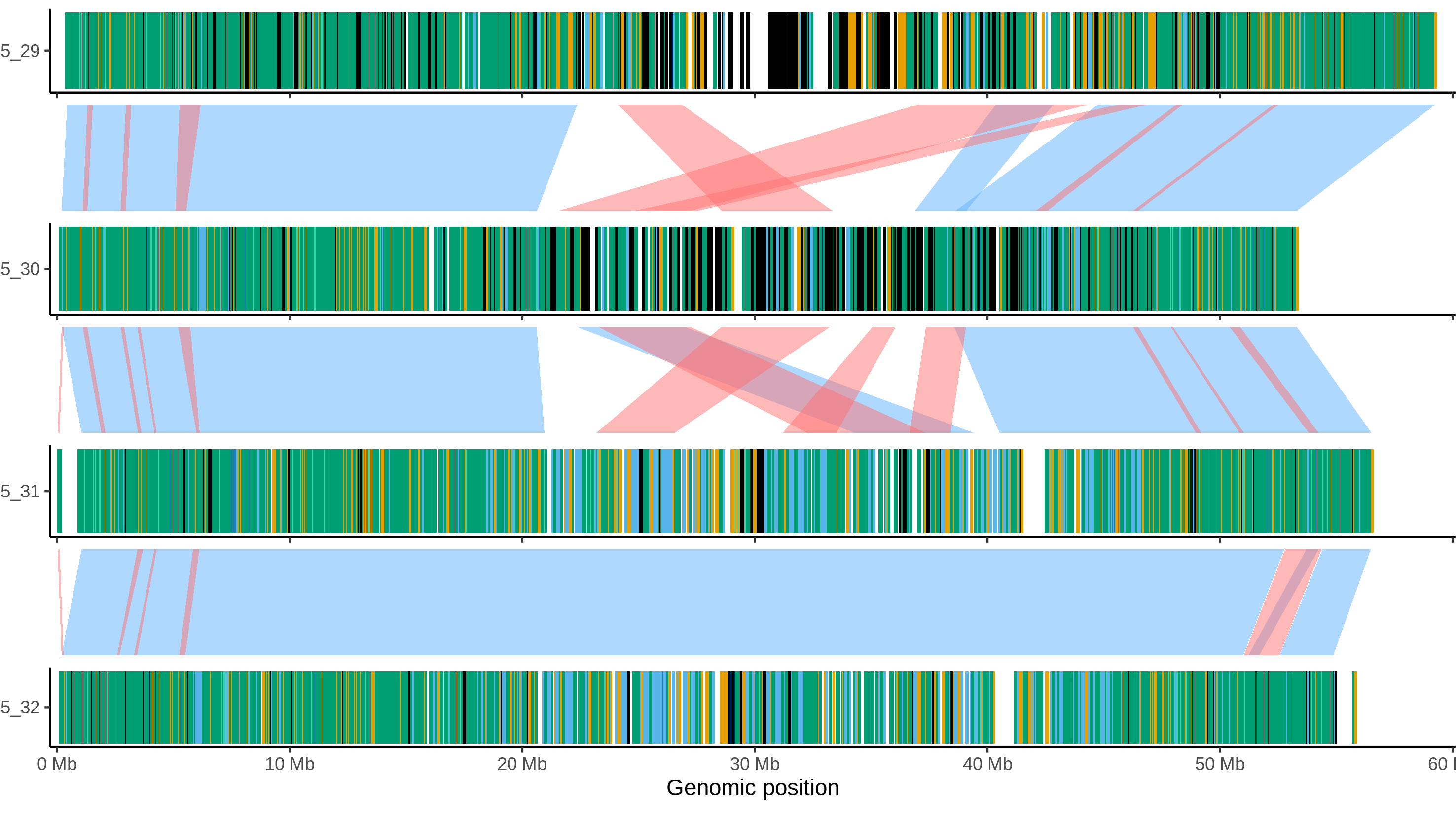

Supplement: Supplementary file 3 — Supplement S3 Supplementary Data. [file PBI-23-874-s002.zip › Supplementary_data/sequence_visualization/Potato/C88_chr_5.png]

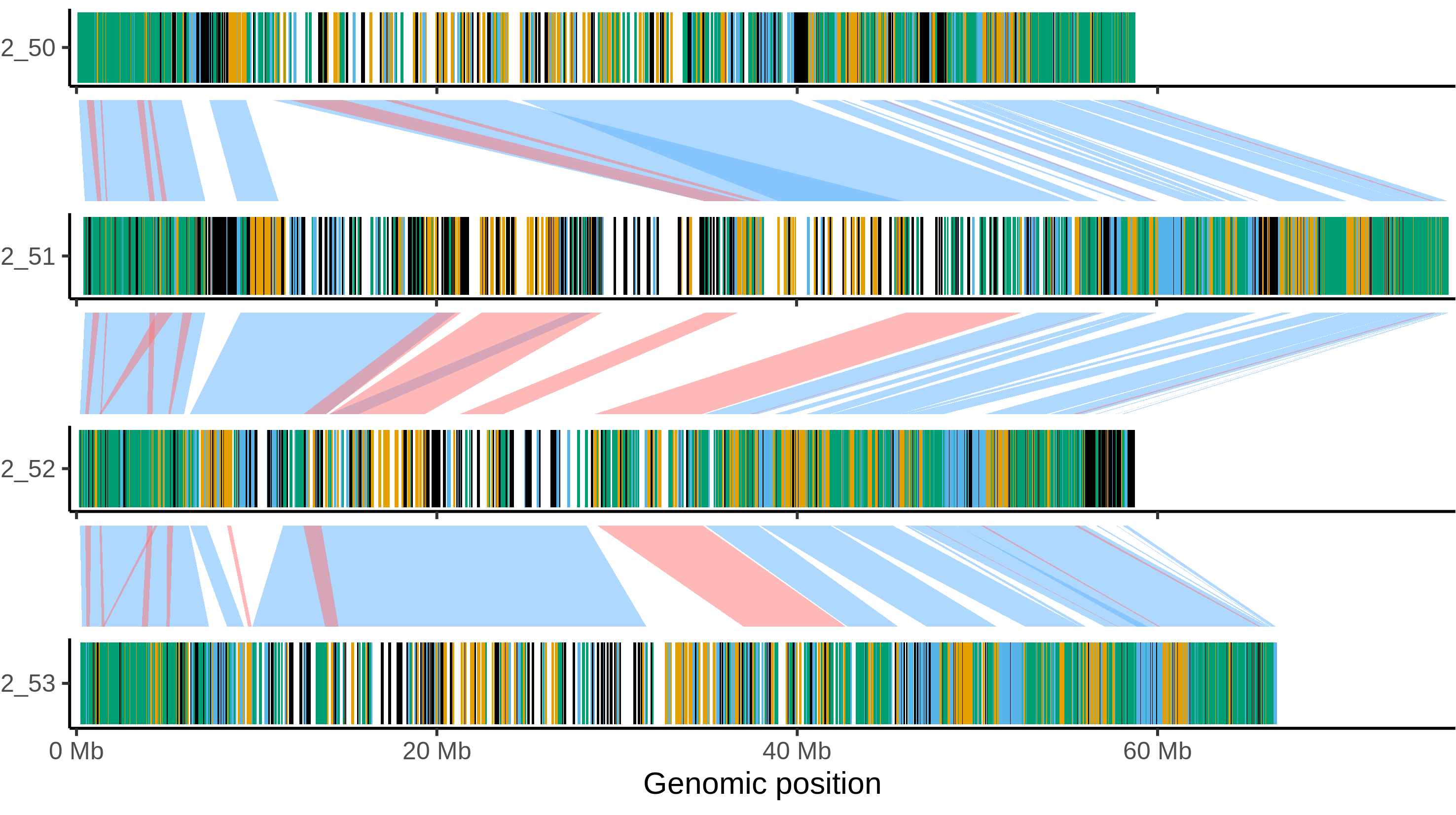

Supplement: Supplementary file 3 — Supplement S3 Supplementary Data. [file PBI-23-874-s002.zip › Supplementary_data/sequence_visualization/Potato/Atlantic_chr_1.png]

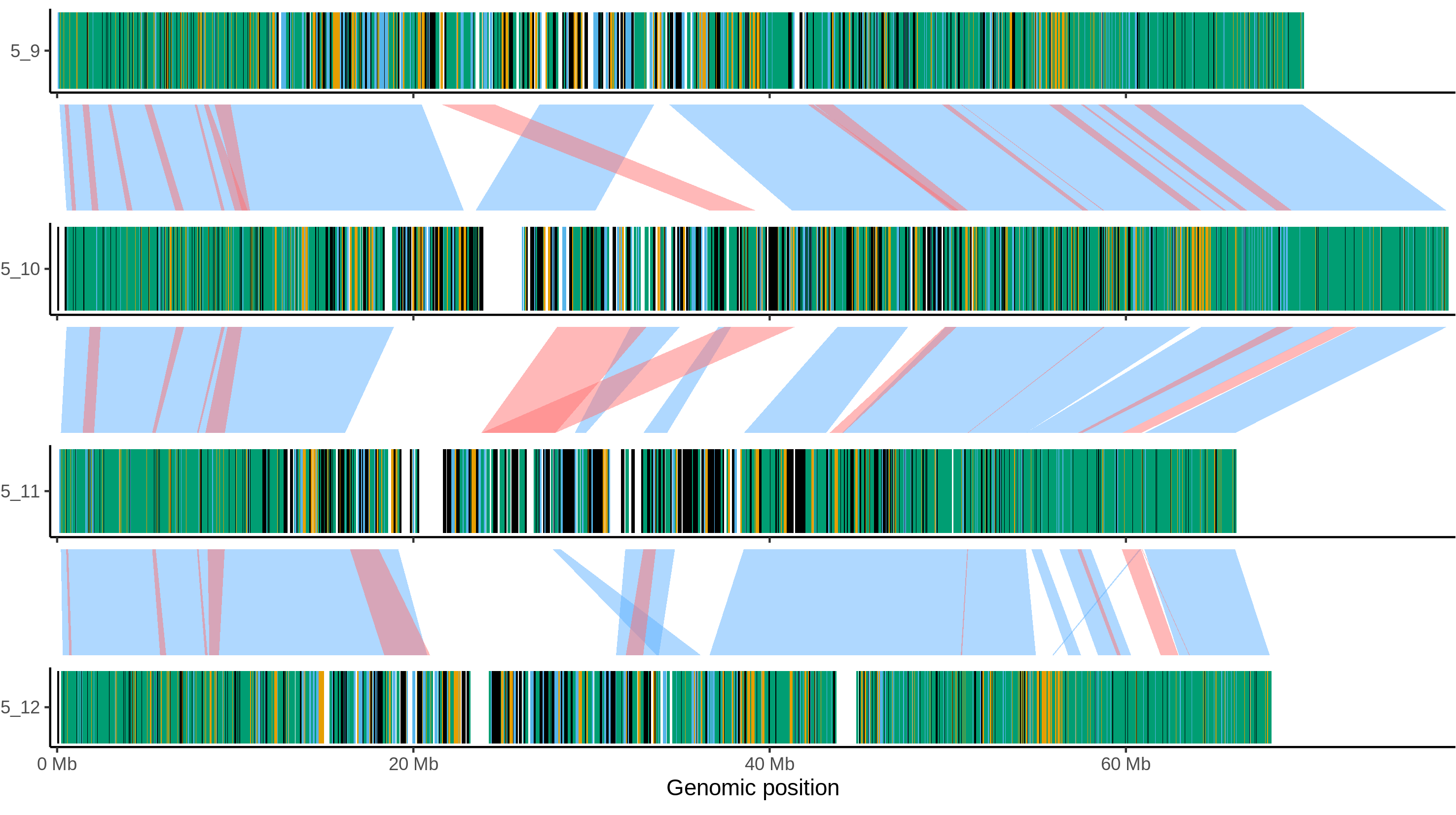

Supplement: Supplementary file 3 — Supplement S3 Supplementary Data. [file PBI-23-874-s002.zip › Supplementary_data/sequence_visualization/Potato/C88_chr_4.png]

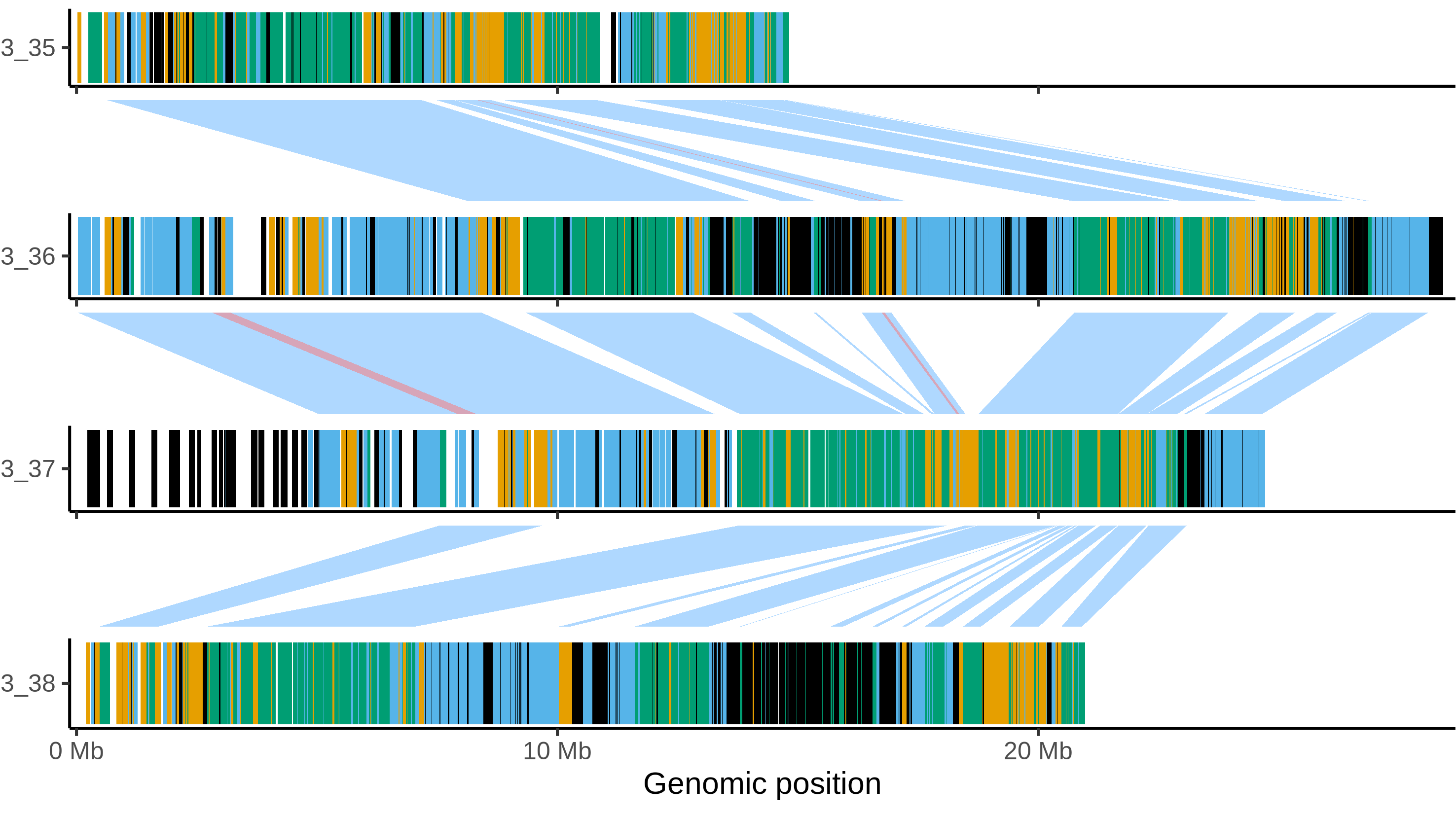

Supplement: Supplementary file 3 — Supplement S3 Supplementary Data. [file PBI-23-874-s002.zip › Supplementary_data/sequence_visualization/Potato/Castle_russet_chr_2.png]

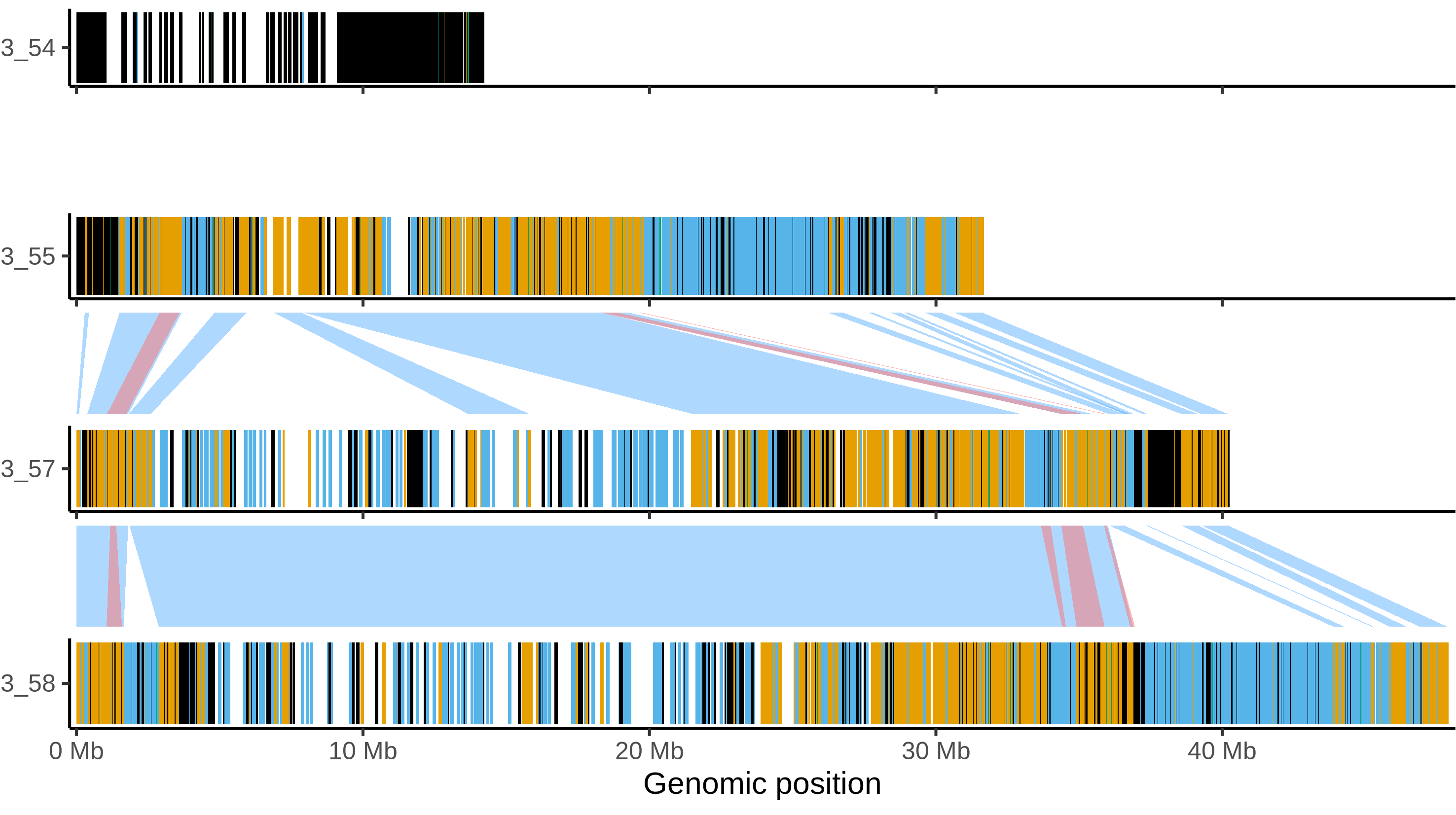

Supplement: Supplementary file 3 — Supplement S3 Supplementary Data. [file PBI-23-874-s002.zip › Supplementary_data/sequence_visualization/Potato/Castle_russet_chr_6.png]

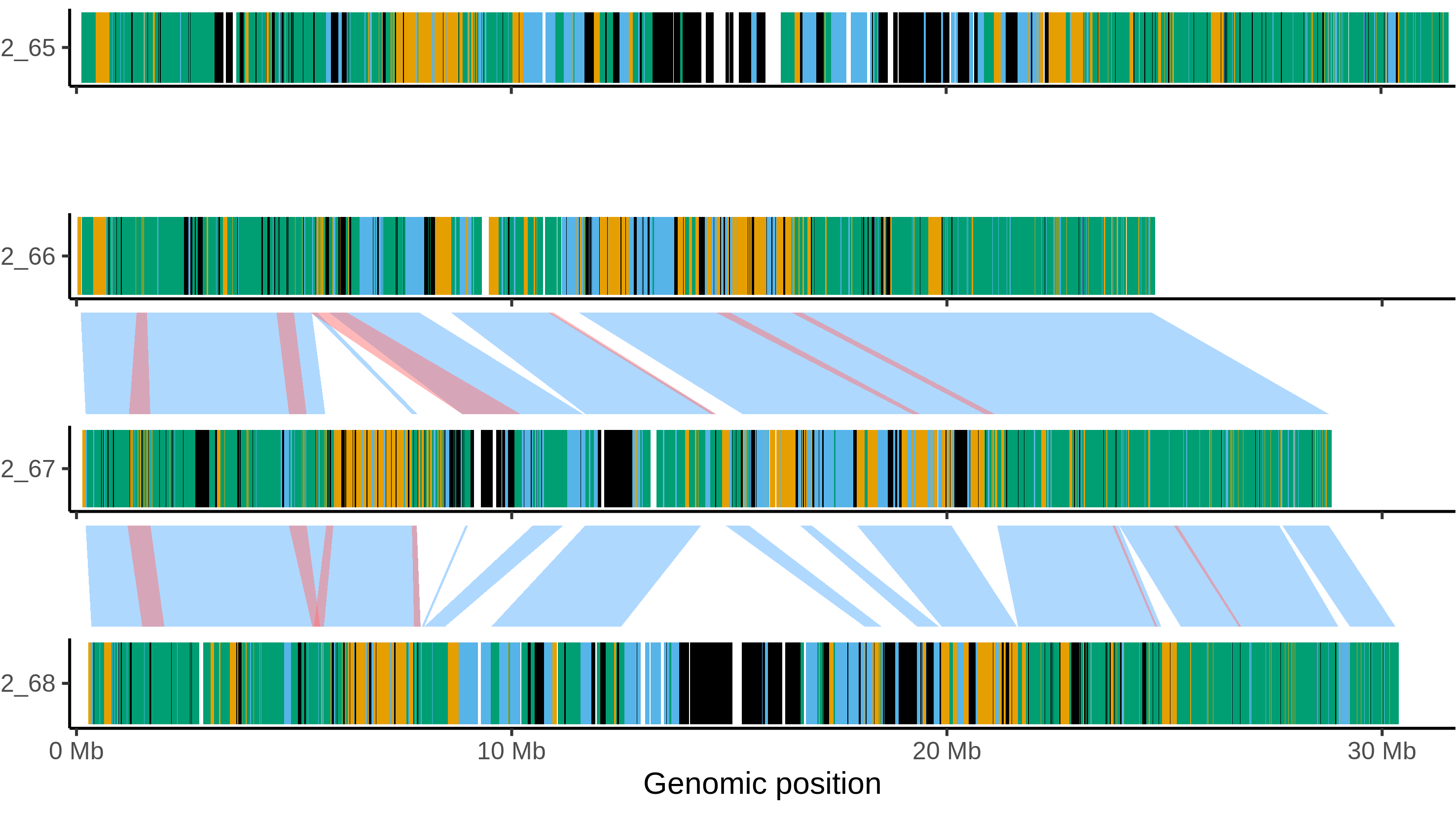

Supplement: Supplementary file 3 — Supplement S3 Supplementary Data. [file PBI-23-874-s002.zip › Supplementary_data/sequence_visualization/Potato/Atlantic_chr_4.png]

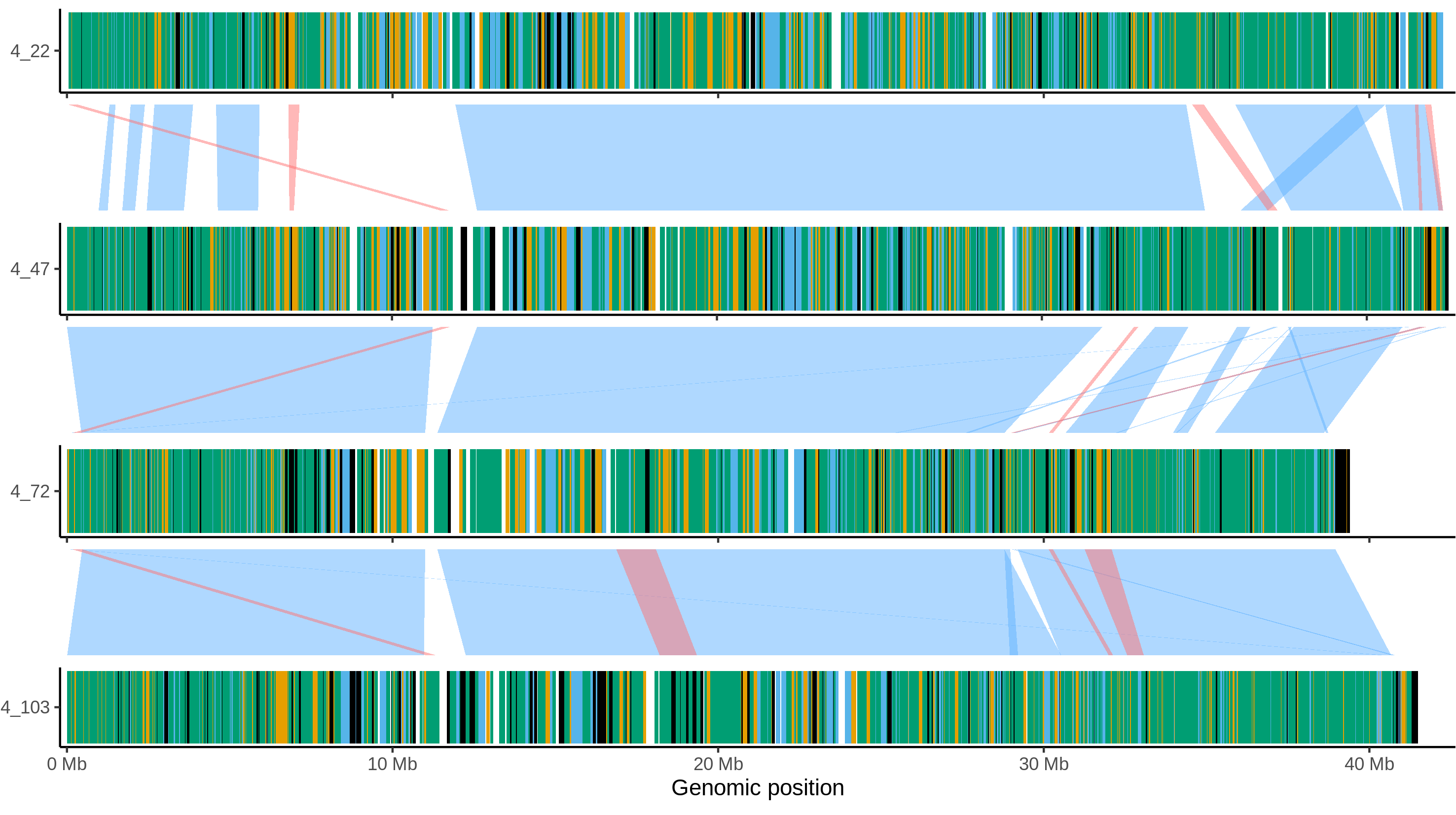

Supplement: Supplementary file 3 — Supplement S3 Supplementary Data. [file PBI-23-874-s002.zip › Supplementary_data/sequence_visualization/Potato/Otava_chr_10.png]

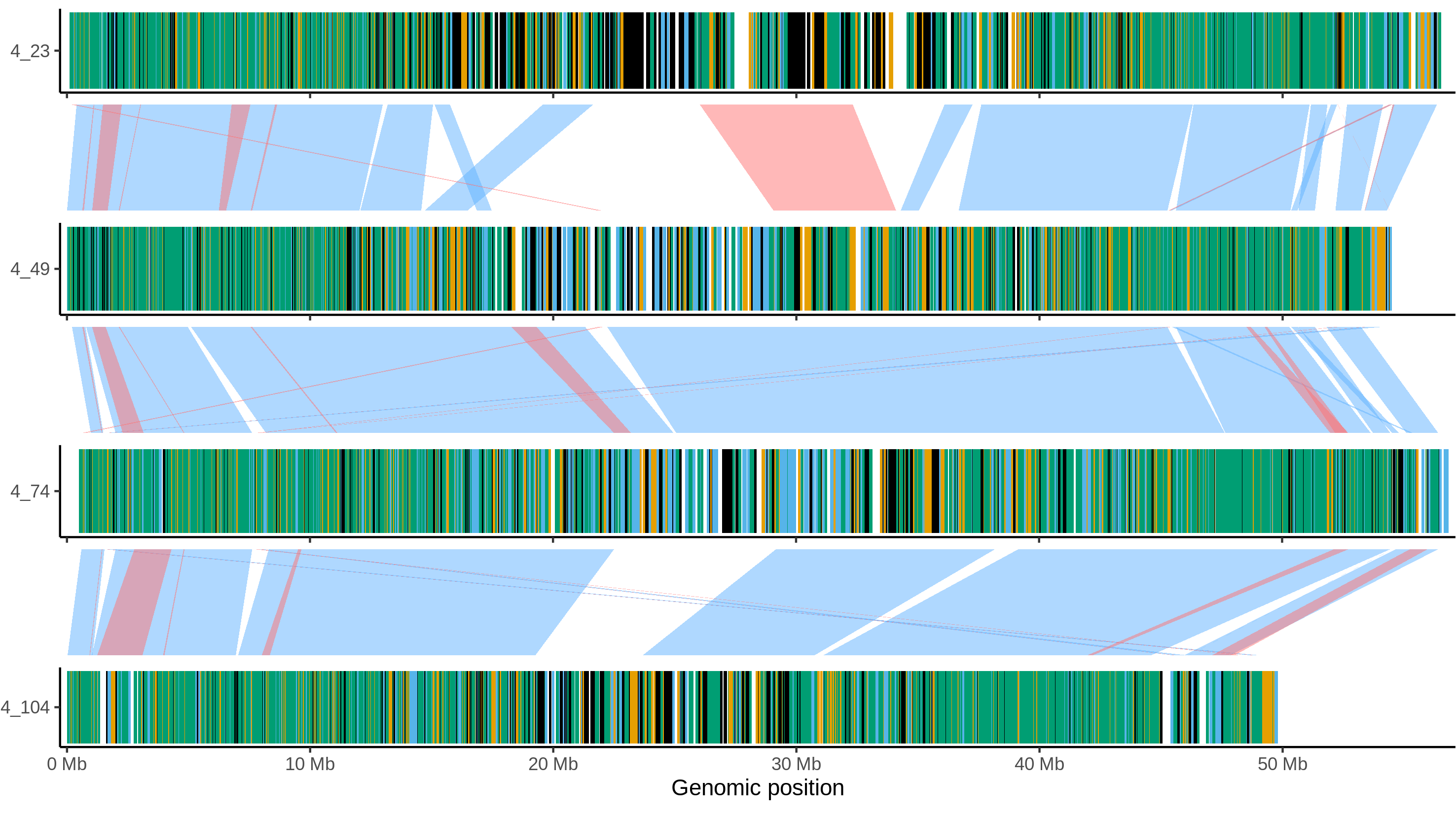

Supplement: Supplementary file 3 — Supplement S3 Supplementary Data. [file PBI-23-874-s002.zip › Supplementary_data/sequence_visualization/Potato/Otava_chr_11.png]

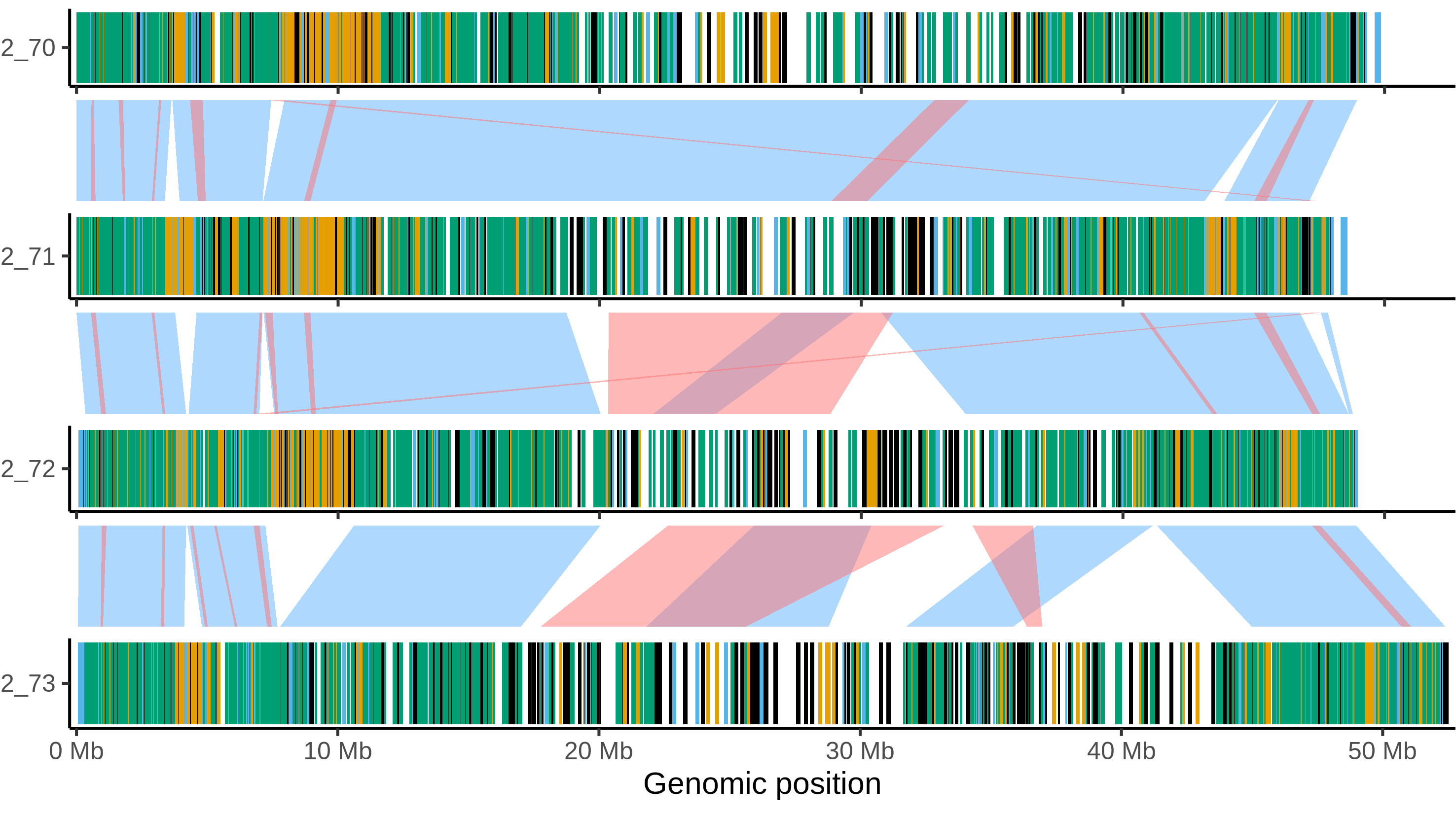

Supplement: Supplementary file 3 — Supplement S3 Supplementary Data. [file PBI-23-874-s002.zip › Supplementary_data/sequence_visualization/Potato/Atlantic_chr_5.png]

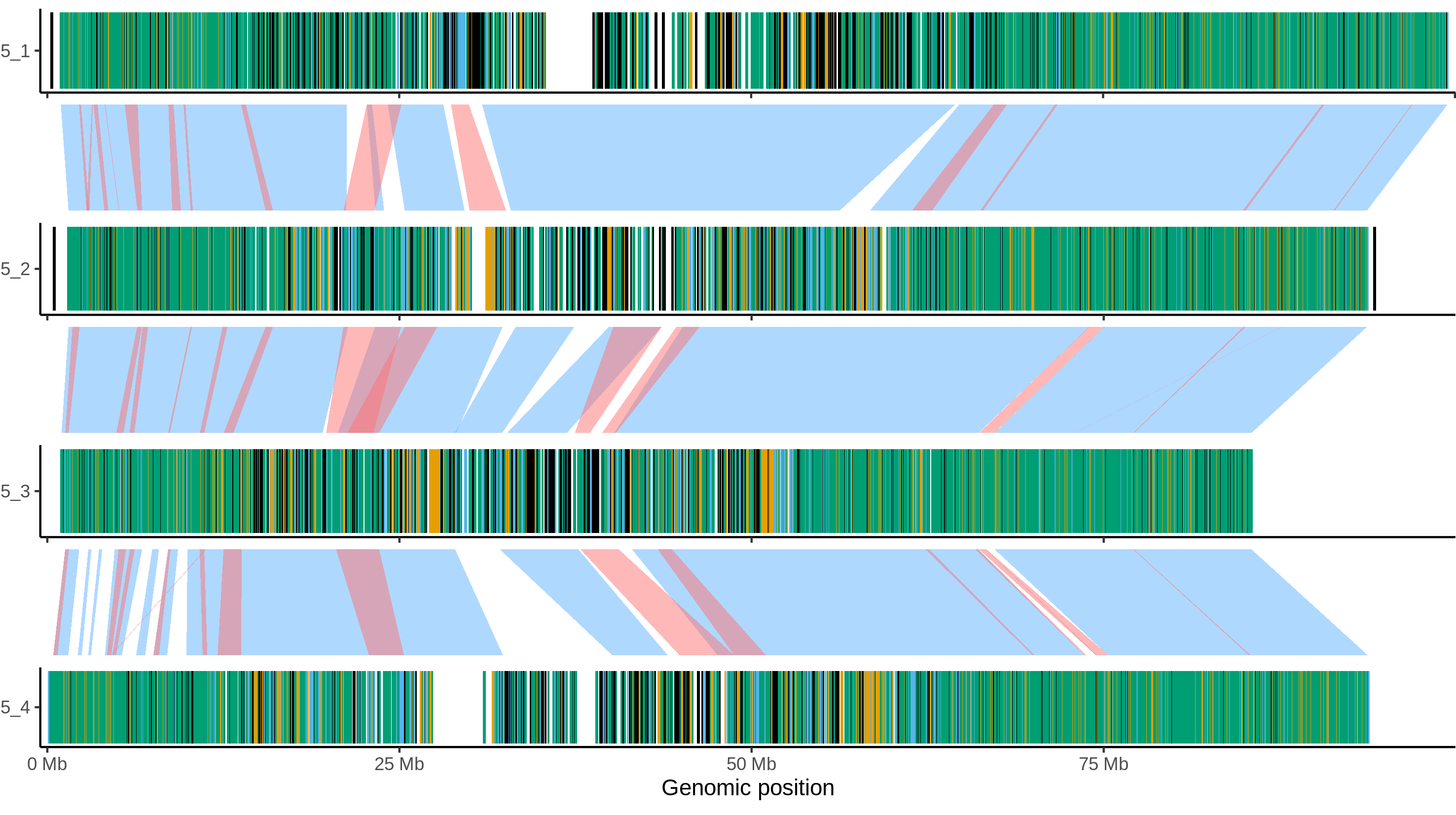

Supplement: Supplementary file 3 — Supplement S3 Supplementary Data. [file PBI-23-874-s002.zip › Supplementary_data/sequence_visualization/Potato/C88_chr_1.png]

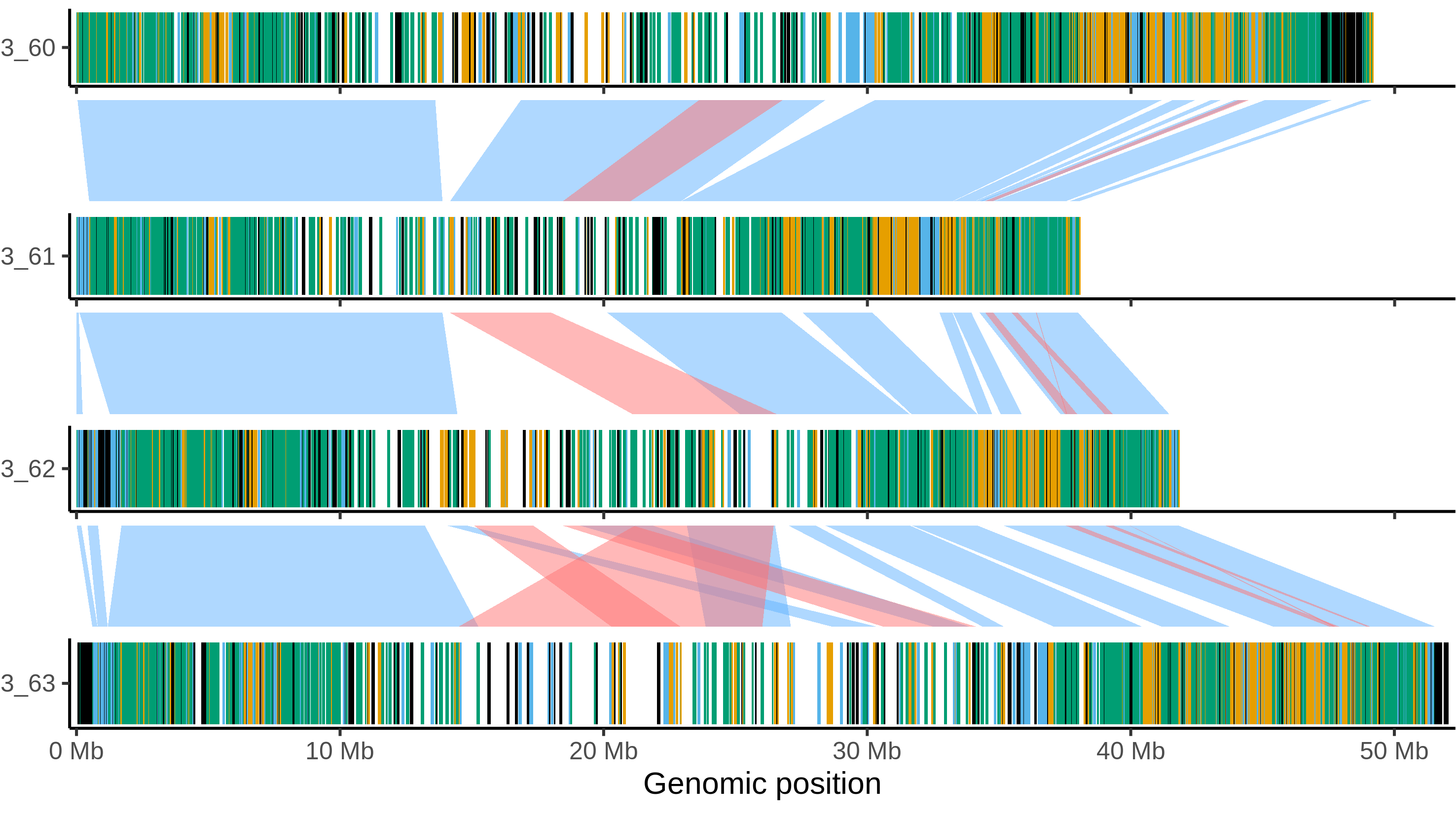

Supplement: Supplementary file 3 — Supplement S3 Supplementary Data. [file PBI-23-874-s002.zip › Supplementary_data/sequence_visualization/Potato/Castle_russet_chr_7.png]

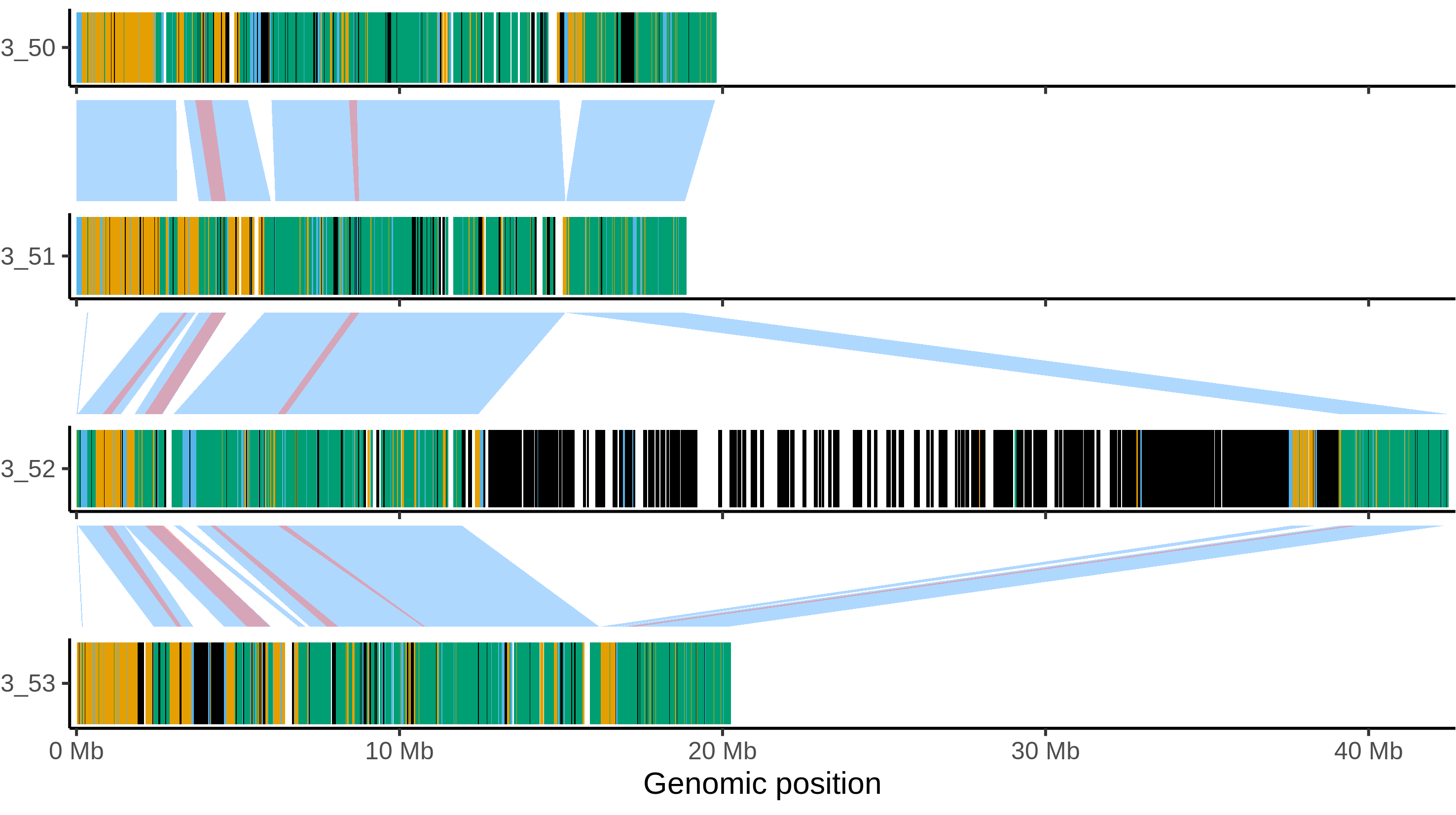

Supplement: Supplementary file 3 — Supplement S3 Supplementary Data. [file PBI-23-874-s002.zip › Supplementary_data/sequence_visualization/Potato/Castle_russet_chr_5.png]

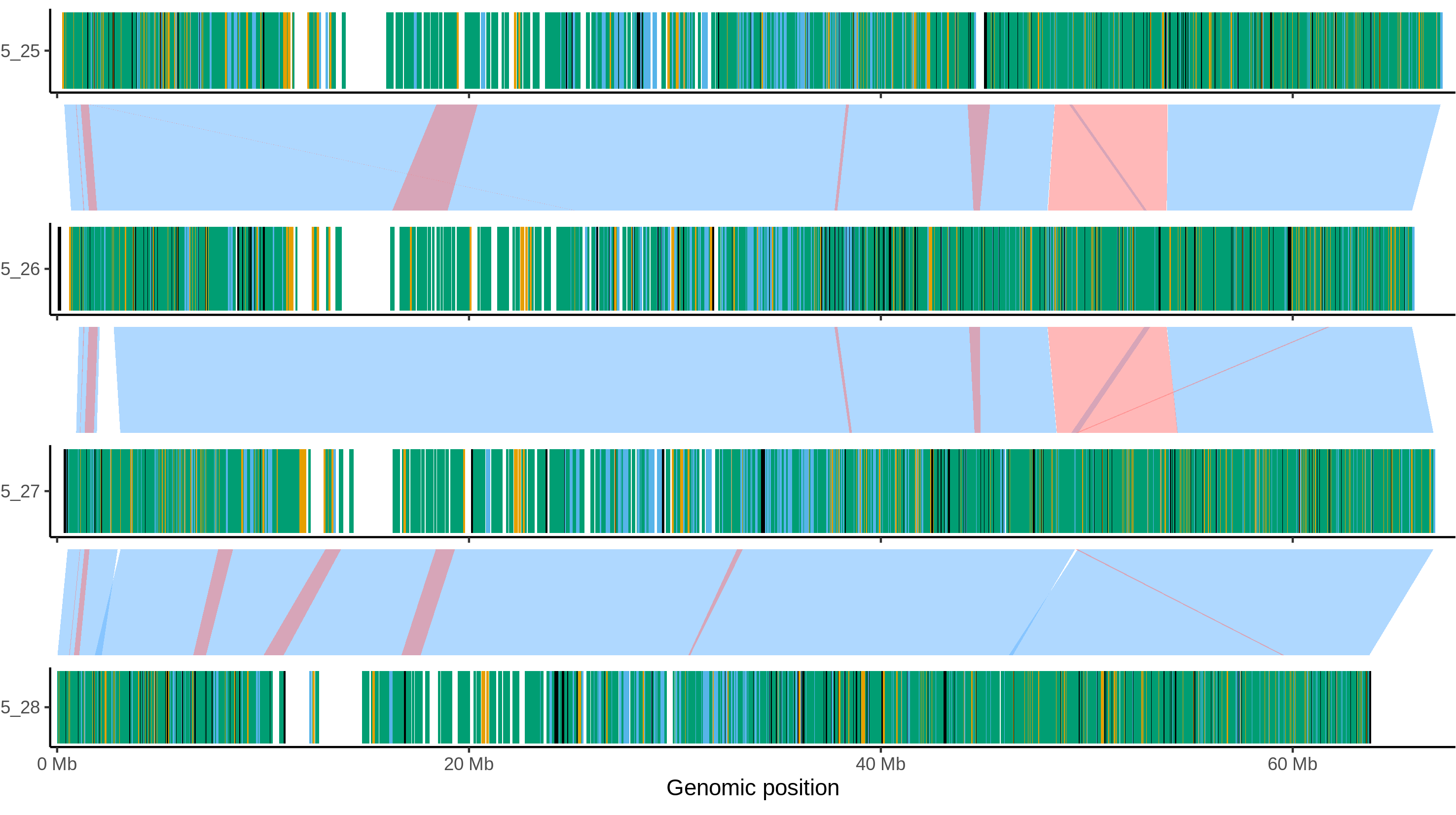

Supplement: Supplementary file 3 — Supplement S3 Supplementary Data. [file PBI-23-874-s002.zip › Supplementary_data/sequence_visualization/Potato/C88_chr_3.png]

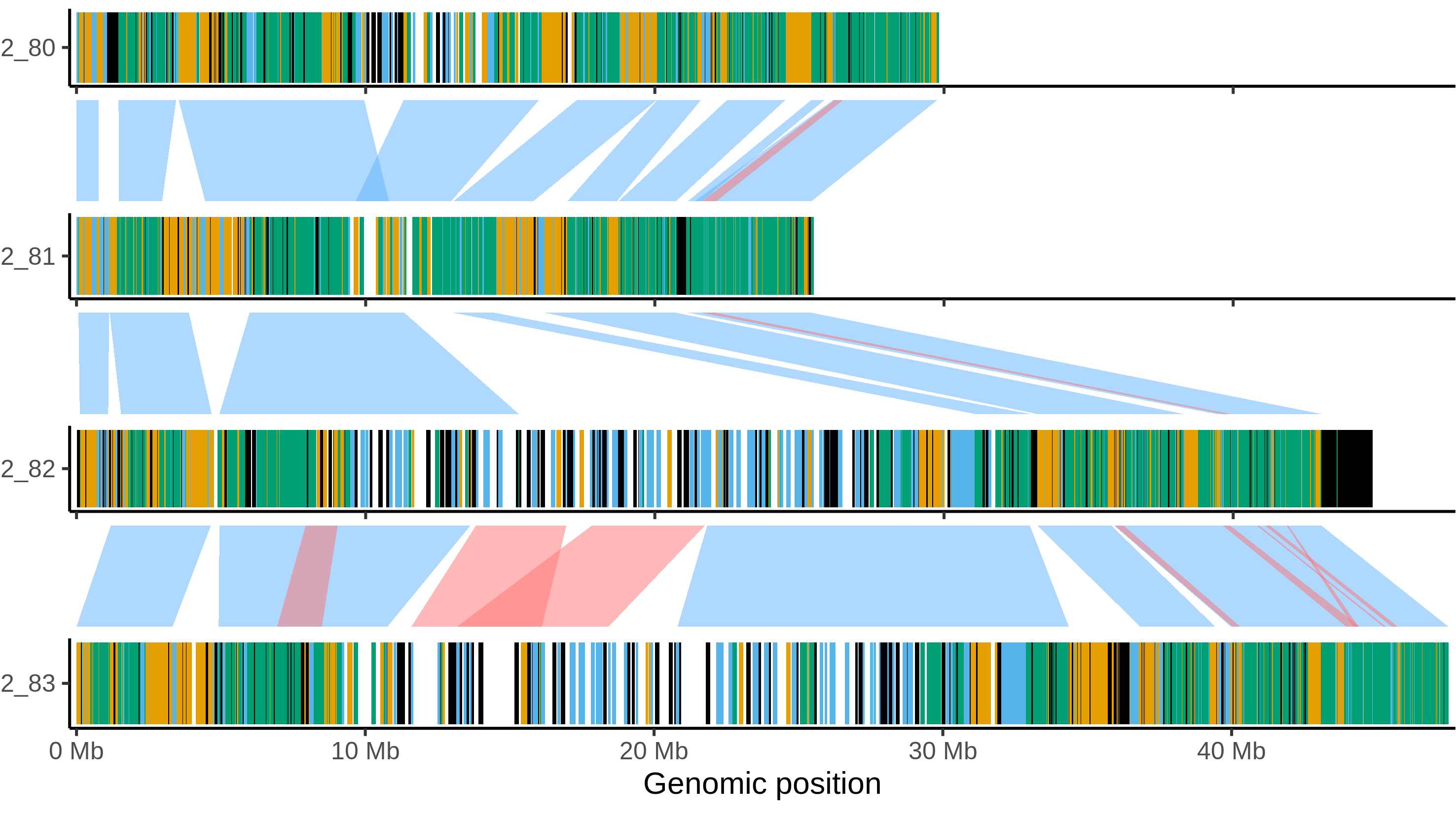

Supplement: Supplementary file 3 — Supplement S3 Supplementary Data. [file PBI-23-874-s002.zip › Supplementary_data/sequence_visualization/Potato/Atlantic_chr_7.png]

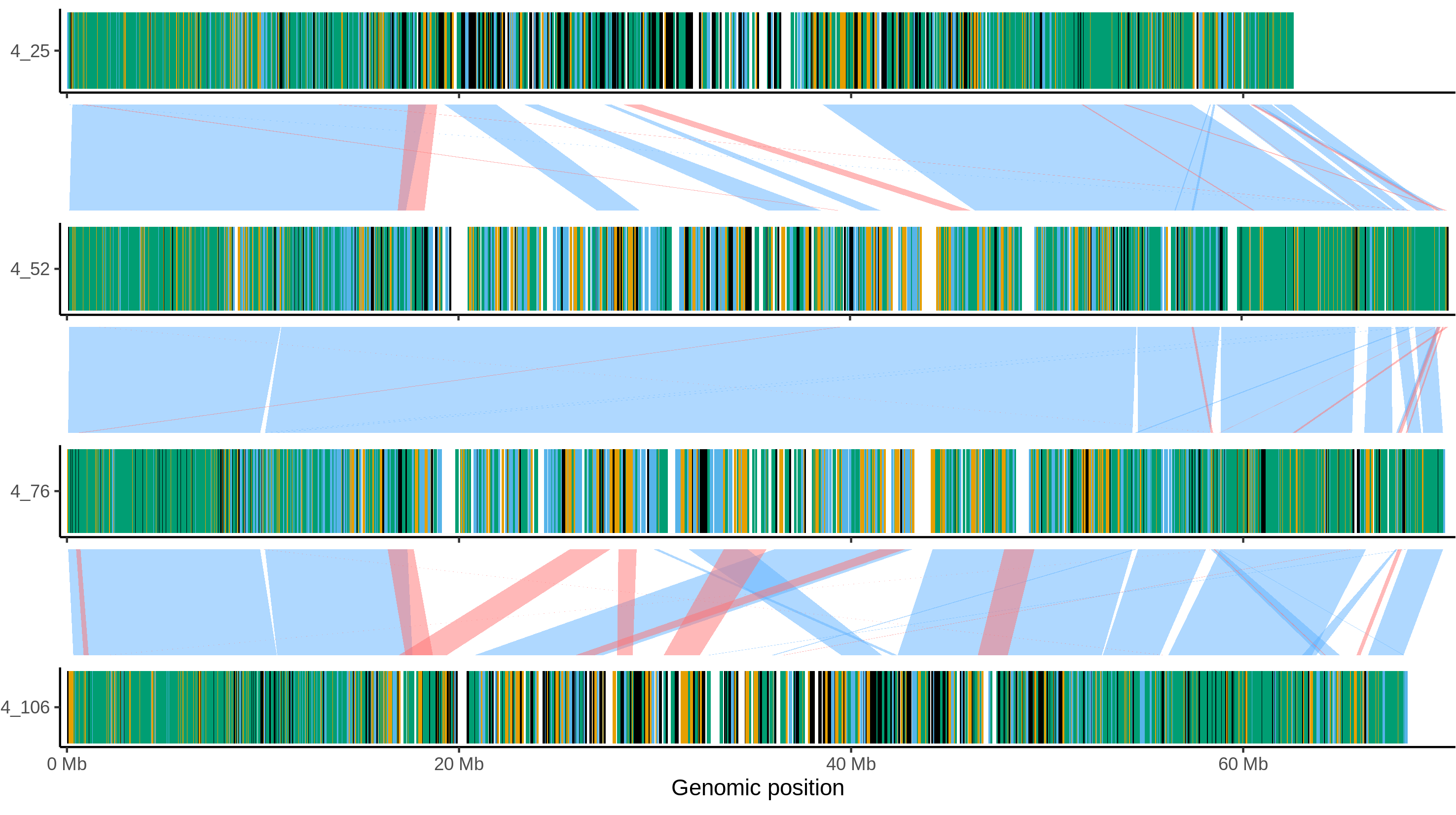

Supplement: Supplementary file 3 — Supplement S3 Supplementary Data. [file PBI-23-874-s002.zip › Supplementary_data/sequence_visualization/Potato/Otava_chr_12.png]

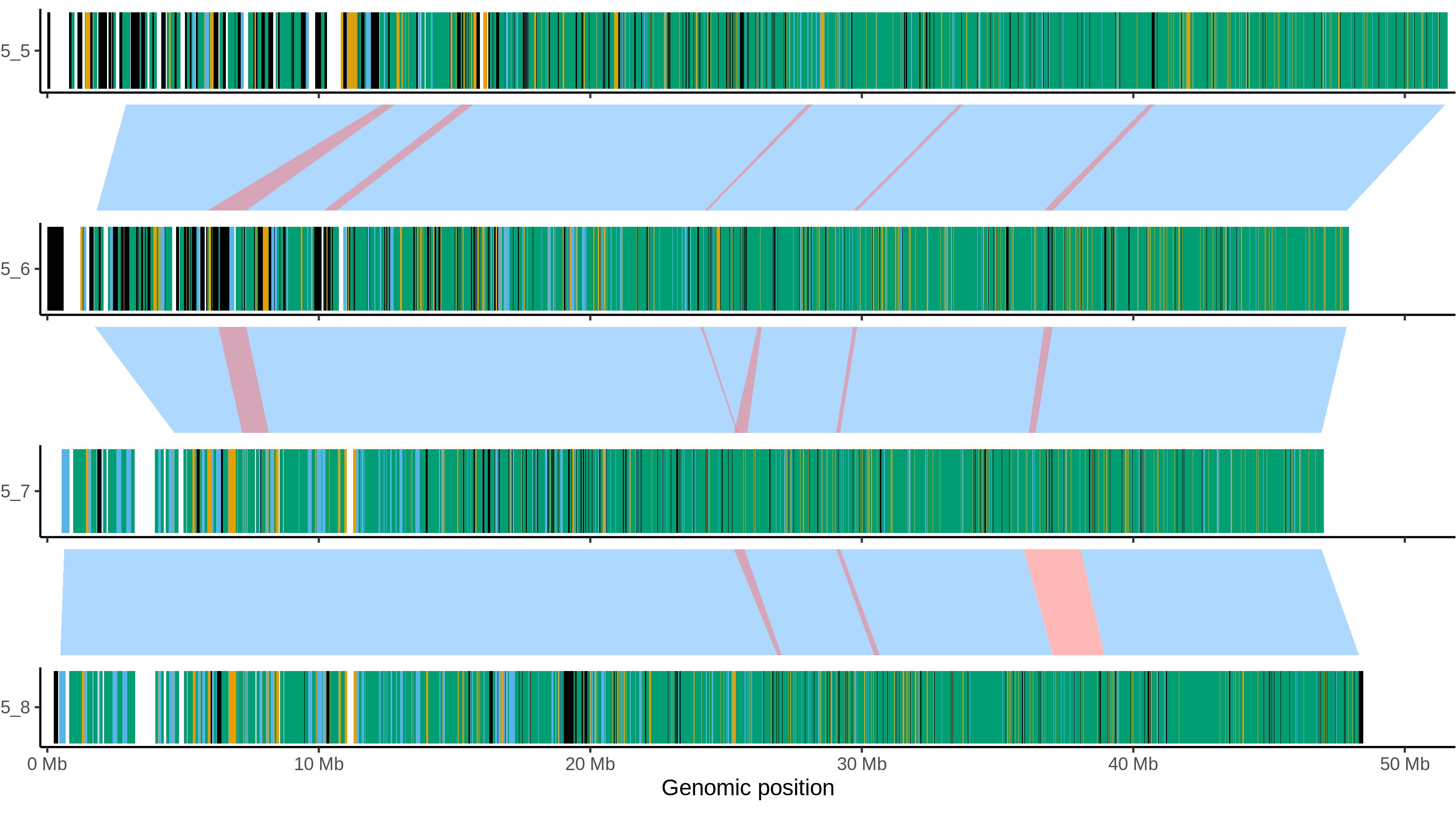

Supplement: Supplementary file 3 — Supplement S3 Supplementary Data. [file PBI-23-874-s002.zip › Supplementary_data/sequence_visualization/Potato/C88_chr_2.png]

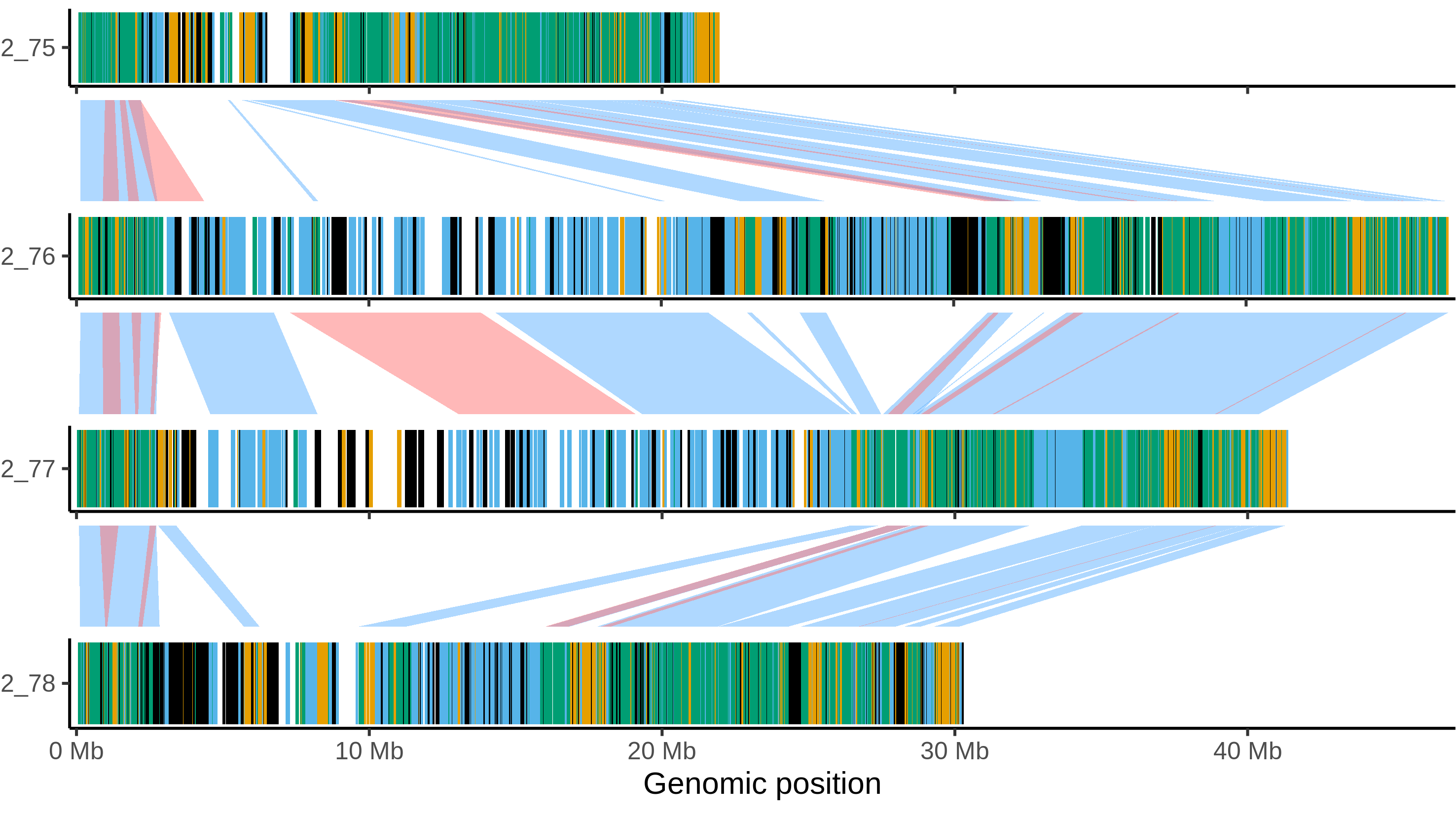

Supplement: Supplementary file 3 — Supplement S3 Supplementary Data. [file PBI-23-874-s002.zip › Supplementary_data/sequence_visualization/Potato/Atlantic_chr_6.png]

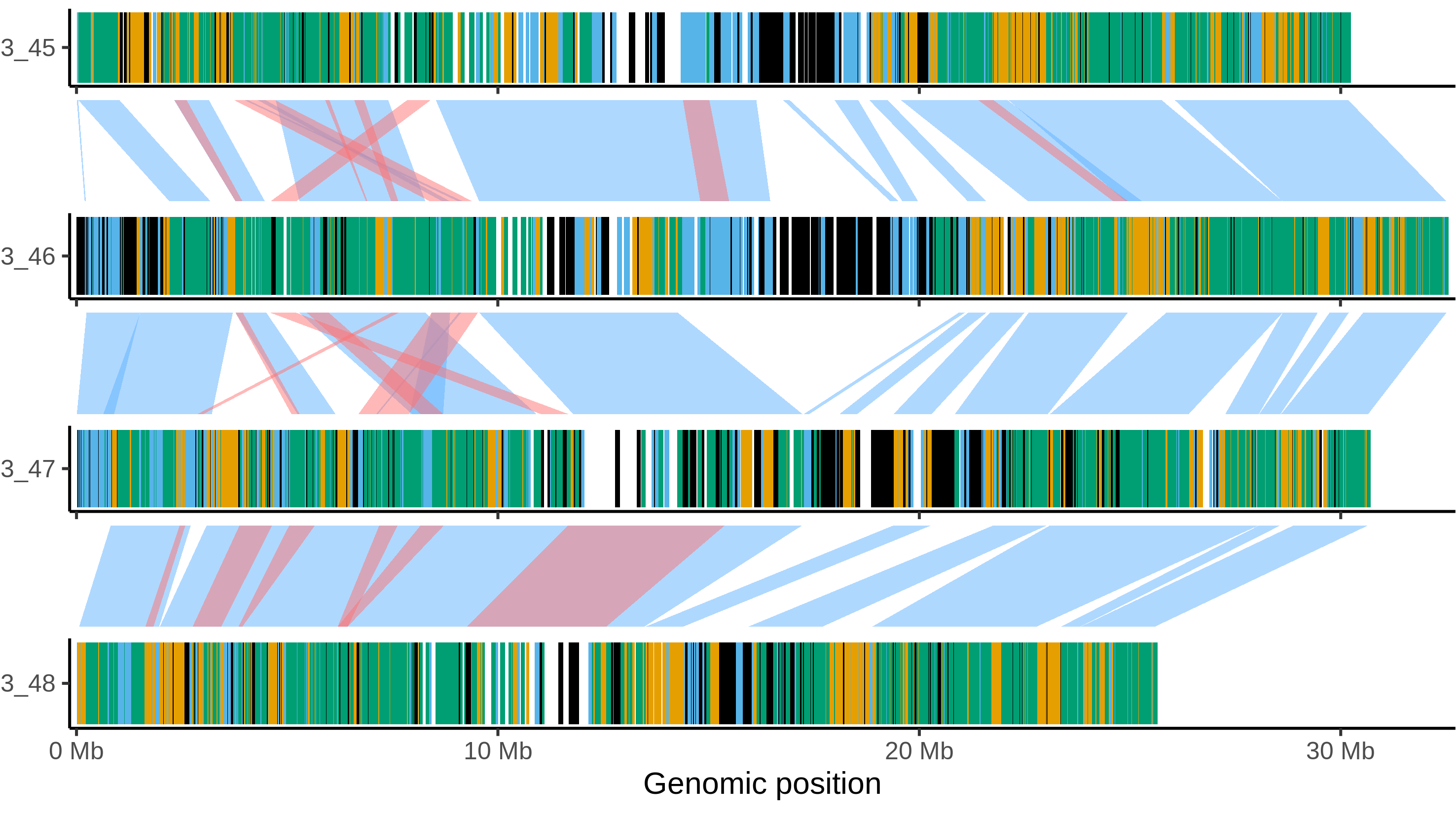

Supplement: Supplementary file 3 — Supplement S3 Supplementary Data. [file PBI-23-874-s002.zip › Supplementary_data/sequence_visualization/Potato/Castle_russet_chr_4.png]

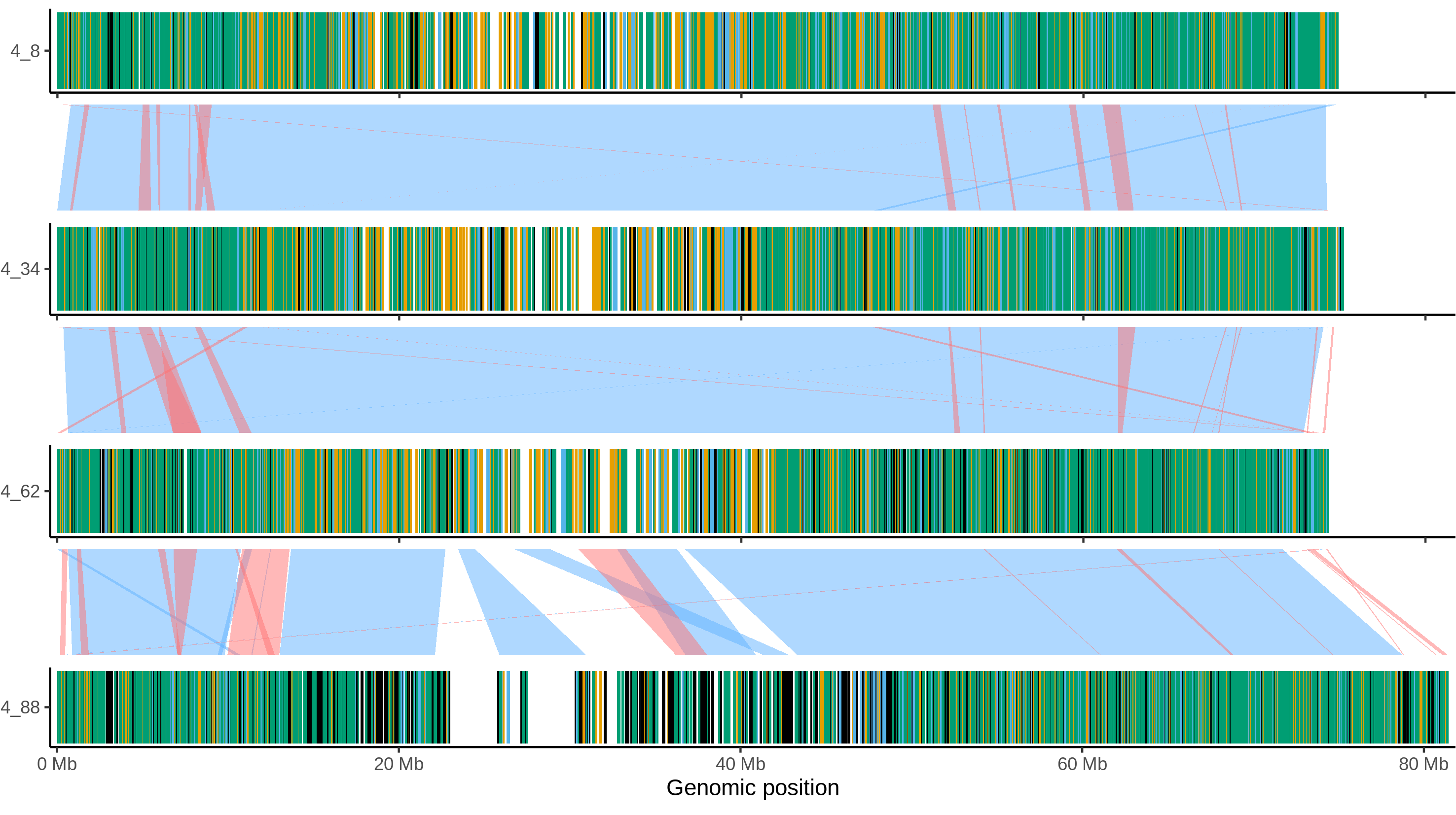

Supplement: Supplementary file 3 — Supplement S3 Supplementary Data. [file PBI-23-874-s002.zip › Supplementary_data/sequence_visualization/Potato/Otava_chr_4.png]

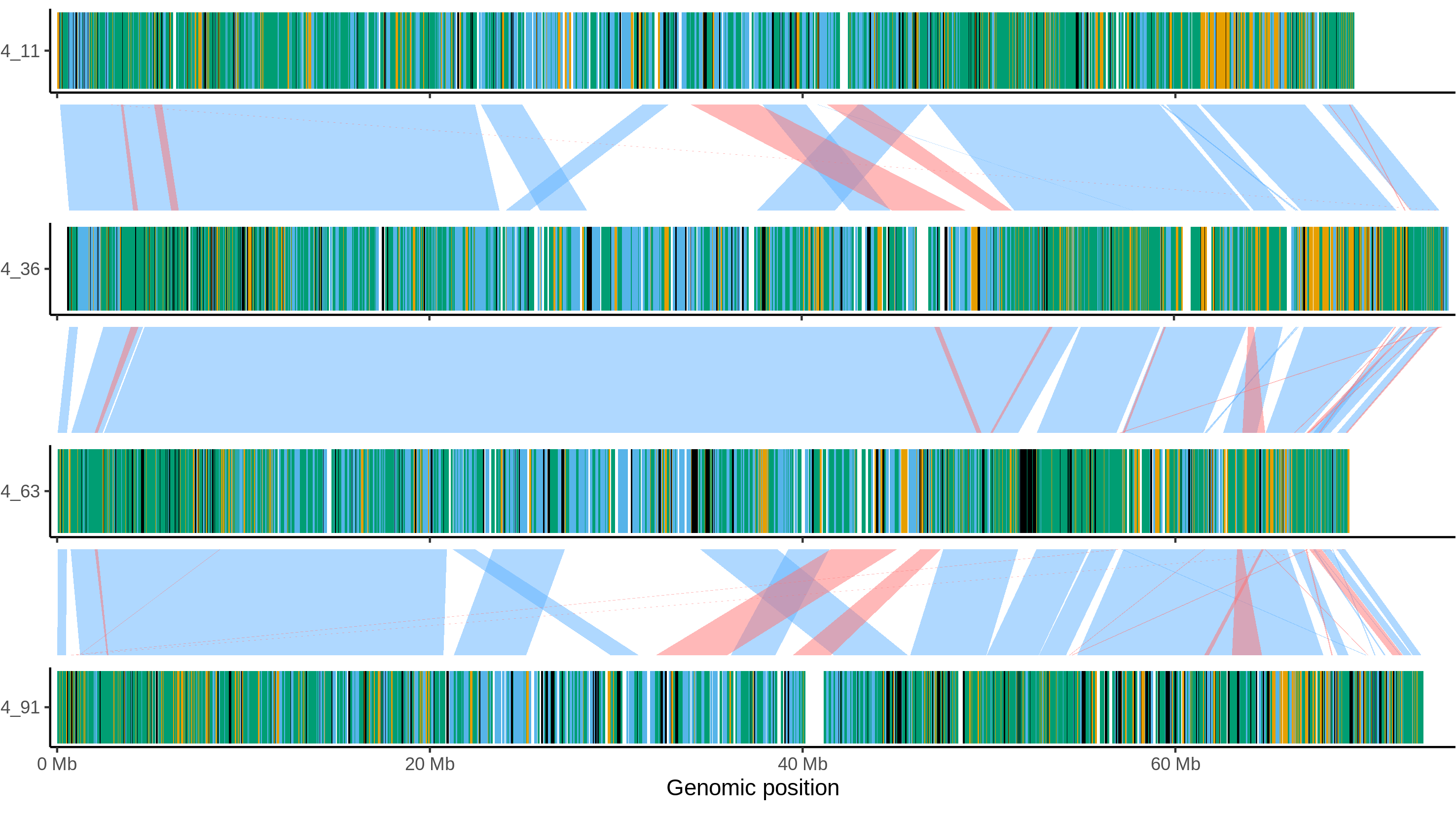

Supplement: Supplementary file 3 — Supplement S3 Supplementary Data. [file PBI-23-874-s002.zip › Supplementary_data/sequence_visualization/Potato/Otava_chr_5.png]

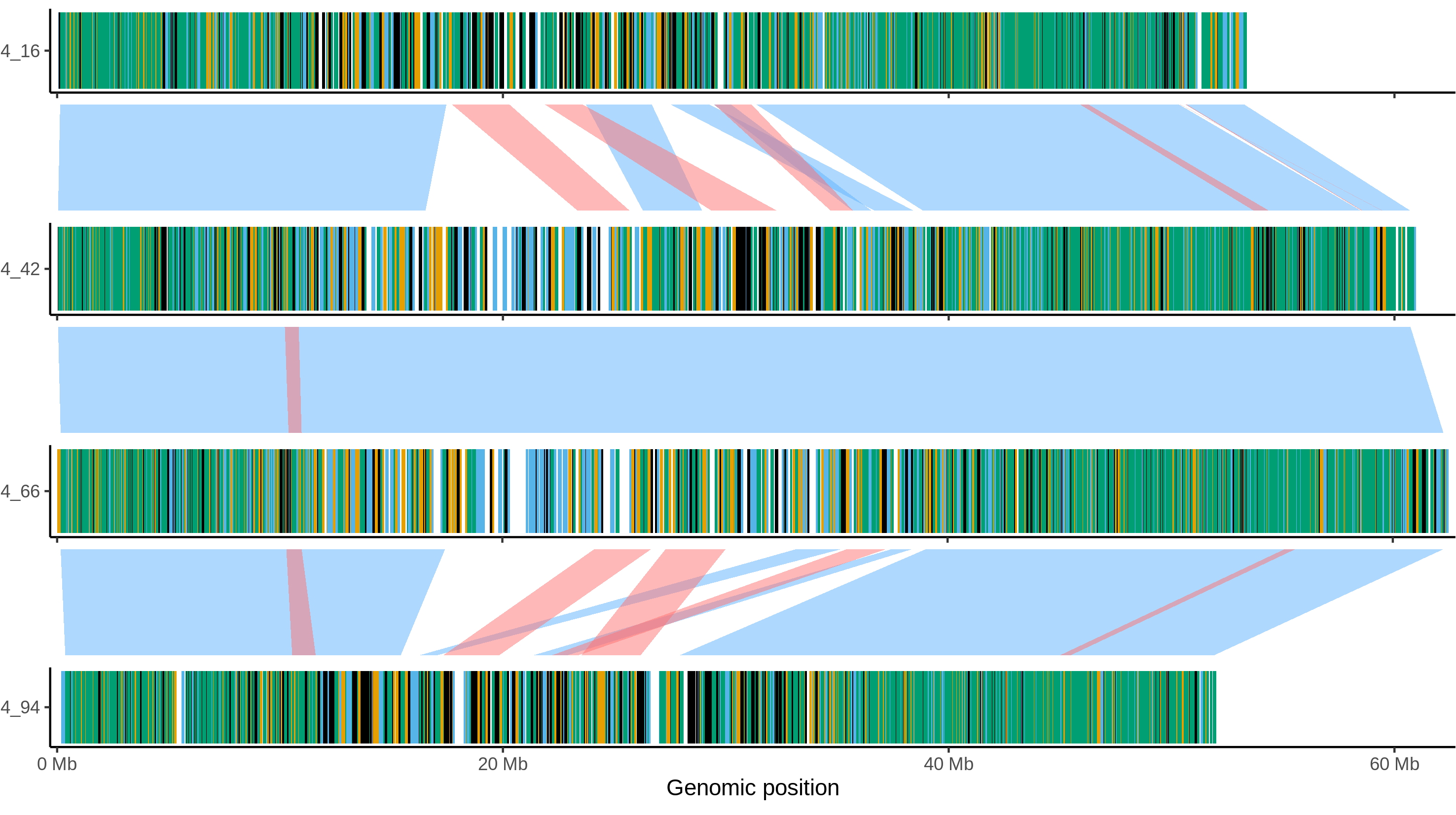

Supplement: Supplementary file 3 — Supplement S3 Supplementary Data. [file PBI-23-874-s002.zip › Supplementary_data/sequence_visualization/Potato/Otava_chr_7.png]

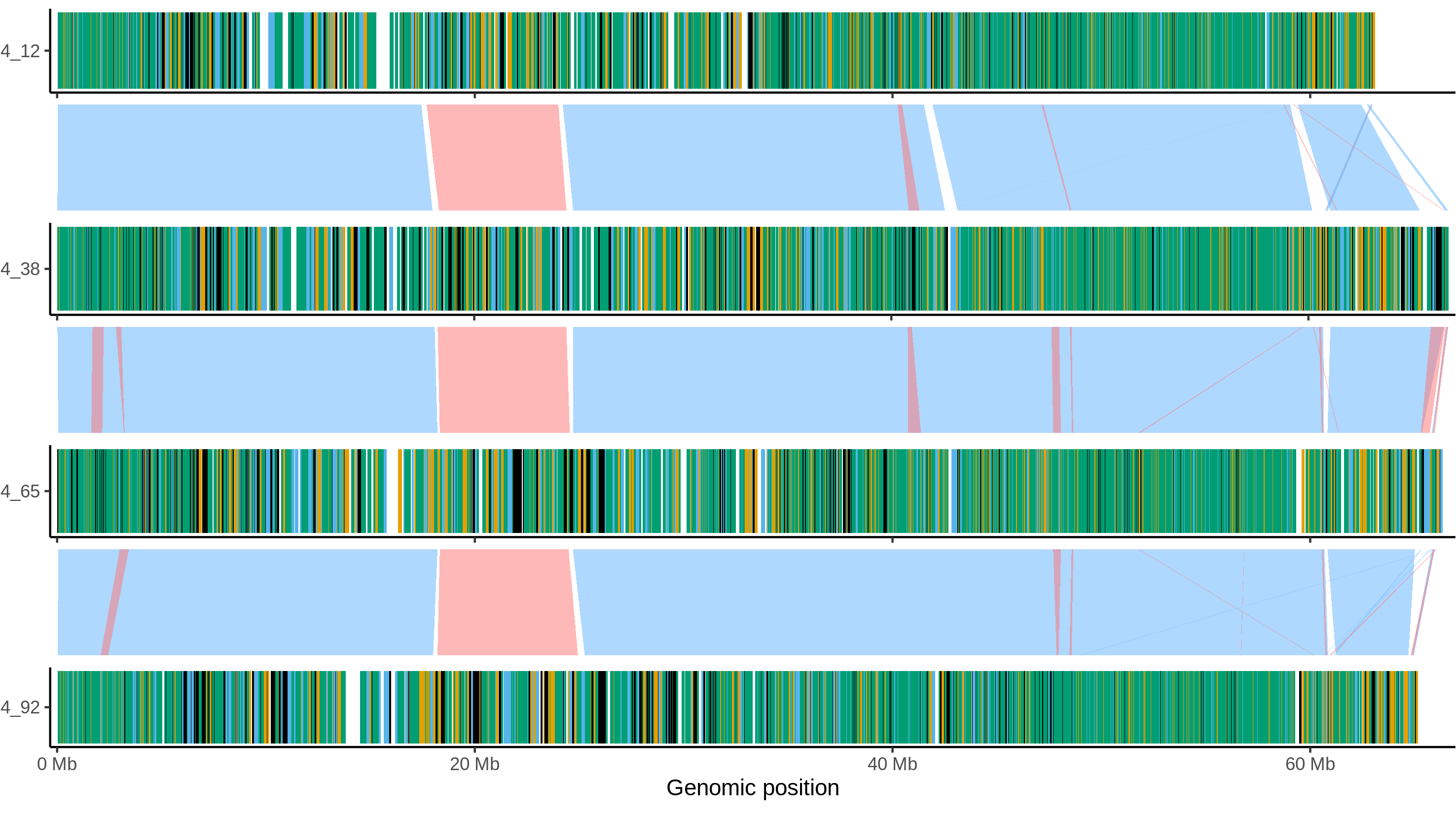

Supplement: Supplementary file 3 — Supplement S3 Supplementary Data. [file PBI-23-874-s002.zip › Supplementary_data/sequence_visualization/Potato/Otava_chr_6.png]

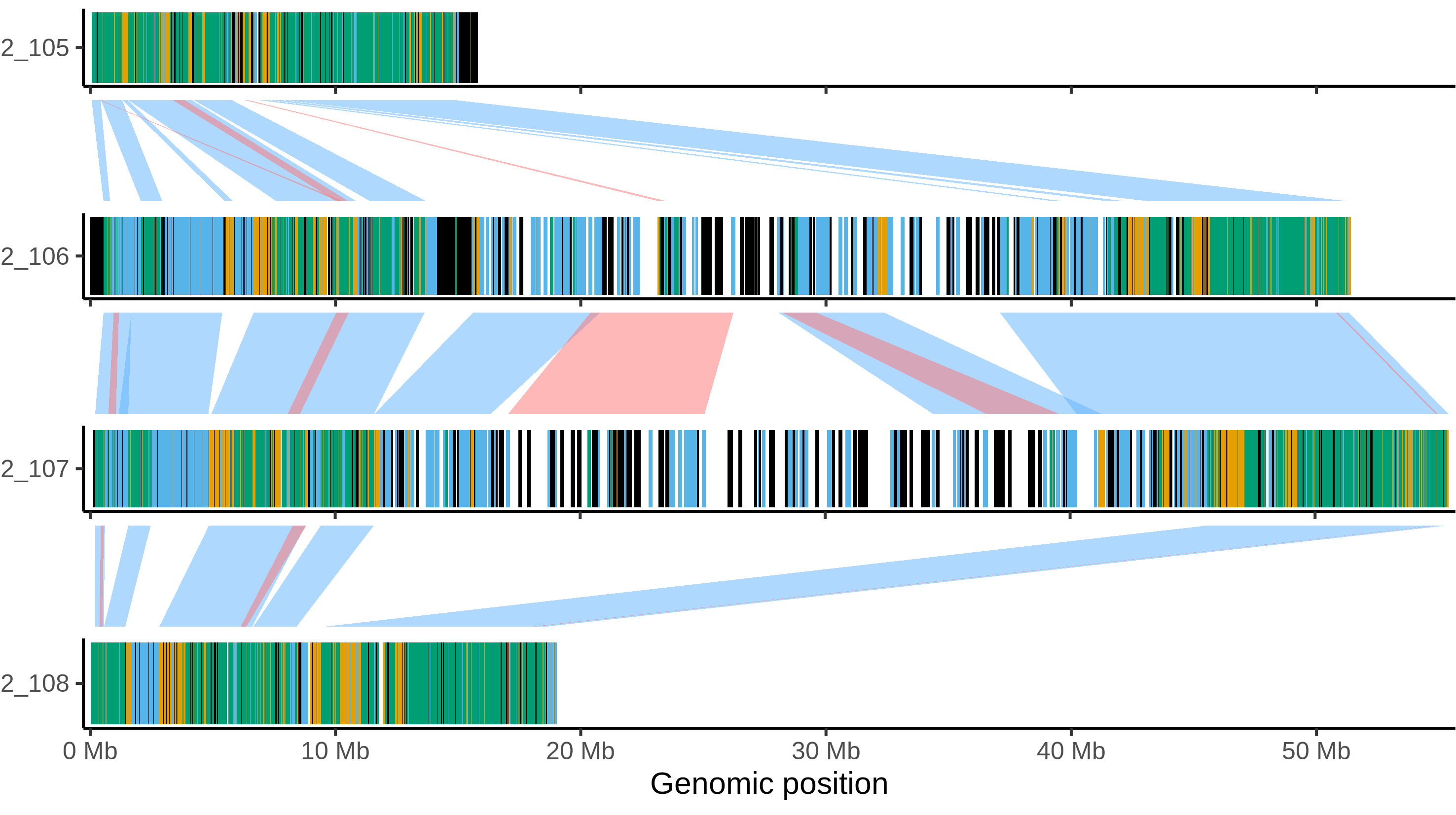

Supplement: Supplementary file 3 — Supplement S3 Supplementary Data. [file PBI-23-874-s002.zip › Supplementary_data/sequence_visualization/Potato/Atlantic_chr_12.png]

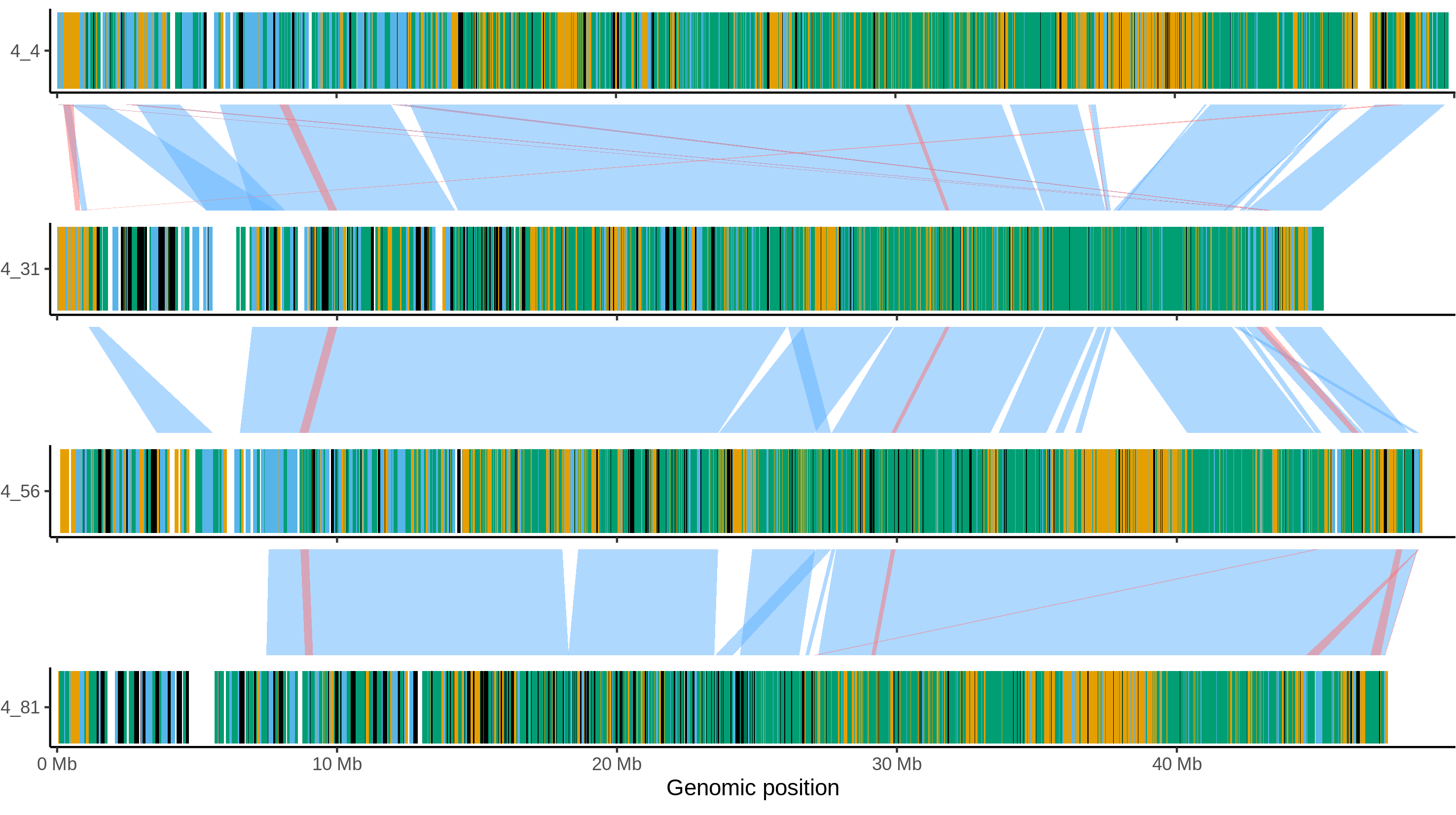

Supplement: Supplementary file 3 — Supplement S3 Supplementary Data. [file PBI-23-874-s002.zip › Supplementary_data/sequence_visualization/Potato/Otava_chr_2.png]

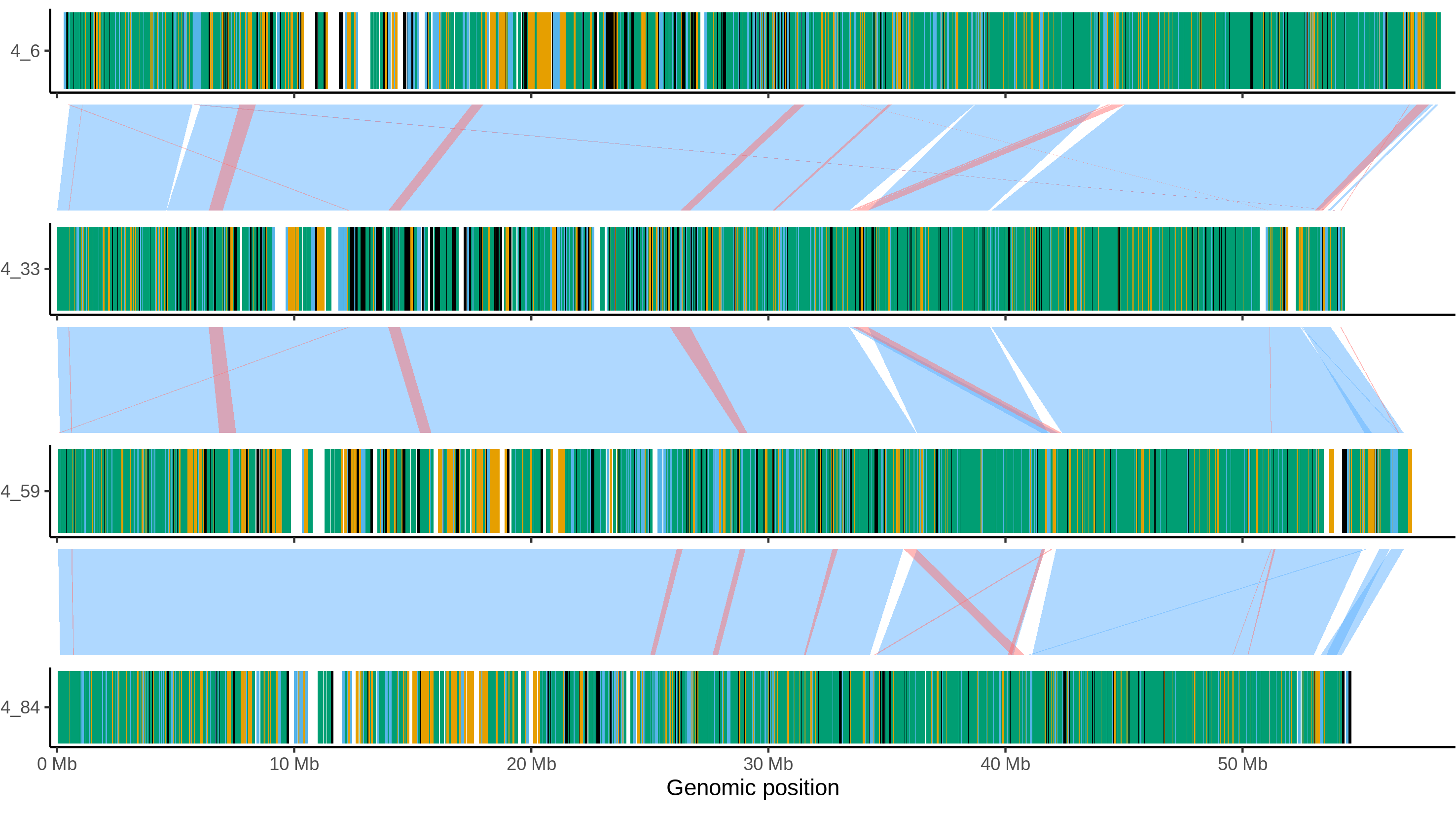

Supplement: Supplementary file 3 — Supplement S3 Supplementary Data. [file PBI-23-874-s002.zip › Supplementary_data/sequence_visualization/Potato/Otava_chr_3.png]

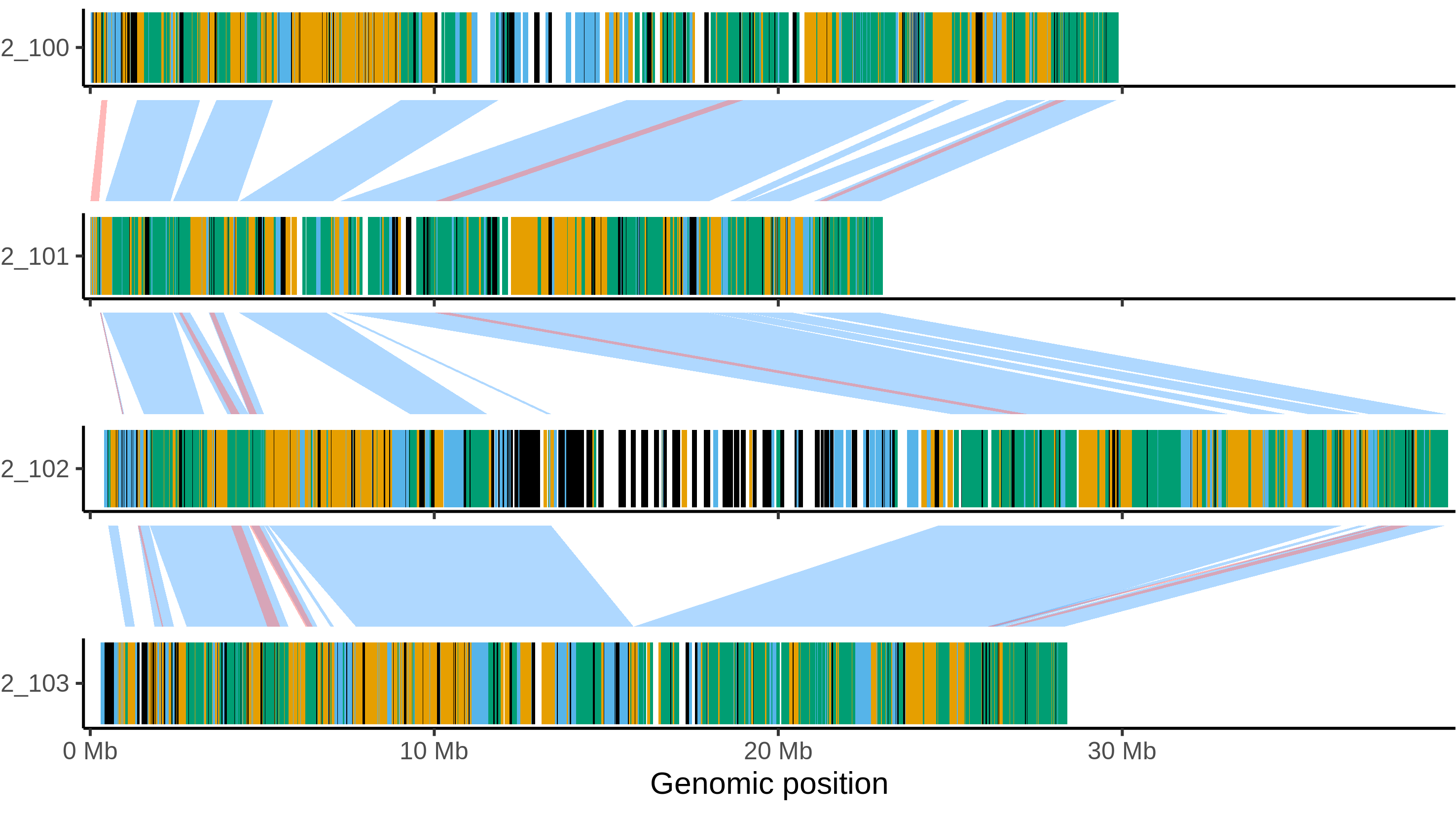

Supplement: Supplementary file 3 — Supplement S3 Supplementary Data. [file PBI-23-874-s002.zip › Supplementary_data/sequence_visualization/Potato/Atlantic_chr_11.png]

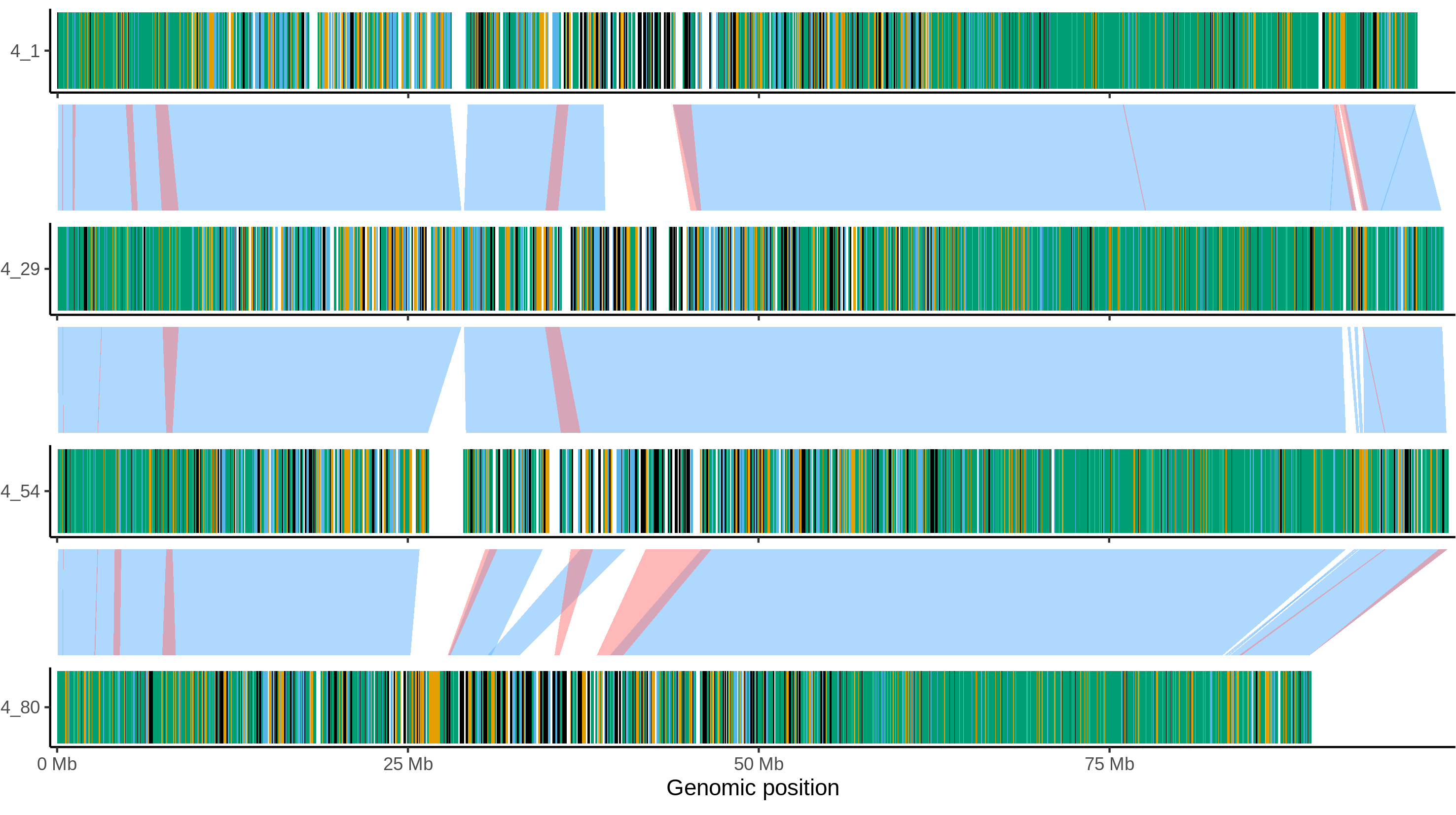

Supplement: Supplementary file 3 — Supplement S3 Supplementary Data. [file PBI-23-874-s002.zip › Supplementary_data/sequence_visualization/Potato/Otava_chr_1.png]

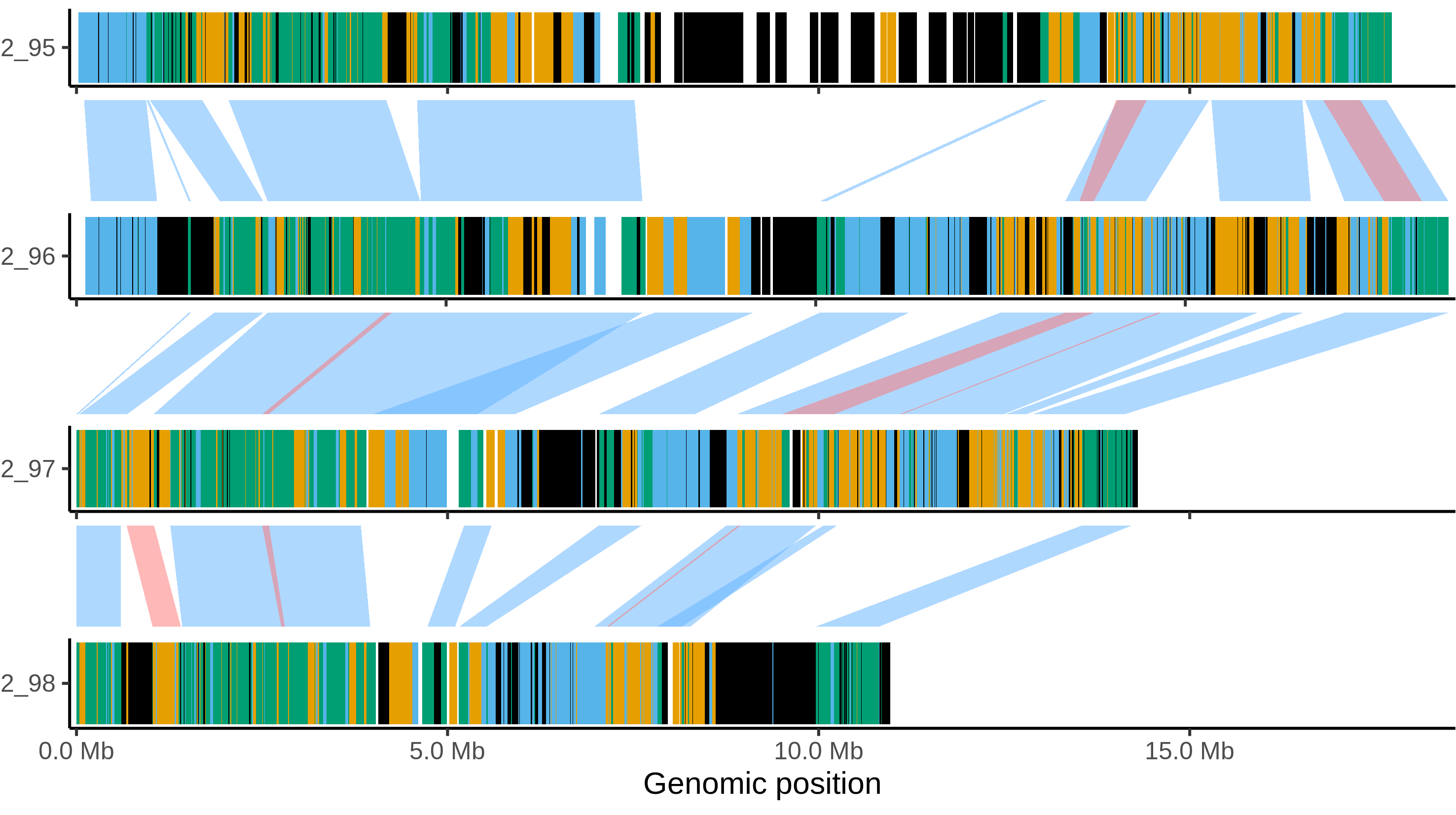

Supplement: Supplementary file 3 — Supplement S3 Supplementary Data. [file PBI-23-874-s002.zip › Supplementary_data/sequence_visualization/Potato/Atlantic_chr_10.png]

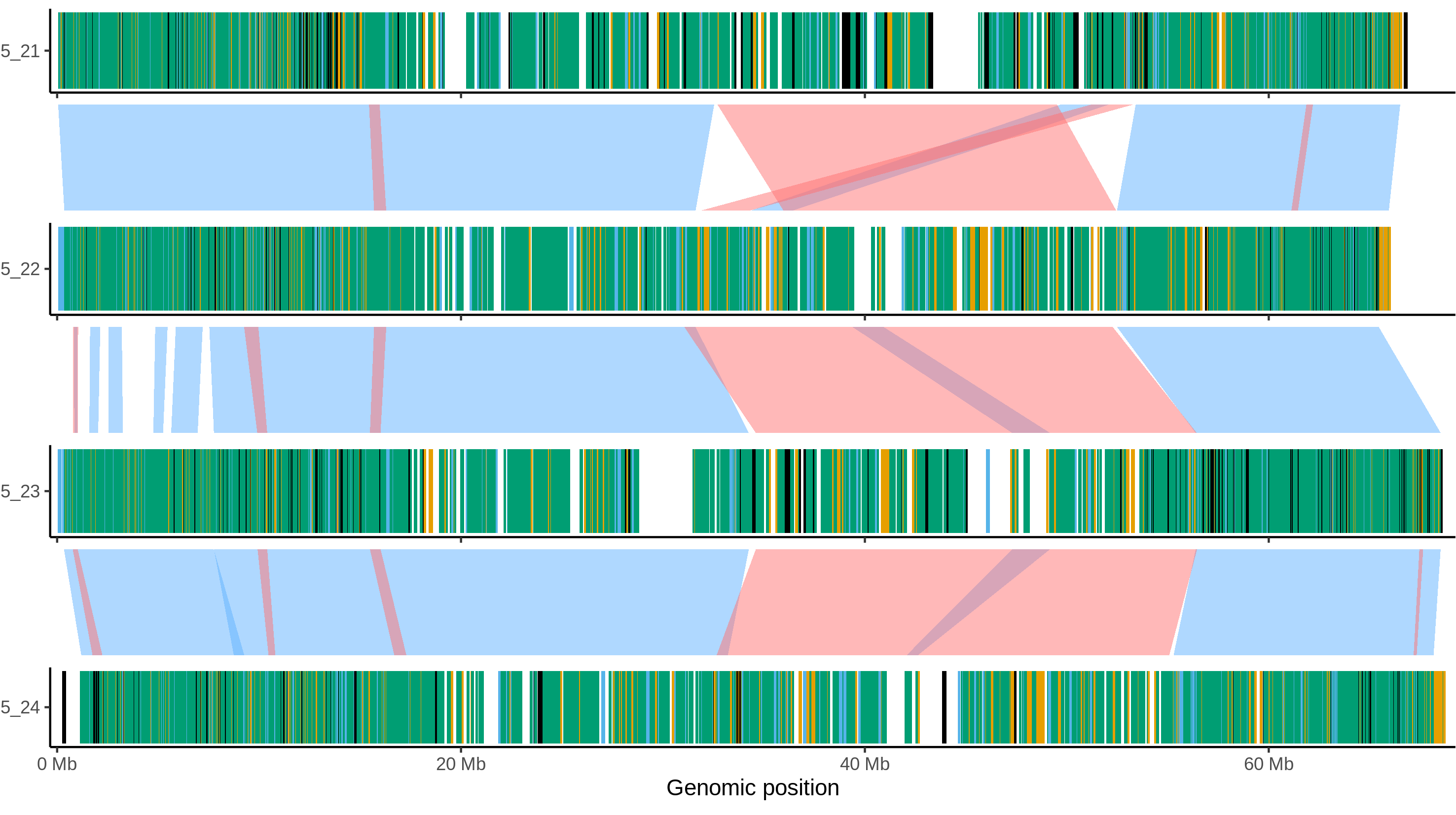

Supplement: Supplementary file 3 — Supplement S3 Supplementary Data. [file PBI-23-874-s002.zip › Supplementary_data/sequence_visualization/Potato/C88_chr_12.png]

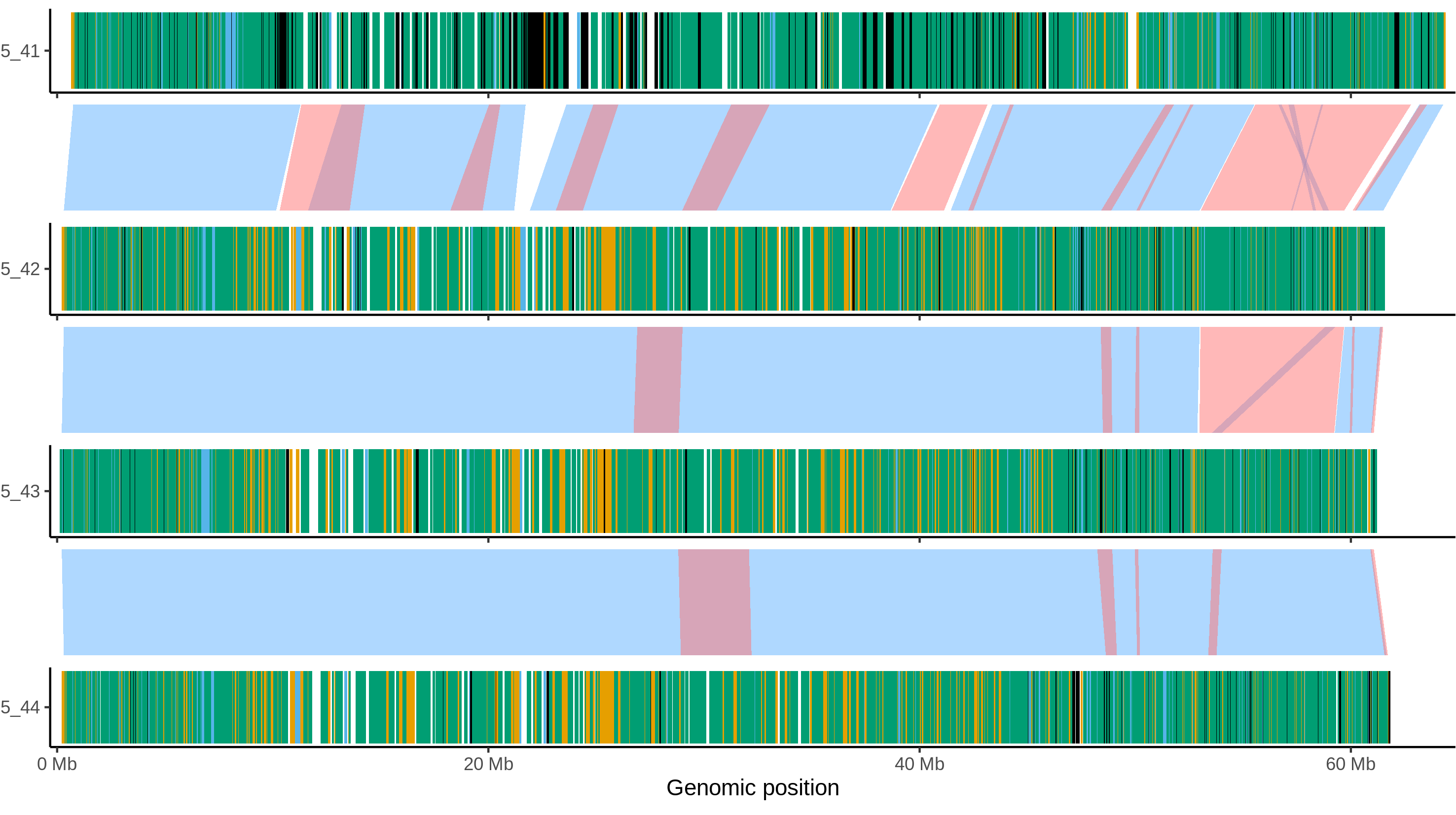

Supplement: Supplementary file 3 — Supplement S3 Supplementary Data. [file PBI-23-874-s002.zip › Supplementary_data/sequence_visualization/Potato/C88_chr_10.png]

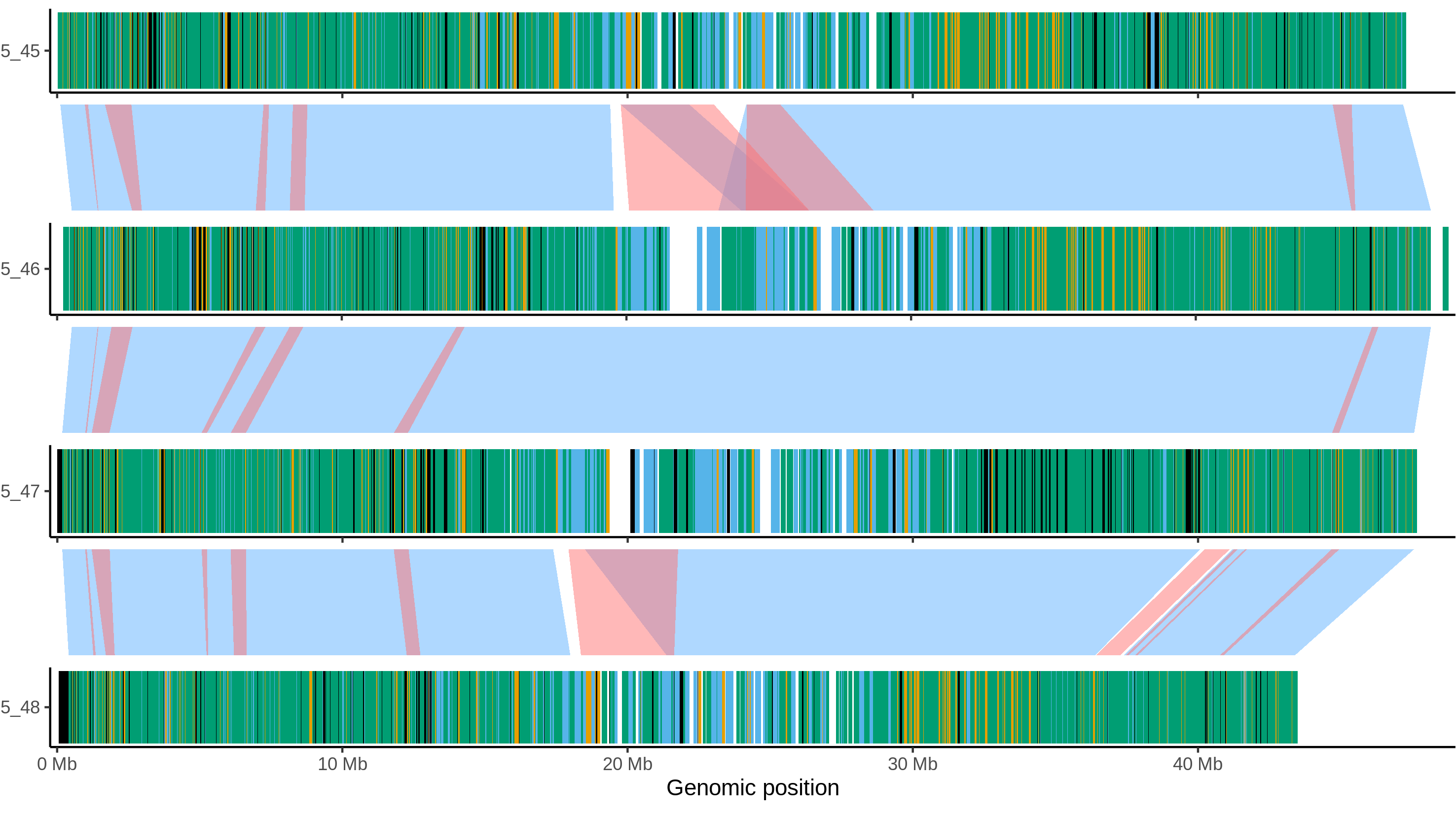

Supplement: Supplementary file 3 — Supplement S3 Supplementary Data. [file PBI-23-874-s002.zip › Supplementary_data/sequence_visualization/Potato/C88_chr_11.png]

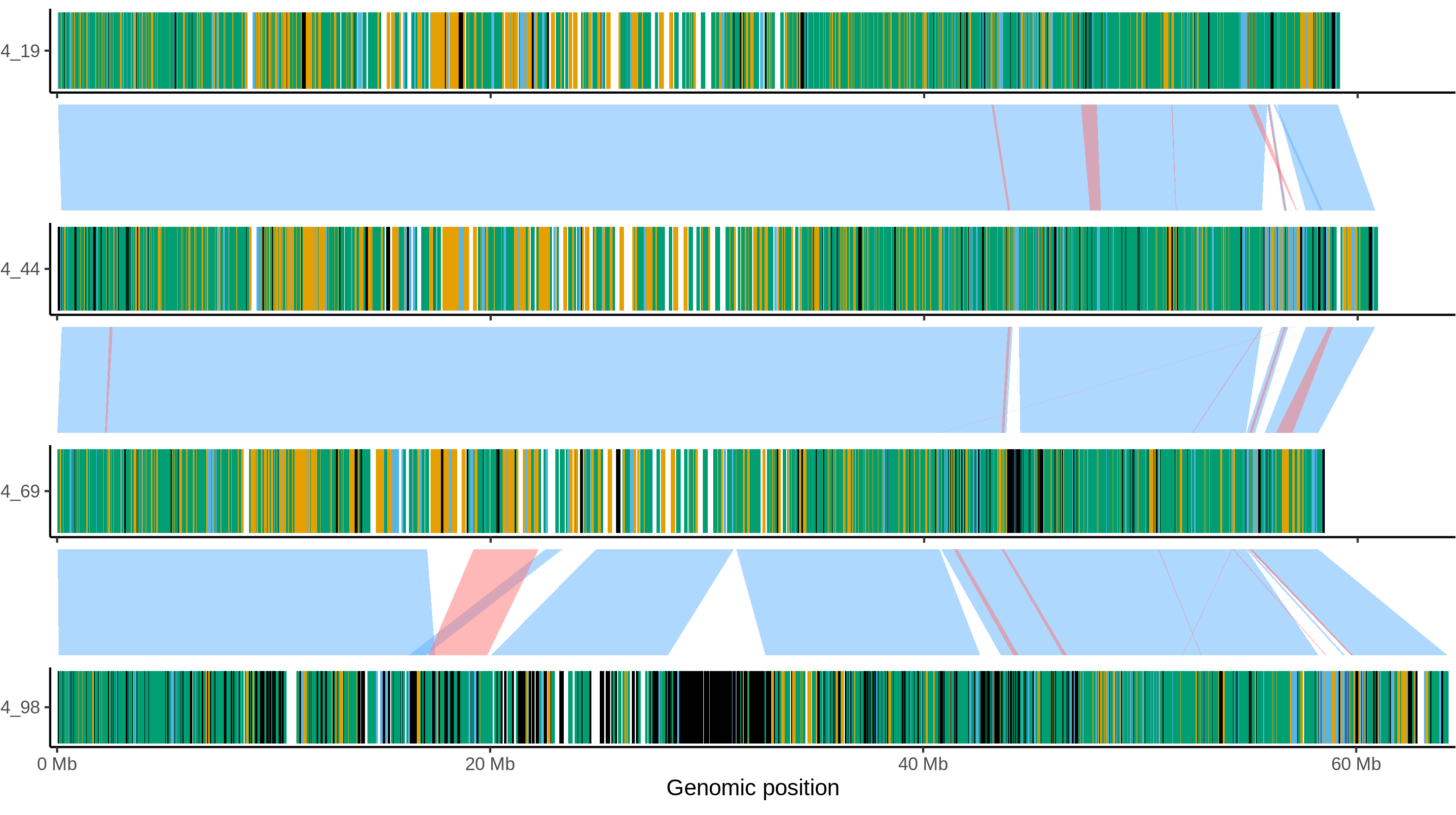

Supplement: Supplementary file 3 — Supplement S3 Supplementary Data. [file PBI-23-874-s002.zip › Supplementary_data/sequence_visualization/Potato/Otava_chr_8.png]

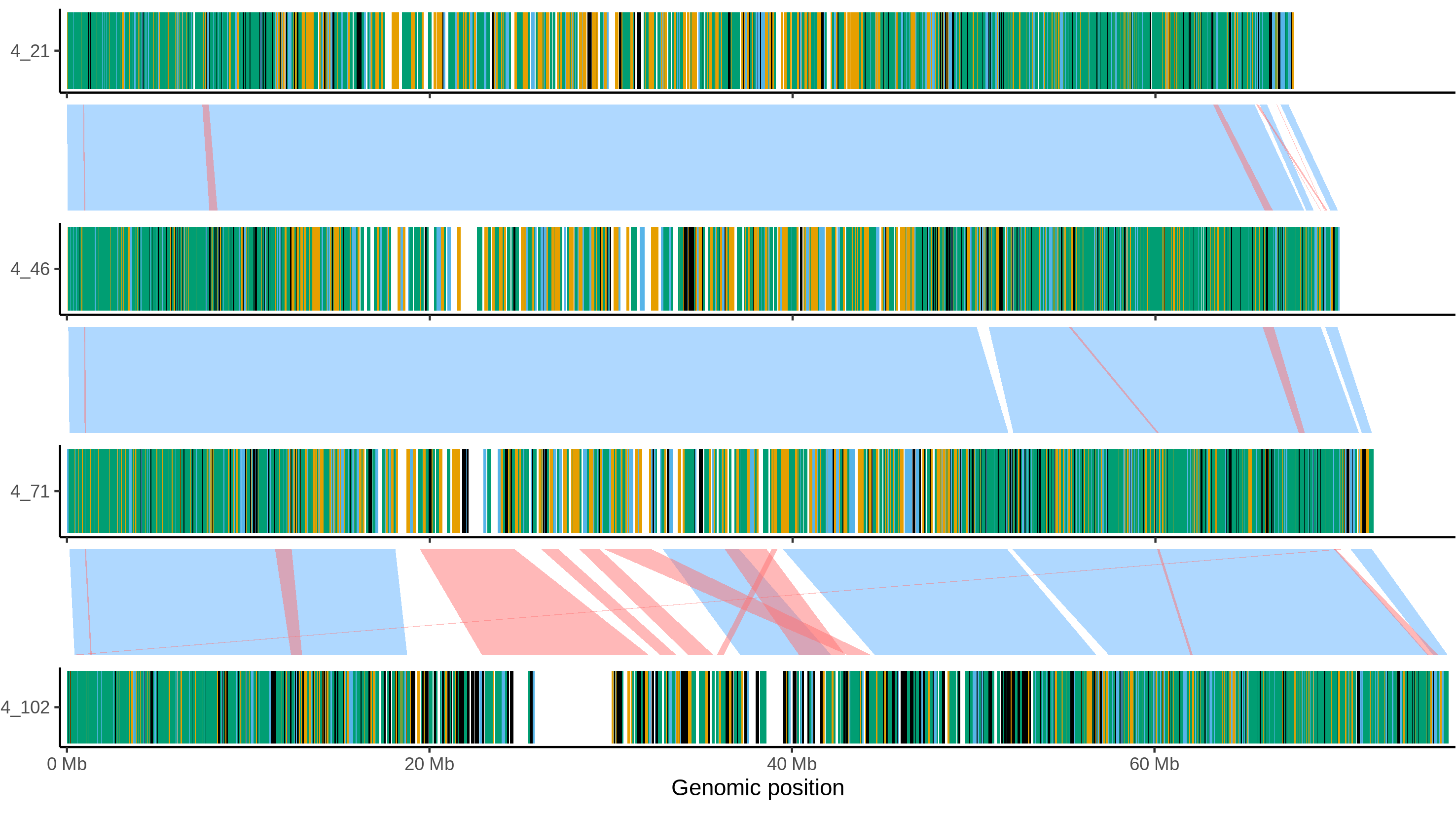

Supplement: Supplementary file 3 — Supplement S3 Supplementary Data. [file PBI-23-874-s002.zip › Supplementary_data/sequence_visualization/Potato/Otava_chr_9.png]

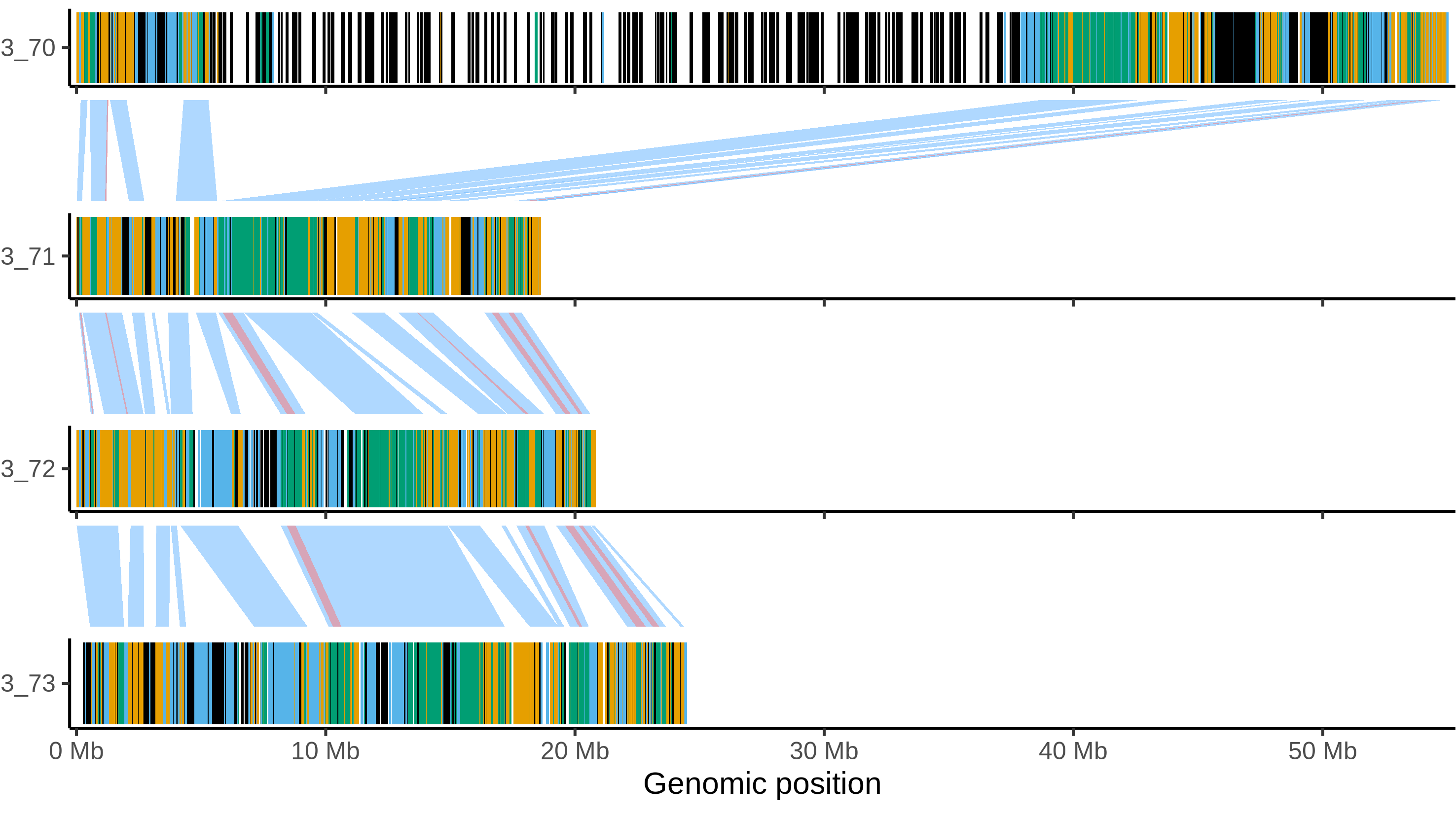

Supplement: Supplementary file 3 — Supplement S3 Supplementary Data. [file PBI-23-874-s002.zip › Supplementary_data/sequence_visualization/Potato/Castle_russet_chr_9.png]

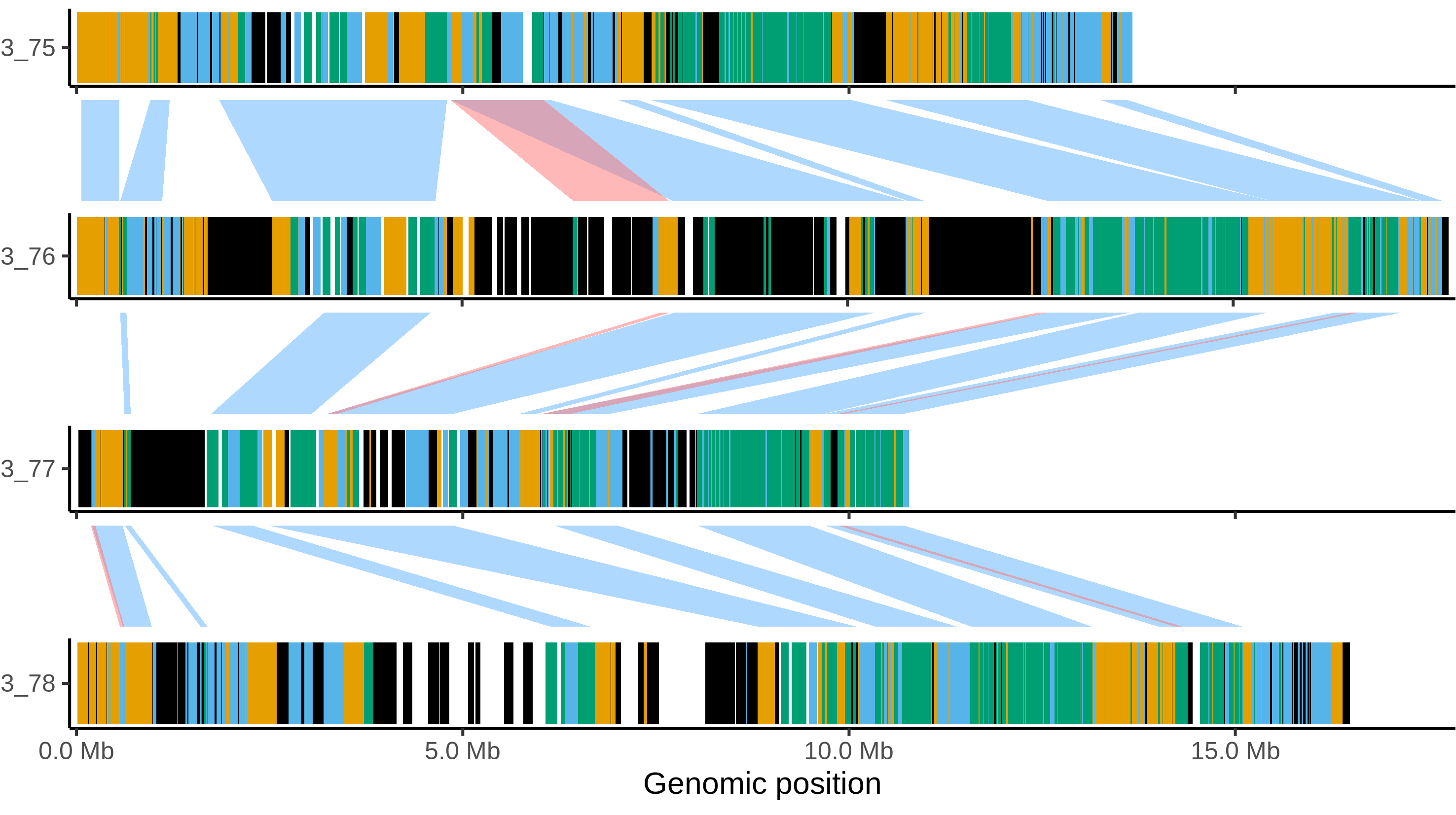

Supplement: Supplementary file 3 — Supplement S3 Supplementary Data. [file PBI-23-874-s002.zip › Supplementary_data/sequence_visualization/Potato/Castle_russet_chr_10.png]

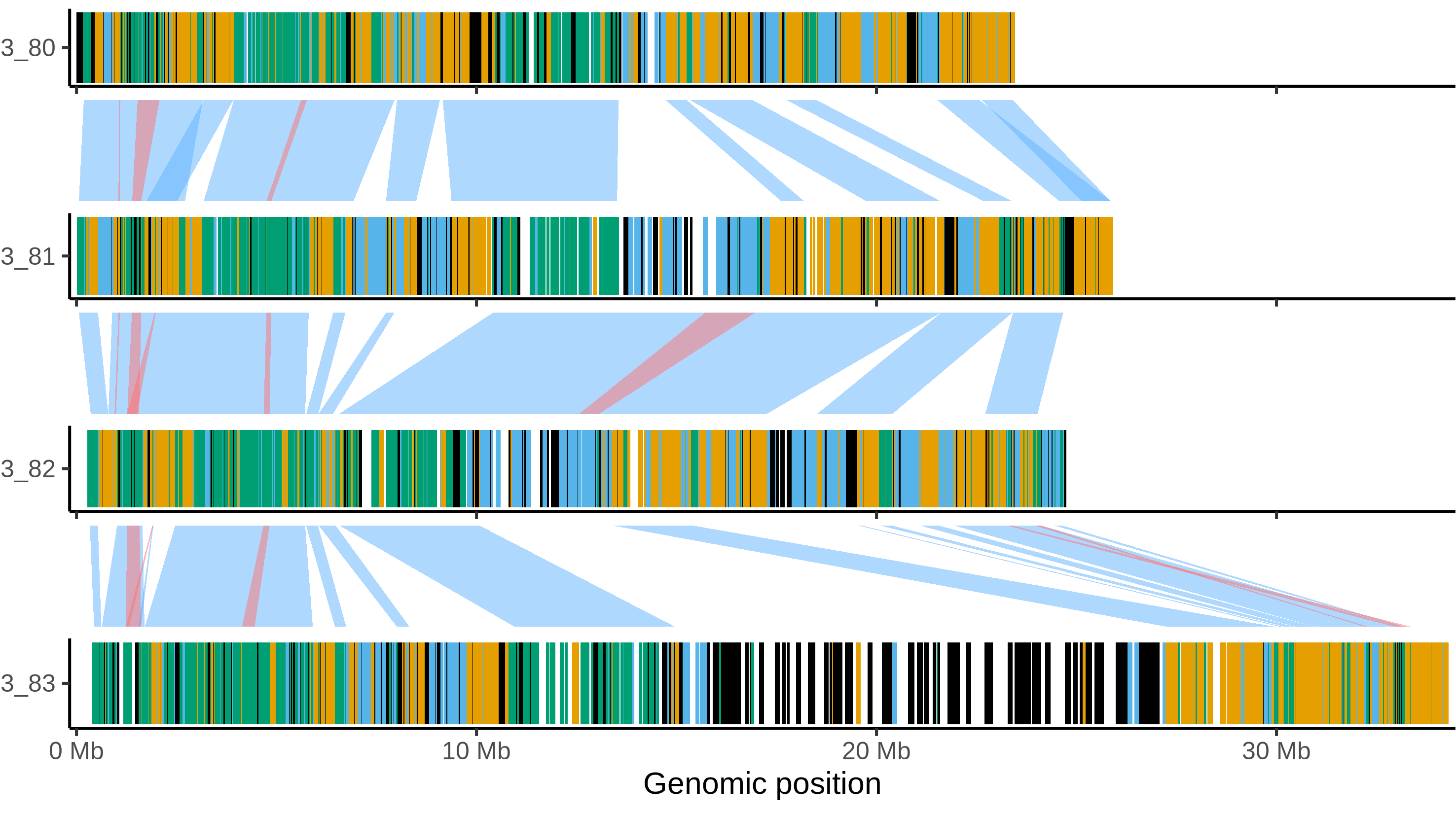

Supplement: Supplementary file 3 — Supplement S3 Supplementary Data. [file PBI-23-874-s002.zip › Supplementary_data/sequence_visualization/Potato/Castle_russet_chr_11.png]

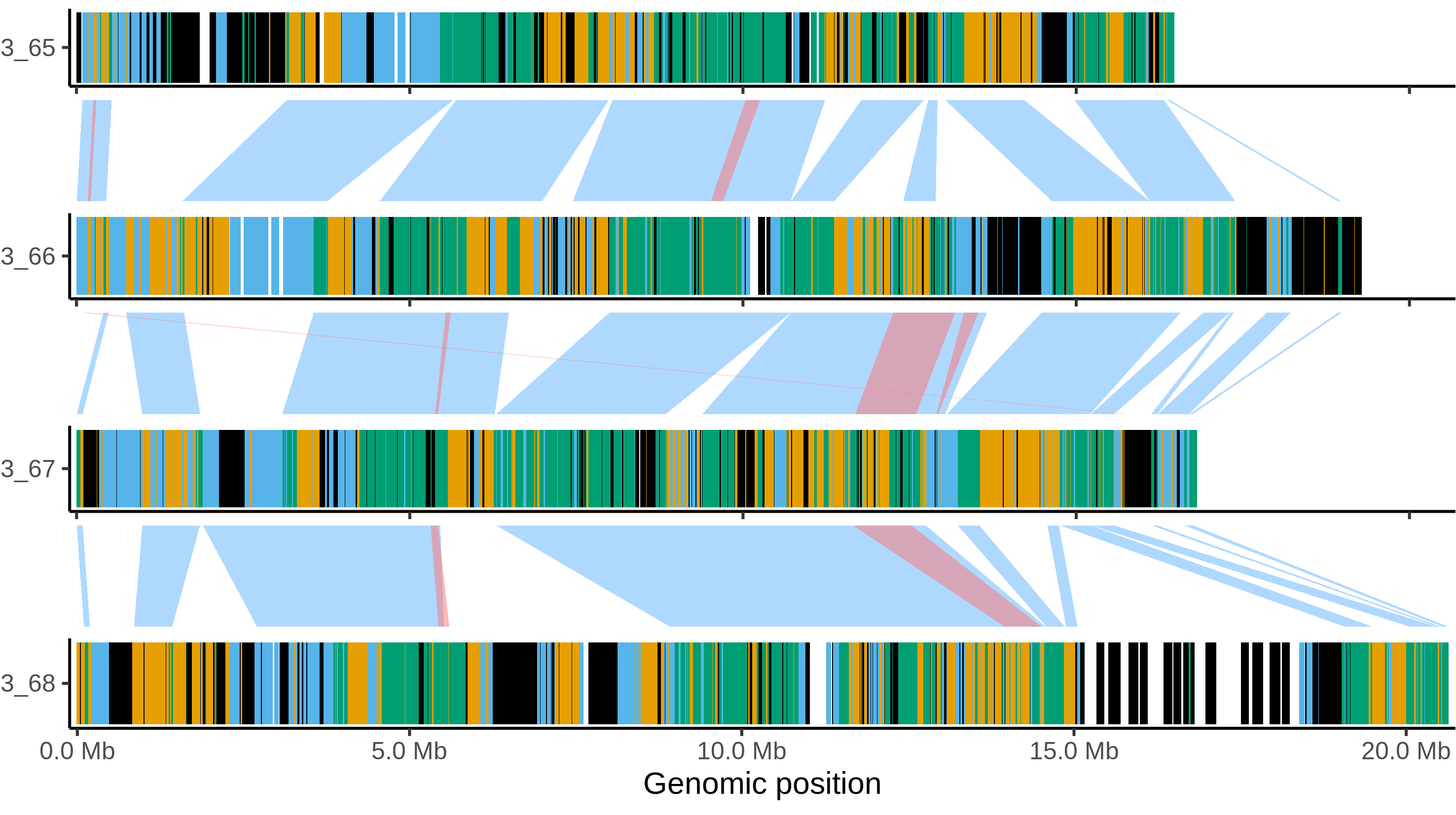

Supplement: Supplementary file 3 — Supplement S3 Supplementary Data. [file PBI-23-874-s002.zip › Supplementary_data/sequence_visualization/Potato/Castle_russet_chr_8.png]

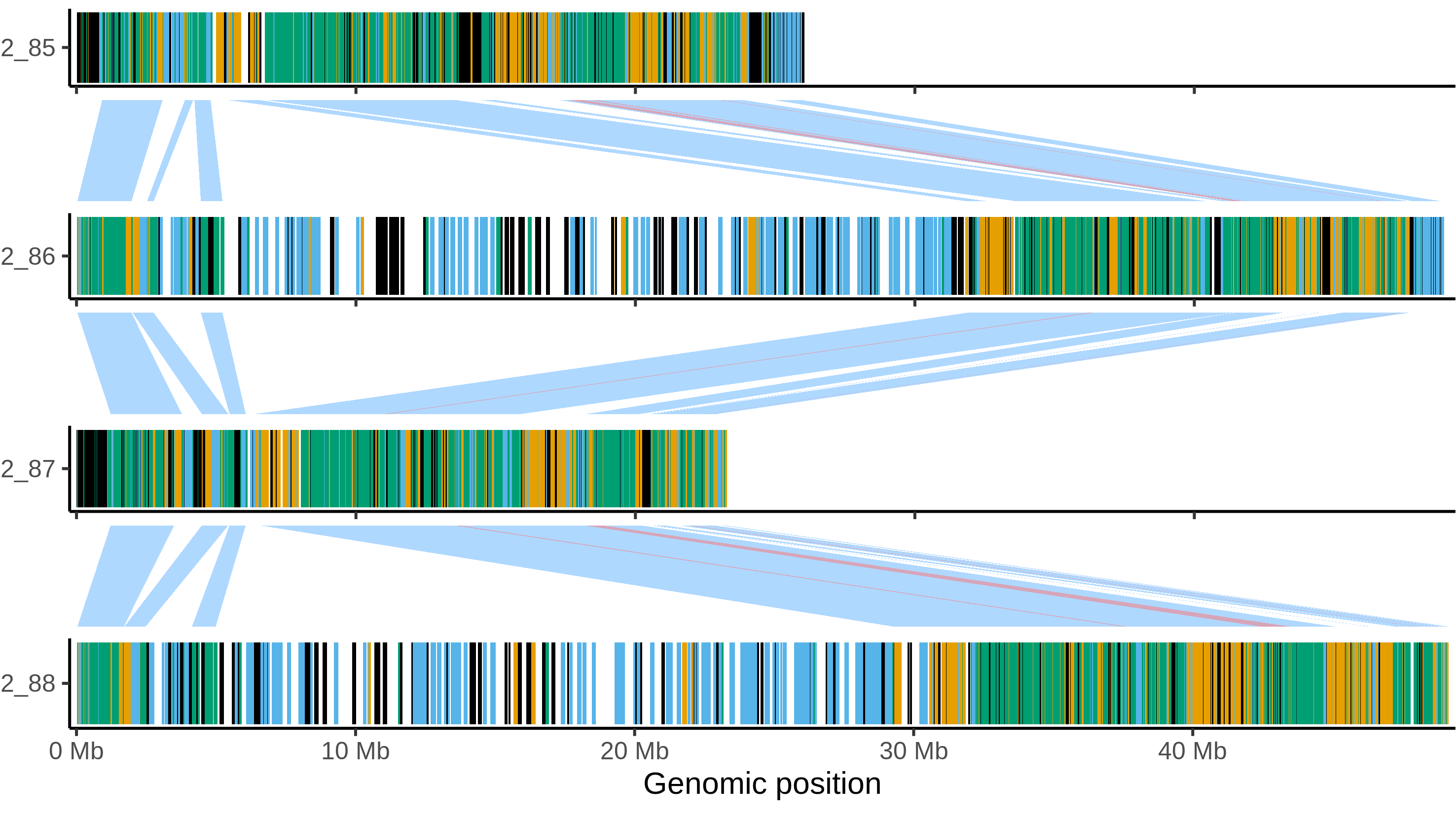

Supplement: Supplementary file 3 — Supplement S3 Supplementary Data. [file PBI-23-874-s002.zip › Supplementary_data/sequence_visualization/Potato/Atlantic_chr_8.png]

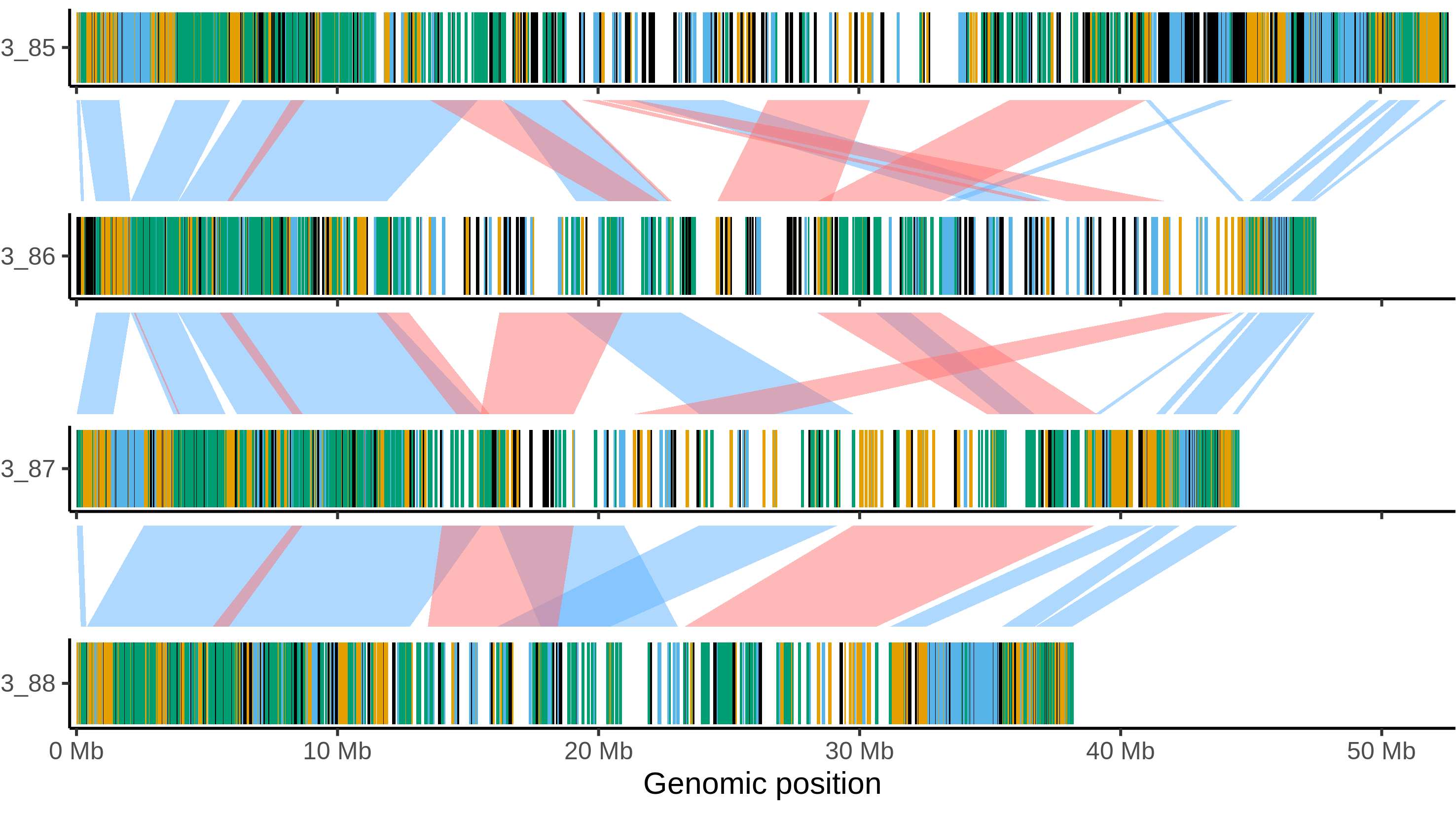

Supplement: Supplementary file 3 — Supplement S3 Supplementary Data. [file PBI-23-874-s002.zip › Supplementary_data/sequence_visualization/Potato/Castle_russet_chr_12.png]

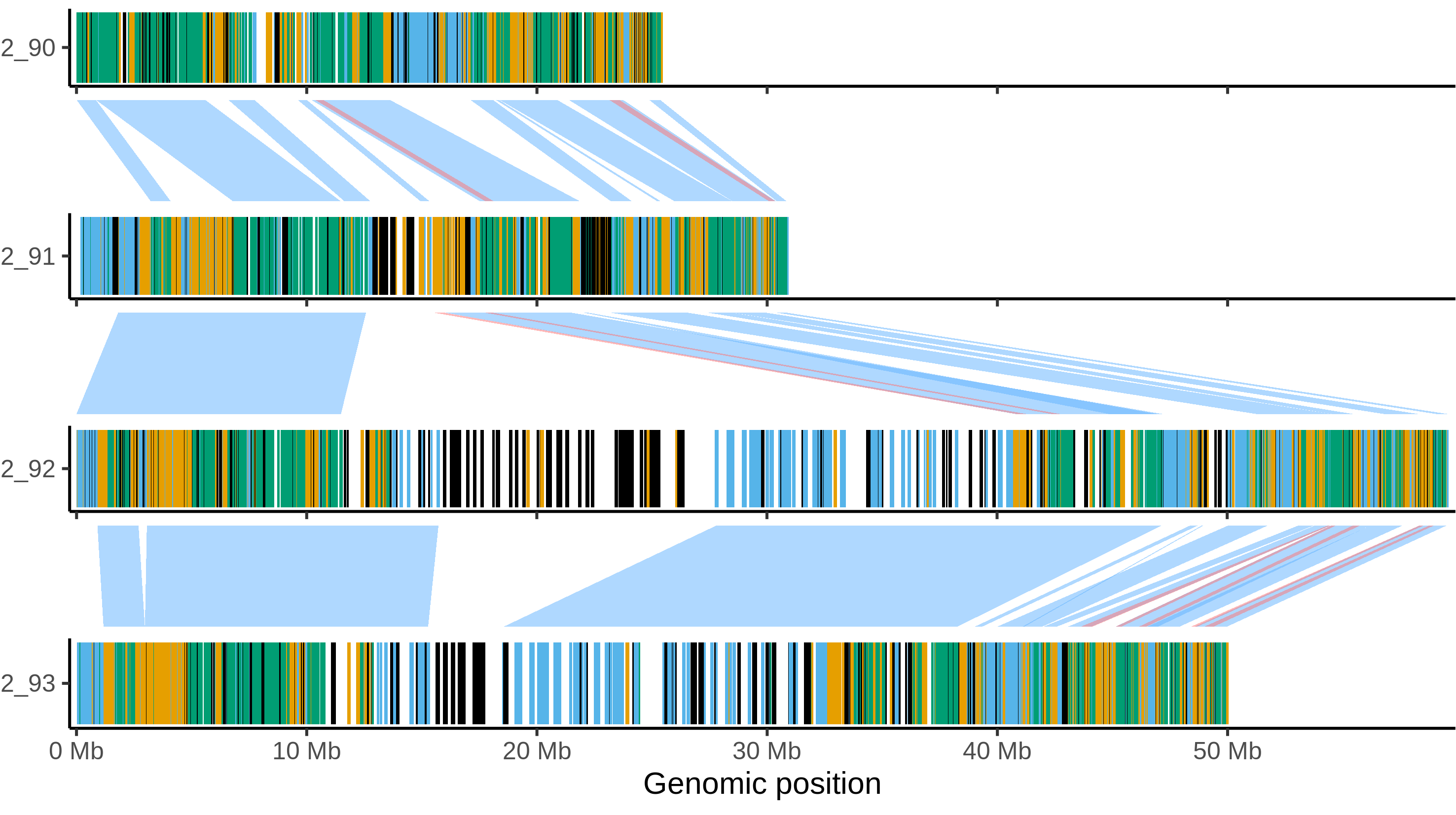

Supplement: Supplementary file 3 — Supplement S3 Supplementary Data. [file PBI-23-874-s002.zip › Supplementary_data/sequence_visualization/Potato/Atlantic_chr_9.png]

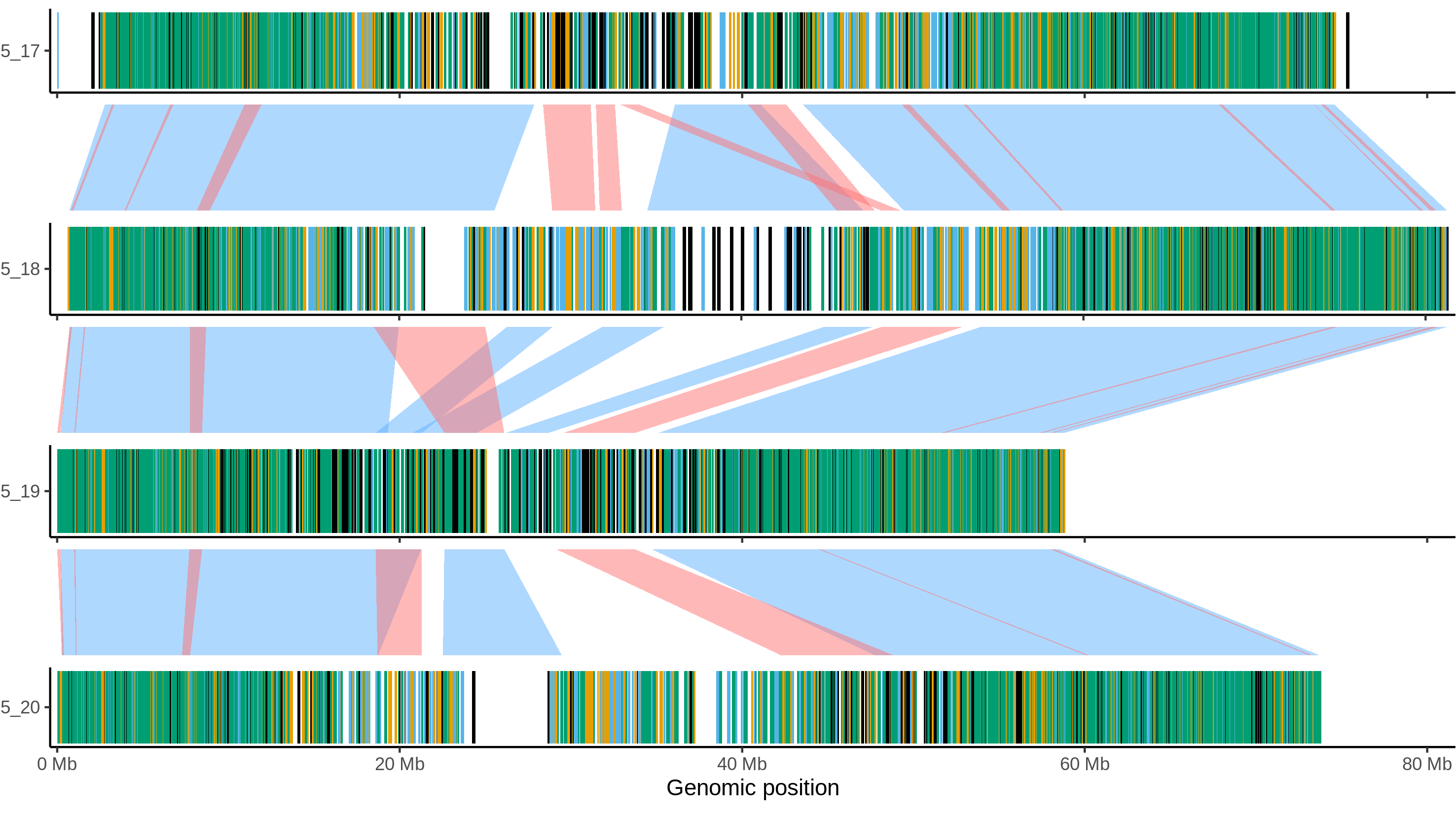

Supplement: Supplementary file 3 — Supplement S3 Supplementary Data. [file PBI-23-874-s002.zip › Supplementary_data/sequence_visualization/Potato/C88_chr_9.png]

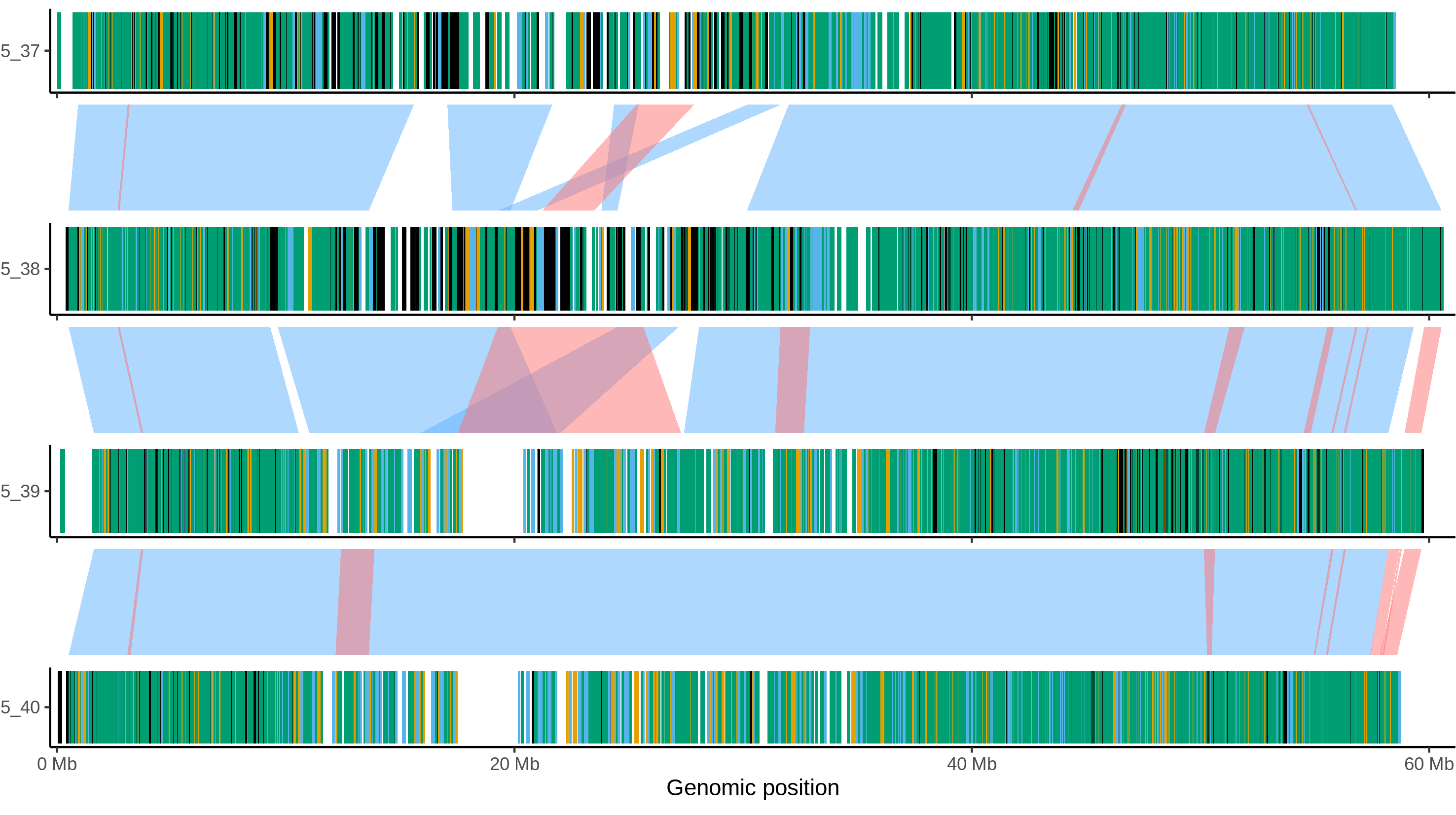

Supplement: Supplementary file 3 — Supplement S3 Supplementary Data. [file PBI-23-874-s002.zip › Supplementary_data/sequence_visualization/Potato/C88_chr_8.png]
